# Subangstrom ion beam engineering of buried ultrathin oxides for scalable quantum computing

**DOI:** 10.1126/sciadv.ads9744

**Published:** 2025-05-07

**Authors:** Nikita S. Smirnov, Elizaveta A. Krivko, Daria A. Moskaleva, Dmitry O. Moskalev, Anastasia A. Solovieva, Aleksei R. Matanin, Vladimir V. Echeistov, Аnton I. Ivanov, Elizaveta I. Malevannaya, Viktor I. Polozov, Evgeny V. Zikiy, Nikita D. Korshakov, Maksim I. Teleganov, Dmitry A. Mikhalin, Nikolai M. Zhitkov, Ruslan V. Romashkin, Igor S. Korobenko, Aleksei V. Yanilkin, Аndrey V. Lebedev, Ilya A. Ryzhikov, Aleksander V. Andriyash, Ilya A. Rodionov

**Affiliations:** ^1^ Shukhov Labs, Quantum Park, Bauman Moscow State Technical University, Moscow 105005, Russia.; ^2^ Dukhov Automatics Research Institute, VNIIA, Moscow 127030, Russia.; ^3^ Institute for Theoretical and Applied Electrodynamics, Moscow 125412, Russia.

## Abstract

Multilayer nanoscale systems incorporating ultrathin tunnel barriers, magnetic materials, amorphous oxides, and promising dielectrics are essential for next-generation logics, memory, quantum, and neuro-inspired computing. Still, an ultrathin film control at the atomic scale remains challenging. Here, we introduce a complementary metal-oxide semiconductor–compatible approach using focused ion beam irradiation for buried ultrathin films’ engineering with subangstrom thickness control. Molecular dynamics simulations confirm the pivotal role of ion-induced crystal defects. Its performance is exemplified by Josephson junction resistance tuning in the range of 2 to 37% with a 0.86% standard deviation in completed chips. Furthermore, it enables ±17-megahertz frequency accuracy (±0.172 angstrom tunnel barrier thickness variation) in superconducting multiqubit processors, as well as qubit energy relaxation and echo coherence times exceeding 0.5 milliseconds.

## Teaser

Focused ion beam irradiation enables precise engineering of buried ultrathin oxide for superconducting qubits’ frequency control.

## INTRODUCTION

Quantum technologies and artificial neural networks, which rely on superconducting qubits (*1*–*3*), magnetic skyrmions (*4*), and next-generation transistors (*5*), are boosting the way to post-exascale hybrid data processing. Multilayer systems with buried 0.5- to 40-nm-thick films are taking center stage in the ever-increasing effort to control nanoscale building blocks for these applications. Statistical variations in Josephson junction (JJ) critical current [with 1.5- to 10-nm-thick AlO_x_ (*6*) or a-Si (*7*) tunnel barriers] of single-flux quantum circuits affect bit error rates and induce storage, decision, and timing errors (*8*). Superconducting quantum processors suffer from two-qubit gate errors and cross-talks (*1*, *9*). As the number of qubits grow, the probability of frequency collisions increases exponentially. Recent superconducting multiqubit processors require qubit frequency set up accuracy ±0.5%, which corresponds to nanoscale JJ normal resistance variation less than 1% (tunnel barrier thickness of 0.5 to 2 nm) (*10*, *11*). When scaling these devices, their nanoscale building block reproducibility becomes crucial and unattainable for state-of-the-art nanotechnology (*12*–*16*). Laser (*10*, *11*), e-beam (*17*), and alternating bias annealing methods (*18*, *19*) were proposed for ultrathin oxide adjusting after fabrication. Their practical applications are limited by nonlocal nature (*10*, *18*), narrow tuning range, or unestablished precision (*17*). A detailed comparison is given in Table 1.

**Table 1.** Josephson junction annealing methods.

Annealing method	Normal resistance tuning range	Resistance reproducibility (σR_N_/<R_N_>)	Qubit frequency reproducibility (σ_f_)	Treatment time per one JJ	Minimal treated area	Coherence impact
Laser annealing (*10*, *11*)	1–15%	±0.61%	±18.5 MHz	8–22 s	4000 × 4000 nm^2^	No
E-beam annealing (*17*)	3%	±1.36%	–	–	10,000 × 10,000 nm^2^	T_1_ decreased
ABAA (*18*, *19*)	80%	±0.37%	±18.4 MHz	1–200 s	Whole isolated metal film	No
This work	1–37%	±0.86%	±17 MHz	1–2 s	20 × 20 nm^2^	T_1_ retention or improvement

The key advantages of the proposed ion beam annealing are nanoscale localization accompanied by extreme accuracy and fast treatment speed. It enables individual sequential treatment of highly integrated nanoscale elements such as junction arrays or superconducting quantum interference devices (SQUIDs), when the irradiated areas can be placed at distances as close as 100 nm. Such a nanoscale, fast, and reproducible superconducting qubit frequency trimming becomes available with the proposed method only. Moreover, next-generation computing platforms require local buried oxide engineering with a subatomic accuracy and chip-scale throughput, together with nondestructive effect on overlying layers of multilayer nanoscale systems.

**Fig. 1.**
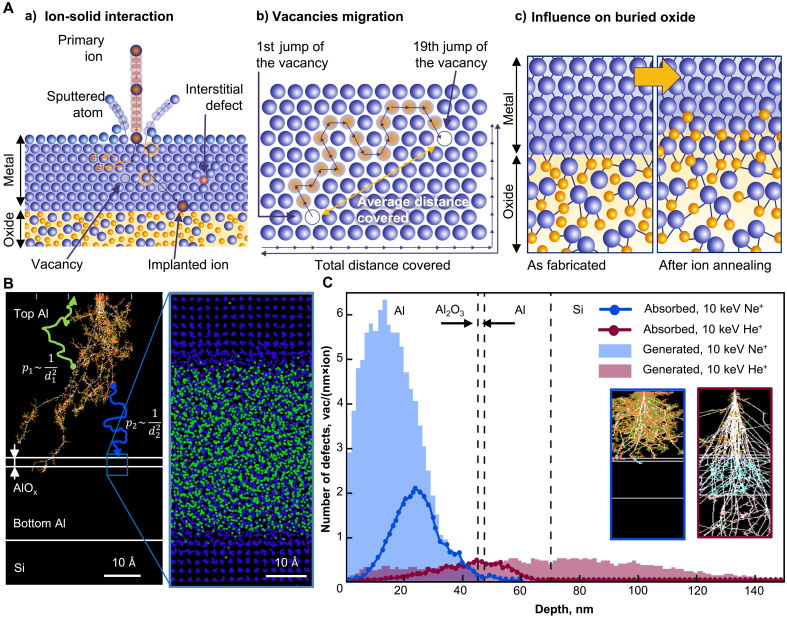
Ion beam engineering of buried oxides: effects of ion irradiation on multilayer nanoscale systems. (**A**) Basic principles: (a) the ion beam interacts with the sample, creating vacancies and interstitial defects; (b) probabilistic defects diffusion toward the oxide interface; (c) crystal defect–induced oxygen diffusion toward metal-oxide interface and oxide formation. (**B**) SRIM (left) and MD (right) simulations for 10 keV Ne^+^ irradiation of the Al/AlO_x_/Al nanoscale system. Left: Ne^+^ creates interstitial defects and vacancies, which migrate to the upper or lower interfaces with probabilities dependent on the distance to the interfaces. Right: Defects and vacancies reaching the oxide interface induce oxide recrystallization. (**C**) Depth distribution of the generated and absorbed defects after 10 keV Ne^+^ and He^+^ irradiation. Absorbed defects are calculated based on the depth distribution of generated defects (SRIM simulations): the sum of all ion ranges over the surface, top and bottom metal-oxide interfaces.

In our approach, we ensure subangstrom accuracy oxide growth buried within a multilayer nanoscale system by defect-induced annealing of top oxide interface with a ~3-nm spot focused ion beam (FIB). Meanwhile, it allows sub–5-nm patterning by scanning the desired systems along a specified trajectory (a given topology). One can select the desired layer in the stack by depth, which is controlled with ion acceleration voltage, and the growth thickness determined by the radiation dose with subangstrom precision (Fig. 1, A and C). Our physical model of the oxide ion beam annealing beneath the top metal is based on molecular dynamics (MD) simulations of ion impact on material structure. We experimentally investigate the effect of ion radiation dose on the room-temperature resistance of JJs and demonstrated 0.86% reproducibility over a 25 mm–by–25 mm chip. We next measure a number of multiqubit quantum processors in a cryogenic environment and guarantee qubit frequency trimming accuracy ±17 MHz using postfabrication ion beam annealing. Moreover, we confirmed that it does not affect transmon qubit performance maintaining energy relaxation time and echo coherence over 0.5 ms. The proposed method can be highly effective in scaling superconducting quantum processors and memory (*20*), quantum-limited amplifiers (*21*, *22*), quantum radars (*23*), powerful neuromorphic computing networks (*24*–*26*), low-power integrated circuits (*27*), sensitive biosensors and gas sensors (*28*), next-generation logic (*4*, *5*, *29*), and memory devices (*30*, *31*).

## RESULTS

### Ion irradiation–induced crystal defect formation

**Fig. 2.**
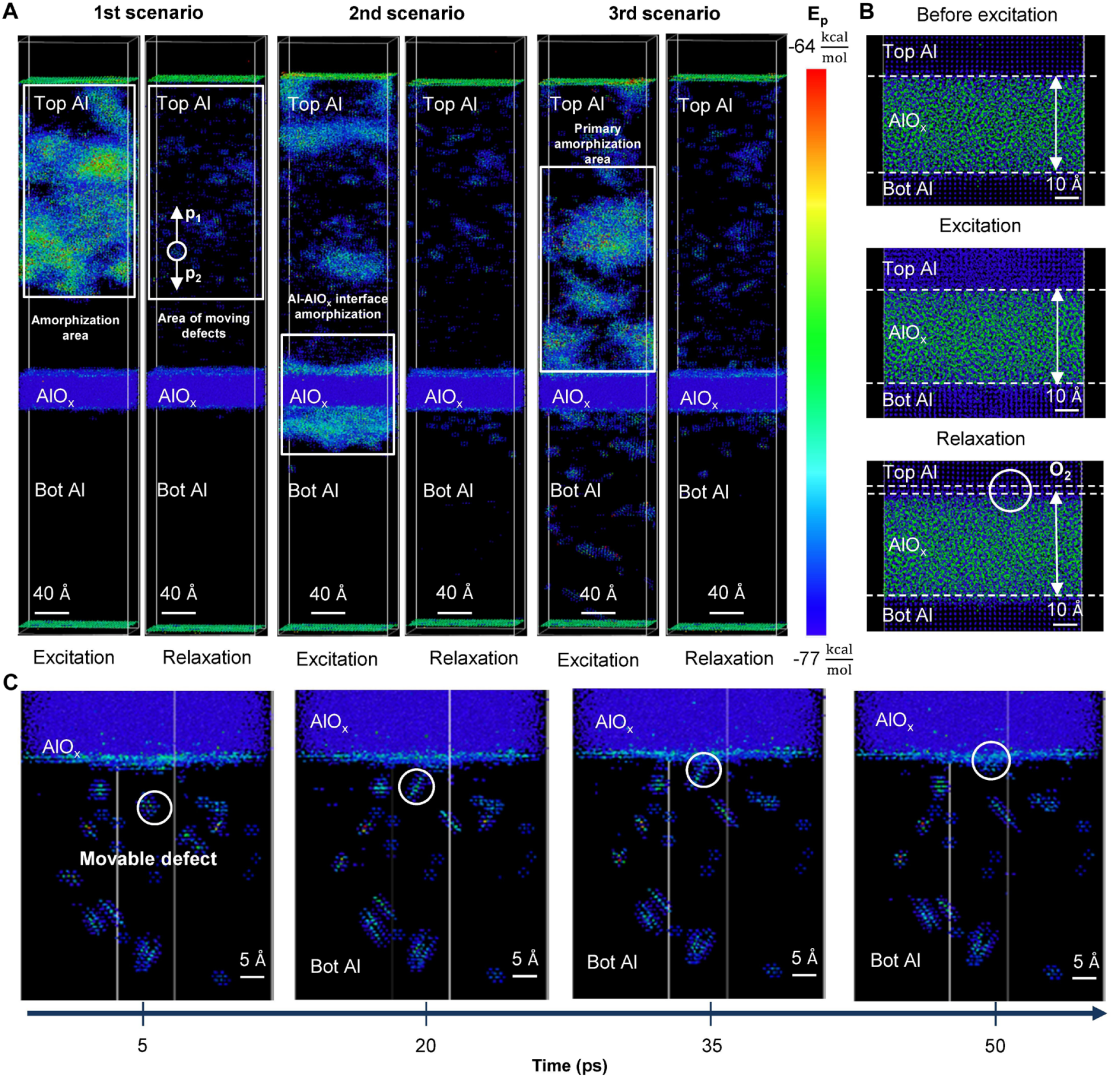
MD simulation of Ne^+^ irradiation on Al/a-AlO_x_/Al (37.0/3.8/19.0 nm) with 8.3 × 8.3 nm^2^ cross section and 0.5 fs time resolution. (**A**) Characteristic scenarios of ion interaction with the Al/a-AlO_x_/Al structure are shown: ion deceleration in the top Al, the tunnel barrier, and the lower Al. It is evident that the amorphization region and the majority of defects form predominantly in the top Al and the tunnel oxide in all cases. (**B**) The oxide and its interface with the top Al at the initial moment, after the displacement cascade, and postrelaxation. Oxide changes are observed in the Al-AlO_x_ interface after the relaxation of ion-induced defects. (**C**) Interstitial defect absorption at successive time points: the interstitial defect approaching the interface and its subsequent absorption.

Numerical simulations using the Stopping and Range of Ions in Matter (SRIM) Monte Carlo approach (*32*) (Fig. 1B) and MD simulations (Fig. 2) confirmed that oxide layer growth is driven by the diffusion of radiation-induced defects to metal-oxide interfaces. Incident ions form a collision cascade along their trajectories, leading to materials amorphization followed by rapid recrystallization. Consequently, defects are generated that slowly diffuse toward interfaces, where they subsequently relax, resulting in material restructuring along the interfaces. For example, one incident 10-keV Ne^+^ generates 150 defects (vacancies and interstitials) in an Al/a-AlO_x_/Al nanoscale system, while one 10-keV He^+^ generates only 50 defects (Fig. 1C). At room temperature, these defects can diffuse for a distance of up to 100 nm. According to MD simulations, interstitial defects are more mobile in the tens of picoseconds timescale. Vacancies have a lower diffusion coefficient (1 to 100 nm^2^/s), depending on the calculation method (*33*). Therefore, vacancies remain immobile over the microsecond timescale but may reach interfaces over seconds to minutes. In our Al/a-AlO_x_/Al system, mobile defects in the top aluminum layer have two relaxation pathways (Fig. 1B): migration to the surface (green trajectory) or oxide interface (blue trajectory). The probability of these processes is inversely proportional to the square of the distance to the interfaces (*34*): $p_{\text{abs}}∼\frac{1}{d^{2}}/\sum \frac{1}{d_{i}^{2}}$. When a defect reaches the interface, it triggers local atomic structure reconfiguration, as confirmed by MD calculations (Fig. 2, B and C, and section S2). The more defects reach the metal-oxide interface, the greater the transformations in the oxide layer. The change in tunnel JJ resistance is proportional to the ion dose; however, saturation may occur beyond a certain dose. We performed MD simulations to precisely analyze the mechanism of high-energy Ne^+^ interaction with the Al/a-AlOx/Al nanoscale system containing a buried ultrathin tunnel oxide. These simulations revealed three characteristic scenarios of ion impact and propagation (section S2). Besides the defect-induced growth scenario, we explored alternative scenarios and concluded that they have a negligible effect on buried oxides (section S4).

#### Scenario 1

The ion does not reach the oxide due to a strong collision with an aluminum atom [Fig. 2A (1st scenario)]. As a result, an amorphous region and the majority of defects form in the middle of the top aluminum layer. After the amorphous region recrystallization, numerous defects remain in the form of vacancies and interstitial clusters, which are mobile at room temperature over the MD simulation timescale.

#### Scenario 2

The ion reaches the oxide layer, where most of its energy is released [Fig. 2A (2nd scenario)]. This causes amorphization of both the top Al layer and the oxide layer. Figure 2B shows the state of the oxide layer at various times before and after the cascade, as well as postrelaxation. We hypothesize that a small amount of unbound oxygen from the oxide layer diffuses into the aluminum. However, within 5 to 20 ps, annealing occurs, leading to aluminum recrystallization. Consequently, oxygen does not substantially diffuse into the aluminum, with only a few atoms moving beyond two lattice periods. Most changes occur within one lattice period ~4 Å. Notably, the second scenario also results in a substantial number of defects in the oxide layer (movies S1 and S2). Therefore, it can be concluded that displacement cascade formation in the oxide layer causes the amorphization of the adjacent aluminum layer, leading to local interface restructuring due to defect relaxation and minor oxygen displacement (up to 5 to 10 Å).

#### Scenario 3

The ion penetrates the entire Al/a-AlO_x_/Al structure, stopping in the lower Al layer [Fig. 2A (3rd scenario)]. Consequently, the amorphous region and the majority of defects form in the lower part of the aluminum layer, close to the oxide layer.

These scenarios demonstrate that even a single neon ion generates a substantial number of mobile lattice defects in the metal layers, capable to diffuse toward the oxide. Hence, the interface serves as an efficient sink for crystalline lattice defects. Figure 2C illustrates the absorption process of interstitial defects by the oxide-aluminum interface. Lattice defects at the interface lead to local structural changes, enabling angstrom-scale control over the properties and thickness of ultrathin oxides buried under thin films of other materials using ~3- to 10-nm spot FIB irradiation. Furthermore, by varying the accelerating voltage, dose and ion type, it is possible to precisely target materials buried at the exact depth of multilayer nanoscale systems. With this experimental nature in mind, we name our method the “iDEA” (ion beam–induced defect activation) annealing.

### JJ a-AlO_x_ engineering at the atomic scale

**Fig. 3.**
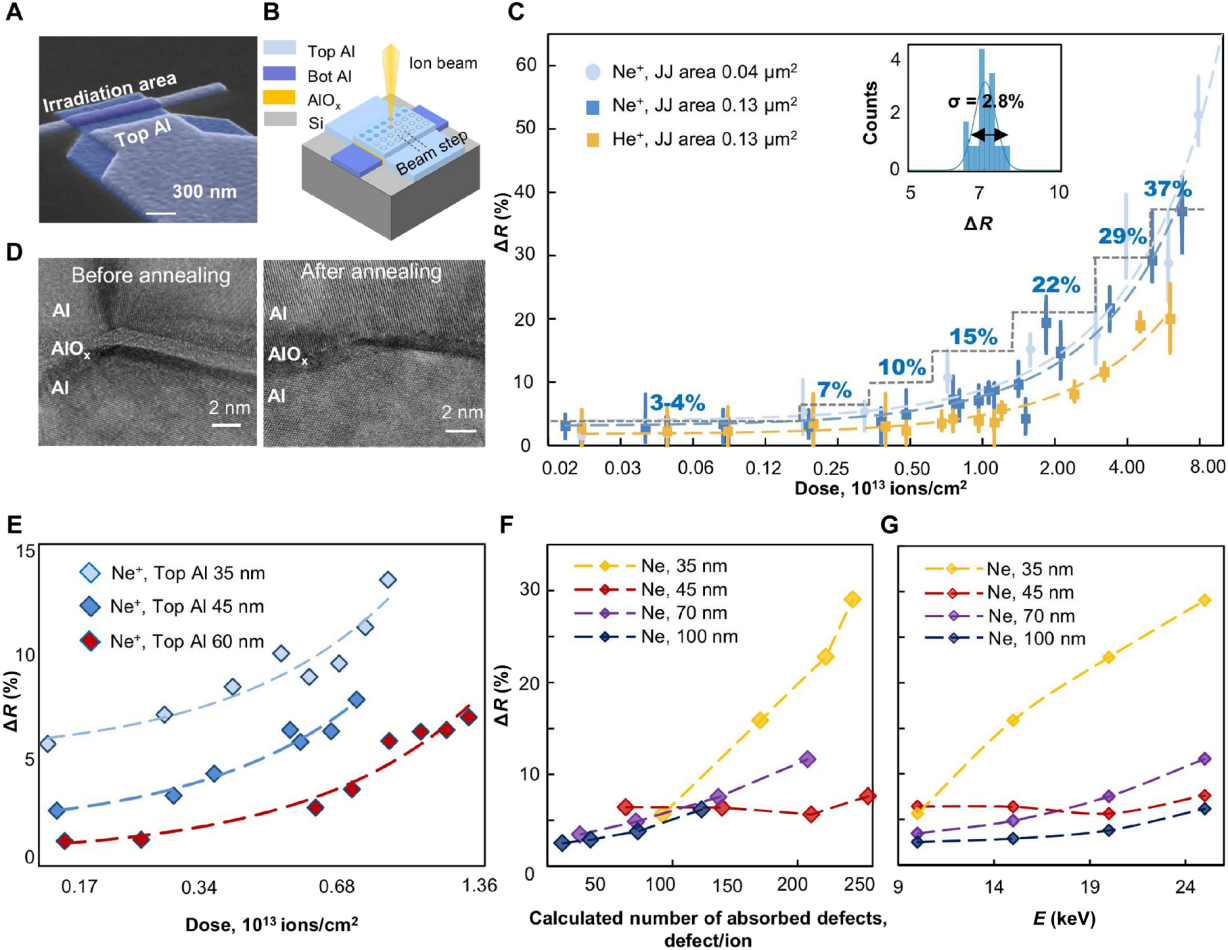
JJ iDEA annealing (areas 0.04 μm^2^ and 0.13 μm^2^). (**A**) Typical Al/a-AlO_x_/Al tunnel junctions test structure. (**B**) Scanning FIB annealing of JJs with a 10-nm spot step (down to 5 Å). (**C**) Experimental curve (logarithmic scale) of room-temperature JJ resistance on 10 keV He^+^ and Ne^+^ irradiation dose. The range of resistance variation exceeds 50%, and the accuracy is independent on junction area and determined solely by irradiation dose. (**D**) TEM images of characteristic top/bottom Al crystallites and tunnel barrier before (left) and after (right) iDEA annealing. No changes in the tunnel barrier crystalline structure are detected. (**E**) Experimental curve of resistance variation on the 10-keV Ne^+^ dose for different top Al layer thickness. Top electrode thickness increasing up to 60 nm reduces the effect of ion irradiation on the tunnel barrier, allowing a tighter resistance control over a wider range of processing doses. (**F**) Experimental curve of resistance variation on the number of absorbed defects for different top Al layer thickness and Ne^+^ accelerating voltages. (**G**) Experimental curve of resistance variation on the Ne^+^ accelerating voltages.

We investigate the influence of FIB irradiation on buried oxides within multilayer nanoscale systems using extremely sensitive Al/a-AlO_x_/Al JJs with ultrathin buried tunnel oxides (0.5- to 3-nm-thick a-AlO_x_) and top/bottom aluminum thin electrodes (15 to 100 nm thick) as a model system (Fig. 3A). The thickness and structure of a tunnel oxide determine its critical current, which is related to the normal resistance *R*_N_ through the Ambegaokar-Baratoff relationship (*35*). We measured JJ critical current at room temperature before and after iDEA annealing. Top/bottom aluminum thickness (45/25 nm) of JJs is typical for superconducting quantum processors with the state-of-the-art JJ area variation ~3% (*12*–*14*). See Materials and Methods for a description of the fabrication process and test JJ structure measurement. Local FIB treatment was then applied to each JJ (Fig. 3B), avoiding the substrate area (Materials and Methods).

The normal resistance was measured immediately after fabrication and iDEA annealing. We estimate the contribution of the leads and the Al layer to the total resistance from 0.1 to 0.6%, because the leads resistance is 25 to 30 ohms, and consider it insignificant.

We experimentally determined the dependence of JJ normal resistance on the irradiation dose of 10 keV Ne^+^ at room temperature (Fig. 3C). The irradiation dose range was chosen to eliminate the sputtering of aluminum atoms (from 0.015 × 10^13^ to 8 × 10^13^ ions/cm^2^), which was confirmed by SRIM calculations and scanning electron microscopy (SEM). For each irradiation dose, 25 identical JJs were annealed and measured (Fig. 3D).

We enable increasing the room-temperature resistance over the wide range of 2 to 37% in a controllable way using Ne^+^, and of 2 to 20% with He^+^. The smaller effect of He^+^ on the buried oxide layer is due to its lower mass and deeper penetration depth (sections S1 and S2). The relative resistance variation is independent on the junction area and is determined solely by the irradiation dose (i.e., the number of defects absorbed by the metal-oxide interface). This dependence is well approximated by a linear function (Fig. 3C) allowing postfabrication JJs critical current tuning with extreme accuracy. Last, we have proved no tunnel barrier crystalline structure degradation (Materials and Methods) for the JJs with the highest irradiation dose for Ne^+^ (8 × 10^13^ ions/cm^2^ at 10 keV) and 36% normal resistance increase after iDEA annealing (Fig. 3D).

Next, we studied the effect of Ne^+^ irradiation with various top Al thicknesses (Fig. 3E), the number of absorbed defects (Fig. 3F), and accelerating voltage (Fig. 3G) to confirm the proposed hypothesis of iDEA annealing mechanism and subangstrom thickness control potential. Decreasing the top Al electrode thickness (constant 10 keV and penetration depth), the effect of Ne^+^ on the tunnel barrier increases (Fig. 2A) due to the reduced distances to the metal-oxide interface and the increased probability of defect absorption by the interface. Next, we determined the dependence of relative resistance variation on the number of absorbed defects. The impact on the oxide was quantified by the number of defects absorbed by top (metal-oxide) and bottom (oxide-metal) interfaces. We found the number of absorbed defects (SRIM simulations) for the experiments with different top Al thicknesses (35, 45, 70, and 100 nm) and various Ne^+^ irradiations (10, 15, 20, and 25 keV) at a fixed dose of 1 × 10^13^ ions/cm^2^. Experimentally, we confirmed the increase in tunnel oxide resistance with the increased number of absorbed defects (Fig. 3F).

### Superconducting multiqubit processor frequency trimming

**Fig. 4.**
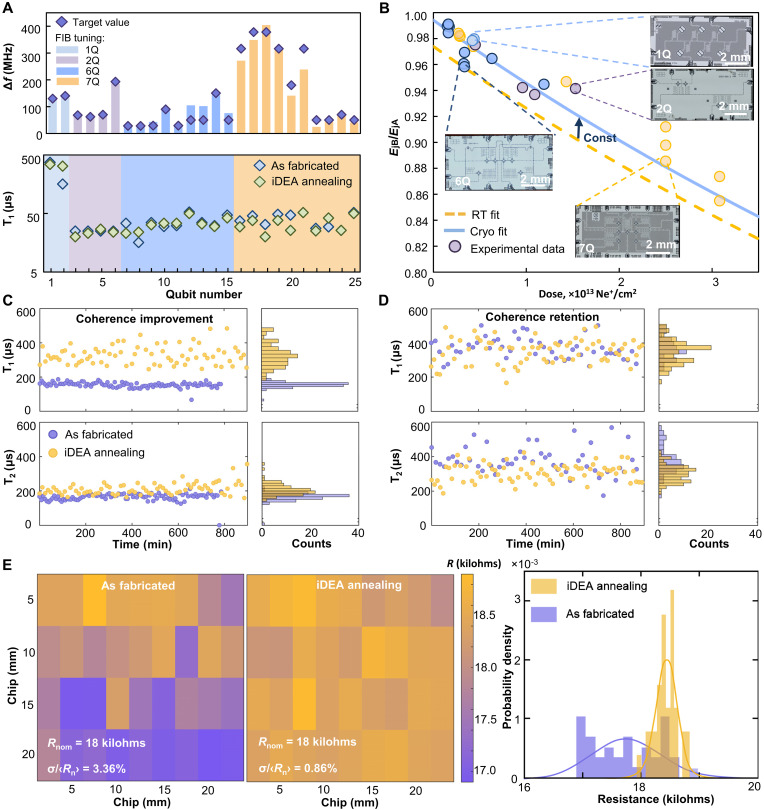
iDEA annealing of superconducting transmons and multiqubit quantum processors. (**A**) Experimental results on the frequency trimming accuracy and iDEA annealing effect on qubits relaxation time (Т_1_). Different qubit circuits represented in colors: single-qubit (blue), two-qubit (purple), six-qubit (blue), and seven-qubit (yellow). Overall frequency trimming accuracy is σ_f_ = 17 MHz. (**B**) Correlation between transmon Josephson energy shift and irradiation dose based on room-temperature resistance measurements (dashed yellow line) and qubits’ cryogenic characterization (blue). The correlation coefficient was 0.78 (the constant shift of JJ resistance between room and cryogenic temperatures). (**C** and **D**) Time-domain measurements of highly coherent qubits before and after iDEA annealing. Both the qubit relaxation improvement from 159 up to 486 μs (C) and retention up to 502 μs (D) are observed after iDEA annealing. (**E**) Distribution maps and probability density histograms of normal resistance *R*_N_ for 0.03 μm^2^ JJs across the 25 mm by 25 mm chip before and after iDEA annealing.

We also evaluated the performance of iDEA annealing in postfabrication frequency trimming of superconducting single transmons and two-, six-, and seven-qubit quantum processors (chips) through nanoscale treatment of individual JJs. This step is crucial to eliminate unwanted qubit frequency collisions and provide proper detuning between interacting qubits for high-fidelity quantum gates. First, we measured the transition frequencies ω_01_ and ω_12_ between energy levels ∣0⟩ → ∣1⟩ and ∣1⟩ → ∣2⟩, respectively (Materials and Methods). A transmon (*36*) qubit functions as a weakly nonlinear harmonic oscillator with a discrete energy spectrum, where the transition frequency between the ground and first excited states is determined by the qubit’s Josephson energy $E_{J}=\frac{\pi \hbar \;I_{c}}{e}$ and its charging energy *E*_C_: ${\hbar \omega }_{01}=\sqrt{8E_{C}E_{J}}-E_{C}$. A transmon charging energy *E*_C_ can be determined with the difference in measured transition frequencies: $E_{C}=\hbar \left(\omega_{01}-\omega_{12}\right)$, which, together with ω_01_, allows determining *E*_J_ and, thus, the normal resistance *R*_N_ using the Ambegaokar-Baratoff relationship. After warming up the chips, we irradiated each JJ of the qubits to get the desired *E*_J_ for the target qubit frequency, using the experimental curve (Fig. 4B). Then, we cooled the chips again and measured frequencies of 25 qubits in different qubit circuits (Fig. 4A). In the case of the seven-qubit circuit (Fig. 4A, yellow), we also tested a two-step iDEA annealing: After the first cool down, the qubit frequency was trimmed by 300 to 400 MHz, followed by precise 50- to 70-MHz tuning after the second cool down.

For all the 25 qubits, we measured relaxation times before and after iDEA annealing (Fig. 4A). For two highly coherent transmons (*37*), relaxation (*T*_1_) and echo coherence (*T*_2*E*_) in time were investigated. One can note (Fig. 4C) a stable improvement in the median energy relaxation time from 159 ± 15 μs to 322 ± 68 μs (up to a maximum of 486 μs) and coherence from 166 ± 22 μs to 217 ± 33 μs (up to a maximum of 356 μs) at 4.385 GHz. We hypothesize that this improvement is due to the detuning of the qubit out of the coupled two-level system (*38*–*40*) after annealing. For the best annealed transmon, the median relaxation time was 345 ± 68 μs (up to a maximum of 502 μs) and coherence was 302 ± 47 μs (up to a maximum of 568 μs) at 4.089 GHz. The average change in relaxation time after iDEA annealing was about 3%. We concluded that iDEA annealing does not adversely affect the coherence of extremely sensitive superconducting quantum multilayer nanoscale systems (Fig. 4, C and D). Notably, for frequency trimming above 150 MHz, the accuracy decreases; however, a multistep iDEA annealing can be used for better precision. Last, we achieved a frequency trimming accuracy of σ_f_ = 17 MHz (Fig. 4B).

Moreover, we have improved the chip-scale variation coefficient of room-temperature resistance $\sigma_{R_{N}}/\left\langle R_{N}\right\rangle$ (JJ nominal resistance of 18 kilohms and area of 0.03 μm^2^) from 3.36% (as fabricated) to 0.86% (corresponding to a critical current variation of 0.87%), which is the best result for JJ yield at the same substrate area (section S3). Assuming the typical 20-Å initial thickness of the tunnel oxide, for the experimentally measured standard deviation of room-temperature resistance of ±0.86%, one can estimate ±0.172 Å accuracy of tunnel oxide thickness across the substrate area.

## DISCUSSION

In summary, we introduce a novel approach for the nanoscale control of buried oxides inside multilayer nanoscale systems with subangstrom precision via FIB irradiation. It allows sub–5-nm patterning through local oxides thickening by scanning with FIB along a specified trajectory (a given topology) inside the multilayer stack. One can select the desired layer in multilayer stack by depth, which is controlled with ion acceleration voltage, and the growth thickness determined by the radiation dose with atomic precision. It involves, first, generating localized crystal defects in the overlying layer above the target oxide using incident Ne^+^ ions, which diffuse through top stack layers in a nondamage manner toward depth-selected layer-to-layer interface. Second, the generated defects slowly diffuse toward interfaces with a probability inversely proportional to the square of the distance to the interfaces. Last, when defects reach the desired film interface, it triggers local atomic structure reconfiguration. Our simulations demonstrate that single ions excite numerous mobile defects capable of diffusing to the oxide boundary and inducing local structural changes.

We experimentally validated an iDEA approach using sensitive Al/a-AlO_x_/Al JJs and quantum nanoscale systems with ultrathin buried tunnel oxide as model systems. We substantiated the proposed annealing mechanism by quantitatively analyzing resistance change as a function of absorbed oxide layer defects. The method enabled controlled adjustment of the tunnel junction normal resistance within the range of 2 to 37% without any structural damage of JJs, and the relative resistance variation remained independent of junction area. The achieved resistance variation, independent of junction area, noticeably improved chip-scale resistance uniformity from 3.36 to 0.86%, with a corresponding critical current variation of 0.87%, which is the best result reported for fabricated JJs with the same nominal resistance and area (18 kilohms and 0.03 μm^2^). We have further confirmed the efficiency of iDEA for postfabrication frequency trimming in superconducting single transmons, as well as in two-, six-, and seven-qubit quantum processors, achieving adjustment accuracy as fine as 0.5%. Crucially, the high quantum conference of superconducting qubits was preserved, with some devices exhibiting improved relaxation times from 159 up to 486 μs and coherence times from 166 up to 356 μs at 4.385 GHz. The best ion beam annealed transmon has retained maximum relaxation times up to 502 μs and coherence times up to 568 μs at 4.089 GHz. The proposed method can be highly effective in scaling superconducting quantum processors and memory (*20*), quantum-limited amplifiers (*21*, *22*), and quantum radars (*23*) and can also be suitable for adjusting fundamentally different devices based on buried thin oxide elements such as powerful neuromorphic computing networks (*41*, *42*), low-power integrated circuits (*27*), and unconventional logic and memory devices (*31*). This approach opens avenues for angstrom-scale control in multilayer nanoscale systems, providing a powerful tool for post-exascale hybrid information processing.

## MATERIALS AND METHODS

### Angstrom-scale ion beam engineering of buried oxide

A primary ion beam with specified energy and current was focused onto the sample, determining the contact spot diameter by selecting the aperture and beam current. Subsequently, the selected area on the sample was scanned using deflecting coils, with scan step and direction controlled by the user. In this study, the scanning algorithm adhered to a fixed topology trajectory resembling a meander pattern. The mechanized stage then moved to the next structure. Processing time for each structure (up to 1 s) depended on the area size under treatment. The processing dose $\text{Dose}=\frac{N}{A}=\frac{I\cdot t}{q\cdot A}$ was defined by the primary ion beam current and exposure time *t*, where *N* represents the number of ions penetrating per unit area *A*, *I* is the FIB current, *t* is the exposure duration, and *q* denotes the ion charge. The primary beam current was determined by the diameter of the aperture and the working gas pressure (2 × 10^−6^ mbar).

### MD simulation

We used a material stack replicating JJs for our simulations. A computational cell comprising an upper aluminum layer (37 nm thick), an aluminum oxide layer (3.8 nm thick), and a lower aluminum layer (19 nm thick) was created. The lateral dimensions were set at 8.3 nm. ReaxFF interatomic potential with short-range part ZBL is used to describe the interactions between atoms. We performed the feasibility analysis of collision cascade in aluminum using MD to check the ReaxFF potential (section S2). The oxide layer was initially amorphized through MD simulations at high temperature, followed by relaxation at 300 K. Subsequently, the oxide layer was interfaced with the aluminum layers. During the relaxation process, the aluminum-oxide boundary formed crystal lattice defects, with some oxygen migrating into the upper aluminum layer, creating a transition layer [Fig. 2A (1st scenario)]. Following structural relaxation, sequential ion irradiation with neon ions was conducted above the upper aluminum layer; each ion imparted a velocity corresponding to 30 keV energy. Each ion irradiation calculation involved two stages: The first stage simulated the passage of a high-energy ion and the creation of a displacement cascade, while the second stage encompassed cascade relaxation, thermalization, recrystallization, and diffusion processes. The first-stage simulations were performed with a variable integration step to resolve high-energy collisions. The second stage used a fixed 0.5-fs integration step over 100,000 steps, equivalent to a total simulation time of 50 ps. Thermalization via interaction with the remaining materials was simulated using thermostating, adjusting the temperature to room temperature over several picoseconds.

### JJ test crystal and fabrication

For this study, we used high-resistivity silicon substrates (10,000 ohm⋅cm). Before the base layer deposition, the substrate is cleaned in a Piranha solution at 80°C, followed by dipping in hydrofluoric bath. A 100-nm-thick Al base layer is deposited using an ultrahigh vacuum e-beam evaporation system. Pads were defined using a direct-laser lithography and dry-etched in BCl_3_/Cl_2_ inductively coupled plasma. The JJs were fabricated using Dolan technique. The substrate is spin coated with a resist bilayer composed of 500 nm of EL9 copolymer and 100 nm of CSAR 62. Layouts were generated and exposed with a 50-keV e-beam lithography system. The development was performed in a bath of amylacetate followed by isopropyl alcohol (IPA) dip and an additional IPA-DIW solution. Al/AlO_x_/Al junctions are shadow evaporated in an ultrahigh vacuum deposition system. Resist lift-off was performed in *N*-methyl-2-pyrrolidone at 80°C. Then, aluminum bandages are defined and evaporated using the same process as for the junctions with an in situ Ar ion-milling to provide good electrical contact of the junction with the base layer. Lift-off is performed in a bath of *N*-methyl-2-pyrrolidone with sonication at 80°C and rinsed in a bath of IPA with ultrasonication. The room-temperature resistances of JJs were individually measured after fabrication and then after ion irradiation with automated probe station.

### TEM, SEM, and EDS characterization of a-AlO_x_ tunnel barriers

During the fabrication of test and experimental samples, SEM was used for quality and uniformity assessment of deposited JJ electrodes. Transmission electron microscope (TEM) samples were prepared by a FIB instrument with a gas injection system. The TEM samples were thinned to electron beam transparency by a Ga^+^ ion beam from 30 to 2 kV. The TEM samples were investigated by an aberration-corrected TEM at 200 kV. A high-angle annular dark-field detector was used for dark-field imaging in scanning TEM mode with a convergent semi-angle and a collection semi-angle of 18 mrad and 74 to 200 mrad, respectively. Energy-dispersive x-ray spectroscopy (EDS) studies were carried out with probe currents of 250 nA.

### Room-temperature normal resistance characterization

The JJ room-temperature resistance were individually measured using a standard technique based on passing current through them, and the voltage drop across the same junction with the automated probe station was measured. Each test chip consisted of an array of 1600 Josephson structures (section S3), and a total of 12,536 such structures were measured. Each test structure consisted of a single Al/a-AlO_x_/Al junction and aluminum contact pads for electrical measurements. Multiple measurements of the same junction have demonstrated repeatability of measurements better than 0.5%.

### Multiqubit processor fabrication

The device is fabricated in a four-step process: (i) base Al layer patterning, (ii) JJ double-angle evaporation and lift-off, (iii) patterning and deposition of bandages, and (iv) air-bridge fabrication. Devices are fabricated on Topsil Global Wafers high-resistivity silicon substrate (ρ > 10,000 ohm·cm, 525 μm). Before the deposition, the substrate is cleaned in a Piranha solution at 80°C, followed by dipping in 2% hydrofluoric bath to remove the native oxide. A 100-nm-thick base aluminum layer is grown using e-beam evaporation in a ultrahigh vacuum deposition system. A Dow MEGAPOSIT SPR 955-CM photoresist (600 nm) is then spin coated. Qubit capacitors, resonators, wiring, and ground plane are defined using a laser direct-writing lithography system, developed in AZ Developer to minimize film damage and then dry etched in BCl3/Cl2 plasma. The photoresist is stripped in *N*-methyl-2-pyrrolidone at 80°C for 3 hours and rinsed in IPA with sonication. The substrate is then spin coated with a resist bilayer composed of 500 nm methyl methacrylate (MMA) and 300 nm polymethyl methacrylate (PMMA). The development is performed in a bath of MIBK/IPA 1:3 solution followed by rinsing in IPA. Al/AlO_x_/Al JJs are patterned using a 50-keV electron beam lithography system and aluminum electrodes are shadow evaporated in an ultrahigh vacuum deposition system. A 25-nm-thick first Al junction electrode is oxidized at 5 mbar to form the tunnel barrier and next the 45-nm-thick counterelectrode is evaporated. We then pattern and evaporate aluminum bandages using the same process as for junctions with an in situ Ar ion milling to provide good electrical contact of the junction with the base layer. Lift-off is performed in a bath of *N*-methyl-2-pyrrolidone with sonication at 80°C for 3 hours and rinsed in a bath of IPA with sonication. Last, aluminum freestanding crossovers are fabricated using a conventional approach. An SPR 220 3-μm photoresist is spin coated and then the sacrificial layer is patterned using a direct laser writing system. The development is performed in AZ Developer/deionized water solution (1:1) for 2 min to minimize film damaging and the resist is reflowed at 140°C. Al (300 nm) is then evaporated with an in situ Ar ion milling to remove the native oxide. A second layer of 3-μm SPR 220 is used as a protective mask and the excess metal is dry etched in inductively coupled plasma. A damaged layer of photoresist is then removed in oxygen plasma, and both layers of photoresist are stripped with *N*-methyl-2-pyrrolidone at 80°C.

### Cryogenic setup

**Fig. 5.**
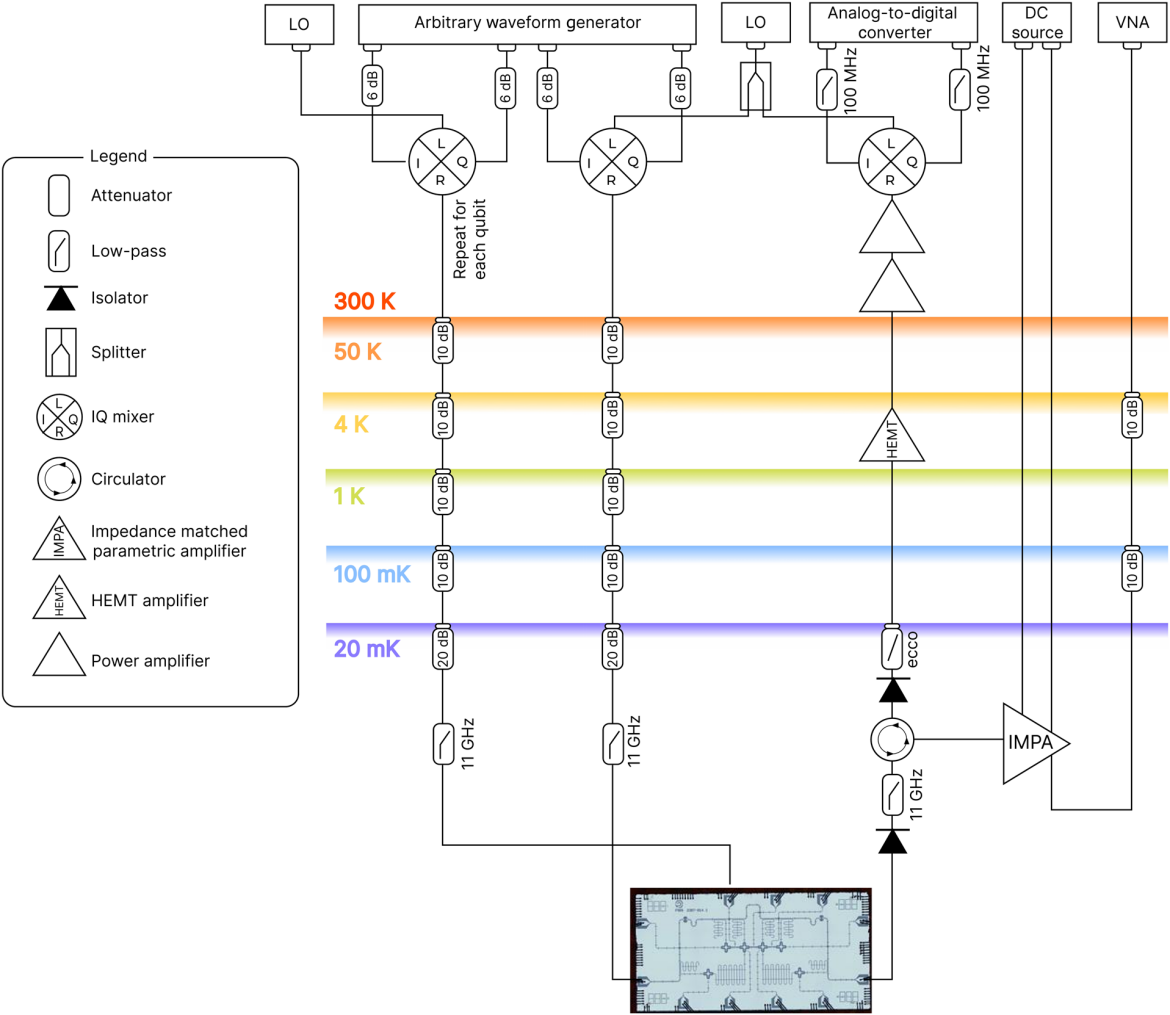
Measurement scheme of superconducting quantum processors in a dilution refrigerator.

The detailed experimental setup scheme is shown in Fig. 5. The device is measured in a Bluefors LD400 dilution refrigerator. One line connected to the chip is used for readout and the others are used for applying single-qubit gates (XY controls). Pulsed XY control of the qubits was realized by upconverting the intermediate-frequency in-phase and quadrature signals from the arbitrary waveform generator (AWG), using an IQ mixer and a microwave local oscillator.

Readout tone was generated by AWG and upconverted to the readout resonator frequency using a mixer and a microwave local oscillator. A readout microwave signal that passed through the chip is amplified by a cryogenic impedance matched parametric amplifier (IMPA) and then downconverted. The readout signal is also amplified by high–electron mobility transistors at the 4K stage of the cryostat and at room temperature. We use a dc source and superconducting coil located on the packaging to tune up the parametric amplifier to the desired frequency and pump the IMPA by a microwave source of vector network analyzer. Readout line is additionally equipped with a custom-made Eccosorb filter on the cryostat mixing stage to suppress infrared noise and standing waves. Sample holders with IMPA is placed in the magnetic shield (*43*–*48*)*.*

## Supplementary Materials

### The PDF file includes:

Supplementary Materials and Methods Supplementary Text Figs. S1 to S25 Tables S1 to S10 Legends for movies S1 and S2 References
