# Supplementary material for: Subangstrom ion beam engineering of buried ultrathin oxides for scalable quantum computing
Source: Sci Adv. 2025 May 7;11(19):eads9744. doi: 10.1126/sciadv.ads9744 (PMC12057654; doi:10.1126/sciadv.ads9744)
Supplement: Supplementary file 1 — Supplementary Materials and Methods Supplementary Text Figs. S1 to S25 Tables S1 to S10 Legends for movies S1 and S2 References [file sciadv.ads9744_sm.pdf]

Science Advances
AAAS

Supplementary Materials for

Subangstrom ion beam engineering of buried ultrathin oxides for scalable quantum computing

Nikita S. Smirnov *et al.*

Corresponding author: Ilya A. Rodionov, irodionov@bmstu.ru

Sci. Adv. **11**, eads9744 (2025)
DOI: 10.1126/sciadv.ads9744

The PDF file includes:

Supplementary Materials and Methods
Supplementary Text
Figs. S1 to S25
Tables S1 to S10
Legends for movies S1 and S2
References

Other Supplementary Material for this manuscript includes the following:

Movies S1 and S2

Supplementary Text

Section 1: SRIM simulations details

The first estimation of the effect of the ion beam on the Josephson junction (JJ) was performed by simulating the collision of ions with a solid in the SRIM programme. In SRIM, the multilayer target structure corresponding to the JJ material (Al-Al_2O_3-Al) and the parameters of the ion beam were specified.

SRIM performs calculations for one ion at a time to accurately estimate the physics of each collision between an ion and a target atom. The type of calculation used in the modelling was surface sputtering/monolayer collisions. The accuracy depends on the number of ions. Simulations were performed for a beam of 1000 ions, although the actual number of ions affecting the JJ is ~100, since a calculation for 1000 ions typically gives an accuracy of better than 10%.

Single JJ processing experiments were performed with Ne and He gases, as shown in Figure 3(d) of the main text. As can be seen from the SRIM simulation, helium ions He+ (Fig. S2) pass through the JJ and stop only at the substrate, whereas for neon Ne+ (Fig. S1) only a small fraction of ions reach the tunnel barrier. The main text of the paper describes the experiment of irradiating Josephson junctions with different top electrode thicknesses. First, the ion penetration depth was analysed for different thicknesses in SRIM. As can be seen from Figures S2, S4, when using helium ions, changing the thickness of the top electrode of the JJ has no significant effect on the processing result because the ions are inhibited in the substrate anyway. However, it is interesting to note that as the energy of the helium ions increases, the number of defects in the tunnel barrier decreases.

The results of experiments on the treatment of Josephson junctions with a neon ion beam are not in such good agreement with SRIM modelling. For cases where ions reach the barrier, the correspondence between experiments and simulations is observed. However, we performed Josephson junction processing experiments for cases where SRIM showed that no ions reached the barrier (Figs. S3, S5) and also observed a few percent increase in resistance, as shown in Figure

3 of the main text. We attribute this to the ability of defects to diffuse toward interfaces over tens of nanometers.

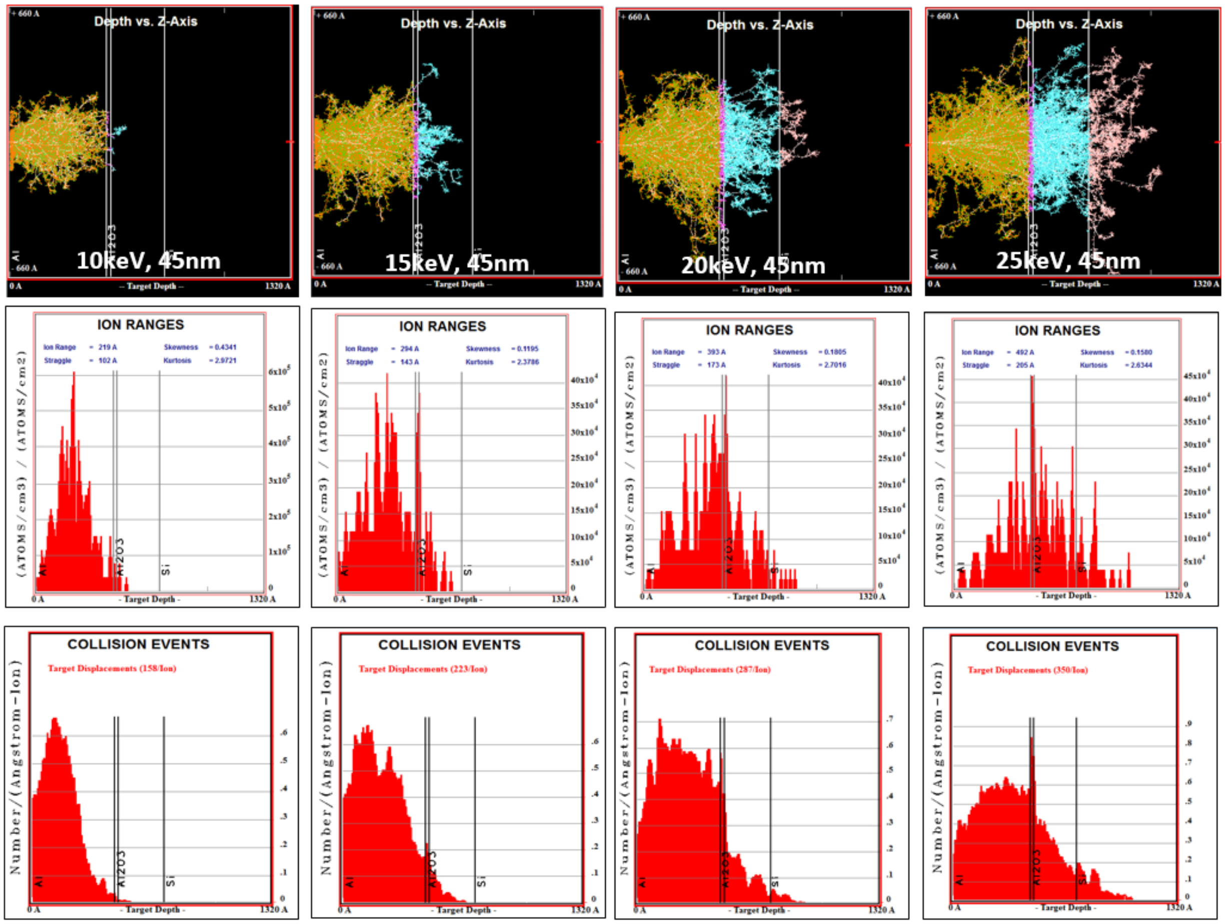

Figure S1 Results of modeling the penetration of Ne^{+} neon ions with an energy of 10-25 keV into a Josephson junction with a top electrode thickness of 45 nm

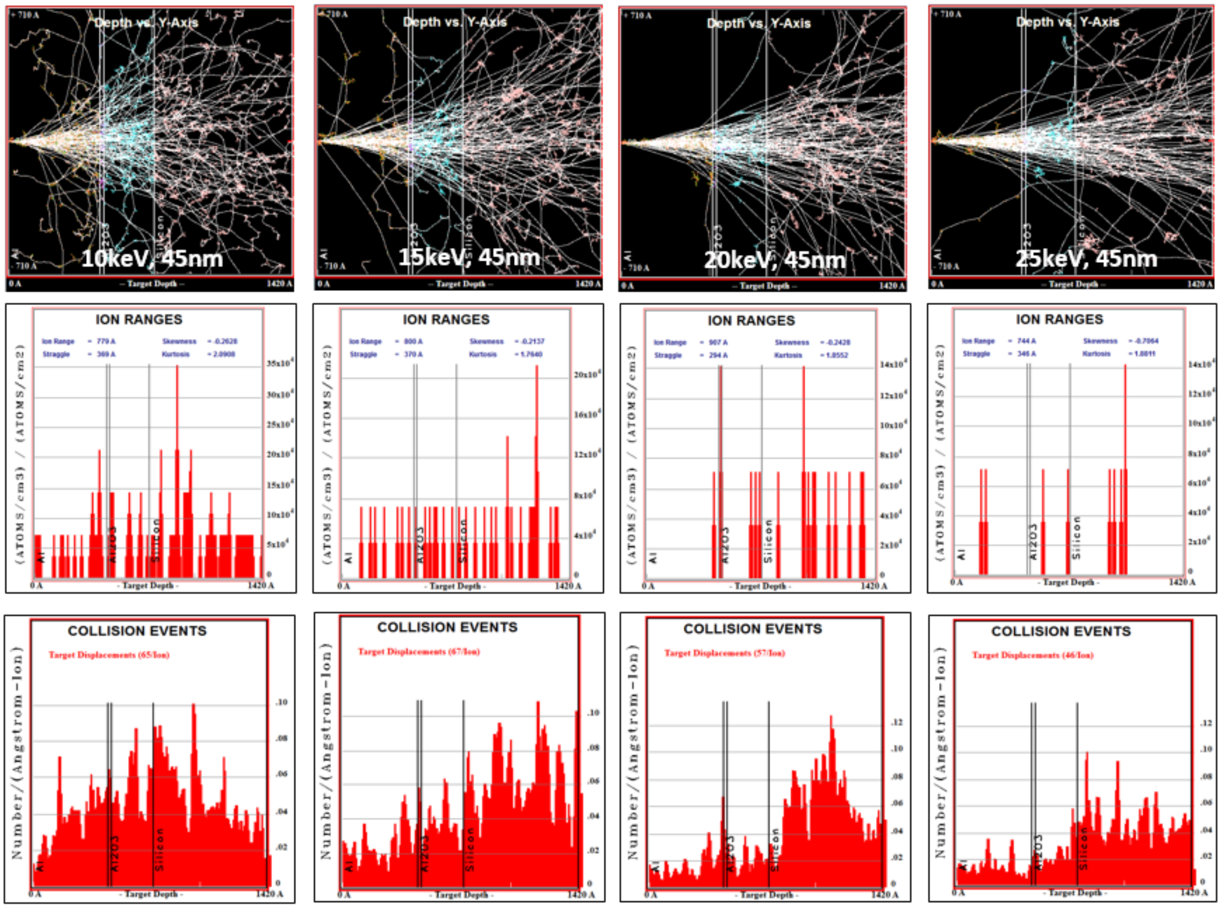

Figure S2 Results of modeling the penetration of He$^+$ neon ions with an energy of 10-25 keV into a Josephson junction with a top electrode thickness of 45 nm

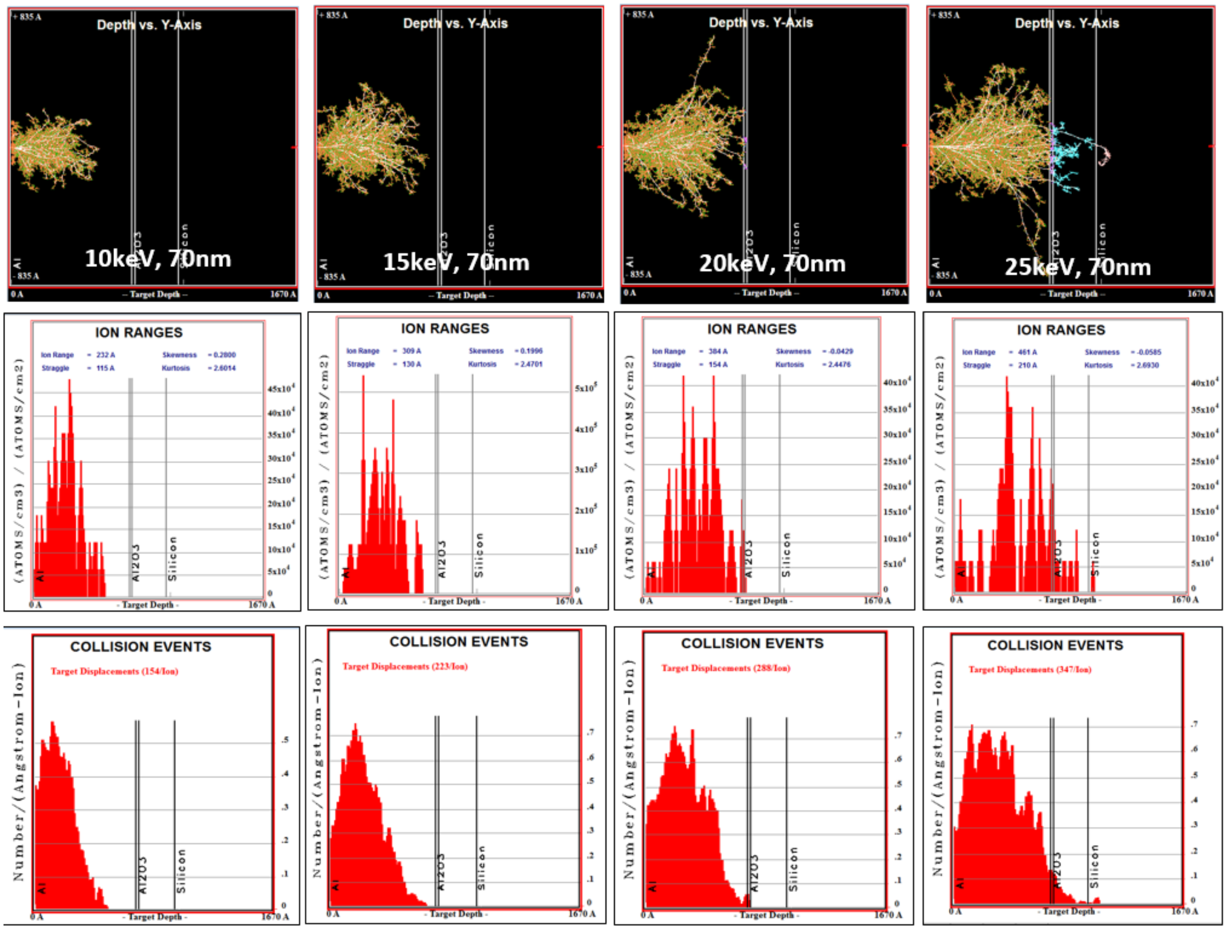

Figure S3 Results of modeling the penetration of Ne^{+} neon ions with an energy of 10-25 keV into a Josephson junction with a top electrode thickness of 70 nm

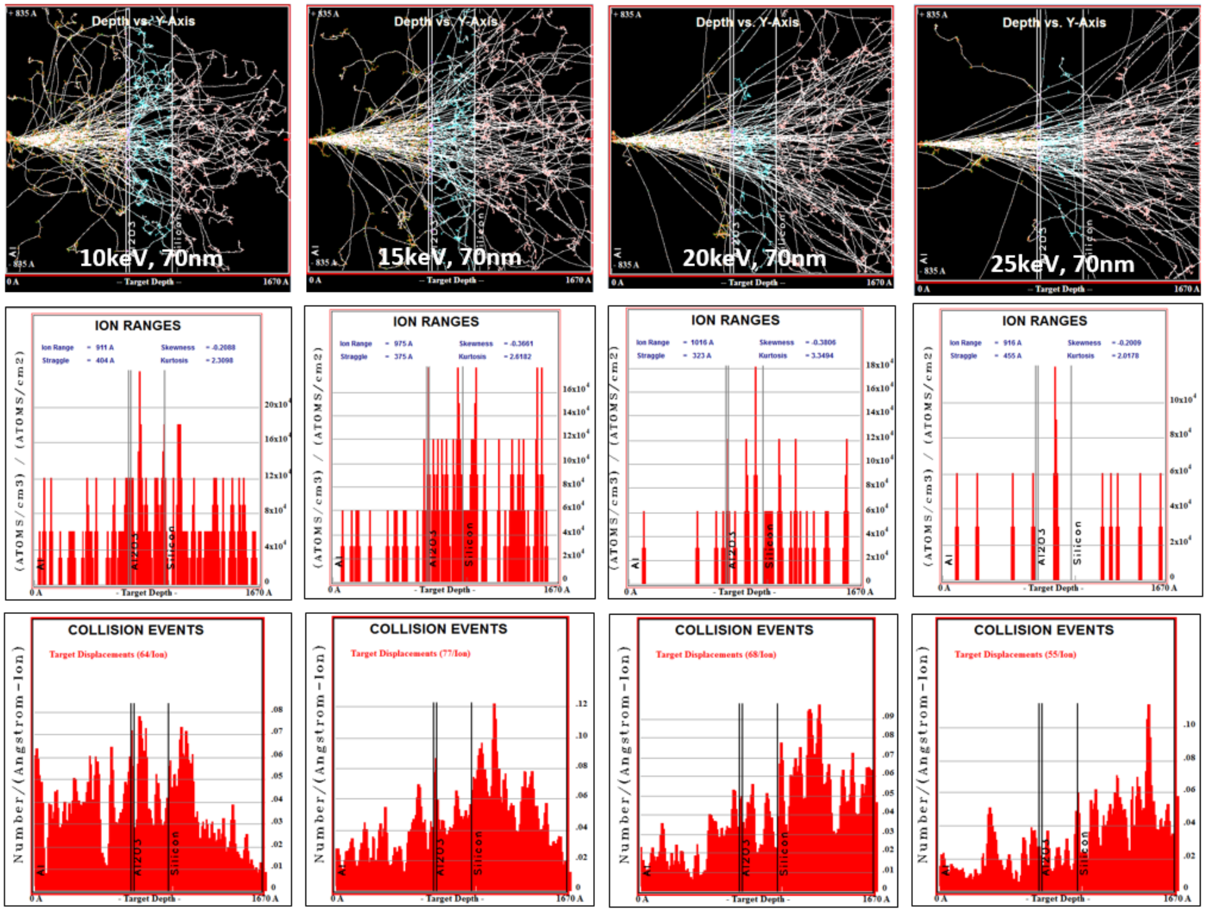

Figure S4 Results of modeling the penetration of He^{+} neon ions with an energy of 10-25 keV into a Josephson junction with a top electrode thickness of 70 nm

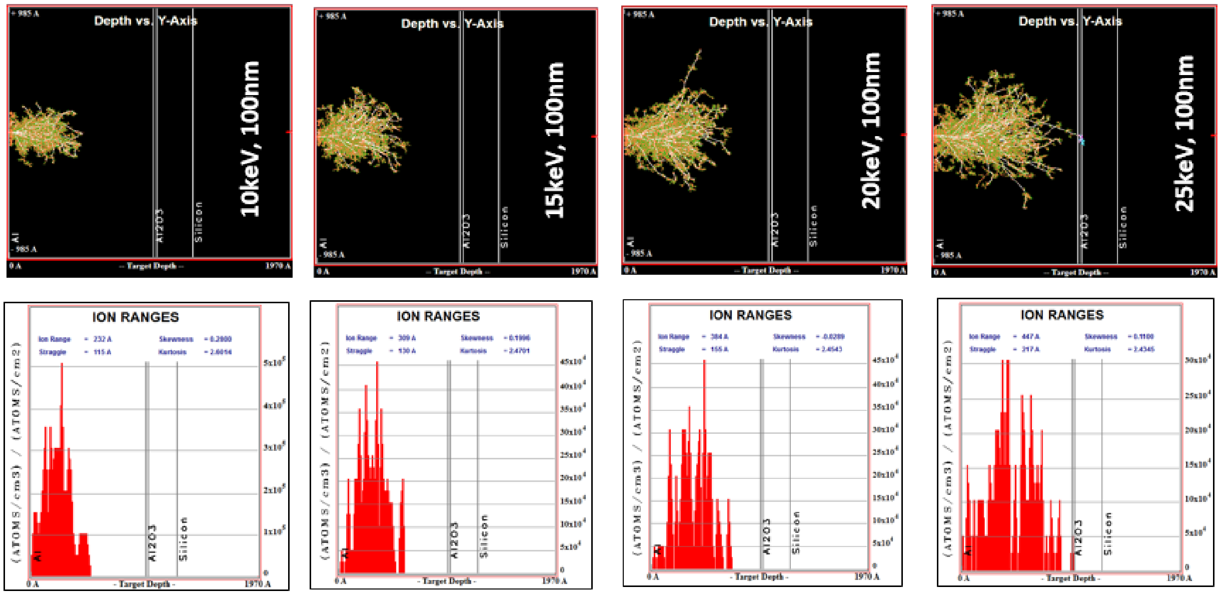

Figure S5 Results of modeling the penetration of Ne^+ neon ions with an energy of 10-25 keV into a Josephson junction with a top electrode thickness of 100 nm

Section 2: Molecular dynamics simulations

Before performing a full-scale calculation, it is necessary to verify the calculations using the molecular dynamics method of collision cascades. ReaxFF potential for the Al-O system (43), proposed for describing chemical reactions, has not been tested for calculating collision cascades. Therefore, comparison with the results of calculations with other potentials is an important issue. Since neon energies reach 20-30 keV, it is necessary to use the ZBL (32) potential to describe the interaction at short distances between atoms (<1.5 Å). Similar potential is used in the SRIM program for calculating binary collisions, so the results should be close. But after the stage of strong collisions, the stage of low-energy interactions begins. For this stage, a large part is played by the potential of interatomic interaction, used for large distances. For problems of forming displacement cascades, potentials of the embedded atom model (EAM) type are usually used, which give values close to the number of defects formed.

Figure S6 shows the results of a calculation of the interaction of an aluminum ion traveling at a speed of 2.17 A/fs (energy 11 keV) in an 8×8×40 nm system. For a given energy, the average depth of defect formation according to SRIM calculations is 10-15 nm, the average number of defects is 186 vacancies/ion.

From sequential frames of ion motion, it is clear that when secondary high-energy ions appear or at the end of the track of the primary ion, a dense cascade of displacements is formed (a lot of atoms are involved). As a result, local amorphization of this area occurs. There can be several such regions for one ion. But further annealing at a temperature of 300 K for 100 ps leads to crystallization of this region with the formation of a complex of defects. The characteristic region of defect formation lies from 5 to 20 nm and the characteristic number of defects is 100-200, which is in good agreement with the results of the SRIM calculation. It is worth noting that for the possibility of crystallization of an amorphous region, the key is the size of the calculation cell and the size of the region. When a continuous region is formed, crystallization may not occur.

Let us compare the results of calculations using 4 potentials for two moments of time - after stopping the primary ion and annealing over the next 100 ps (Fig. S7-8).

It is important to note that the resulting defects (especially interstitial clusters), when calculated with all potentials, are mobile even at a temperature of 300 K on a scale of 100 ps. Therefore, it is expected that in times of 1 μm they completely cover distances of tens of nanometers, reaching either the upper boundary of the layer or the interface with the oxide. Thus, the potential ReaxFF quite well describes the cascade of collisions and their annealing.

The calculation results shown in the figures depend on the position of the ion and the direction of its movement, since the scattering process occurs differently in this case. Figures S9-10 show the calculation results for several launches with similar speeds, but slightly different in direction. It can be seen from the figures that the depth of ion penetration and, accordingly, the depth of defect formation differ in different calculations. It is worth noting that the half-width of the defect distribution in the SRIM calculation is 25 nm, and ranges from 5 to 30 nm. Therefore, the MD results are in good agreement with the SRIM calculations.

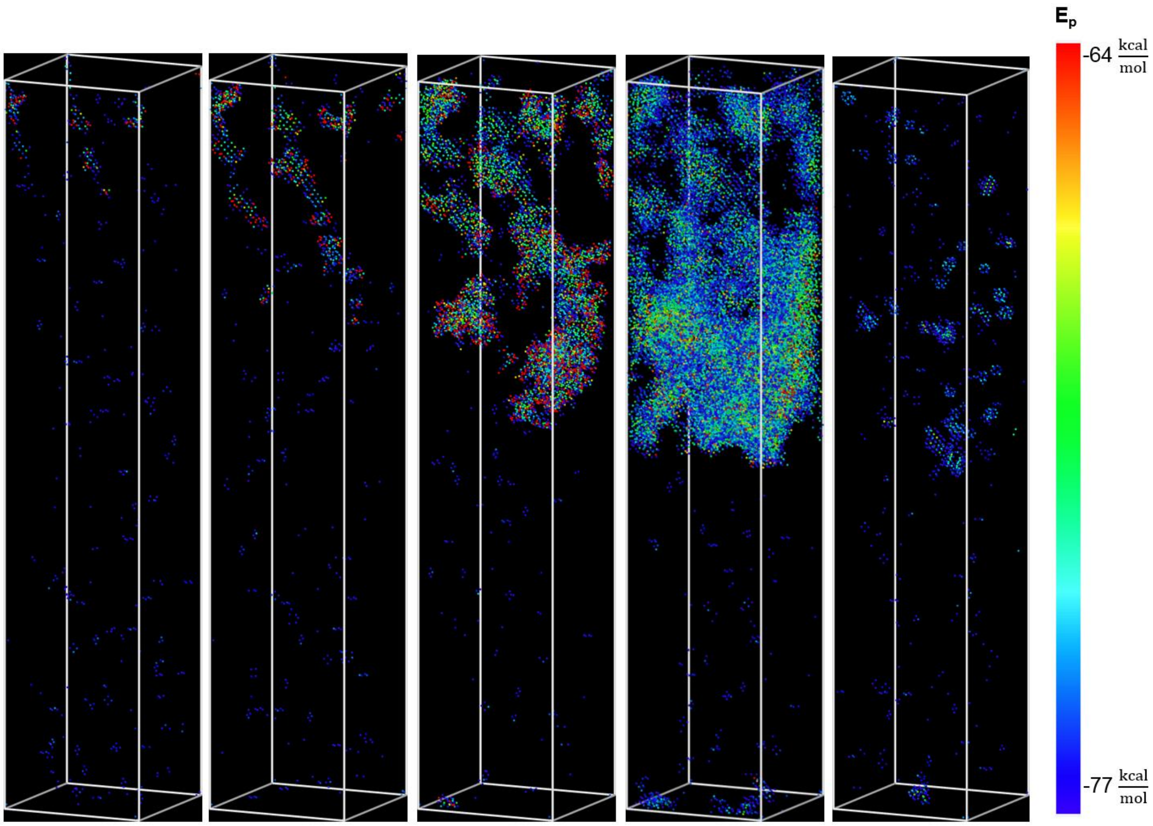

Figure S6 Sequential frames of the structure of a displacement cascade and its annealing with an eam/alloy (44) potential to describe the AlO_x system. Only atoms with a defect in the local environment are shown. Color corresponds to potential energy

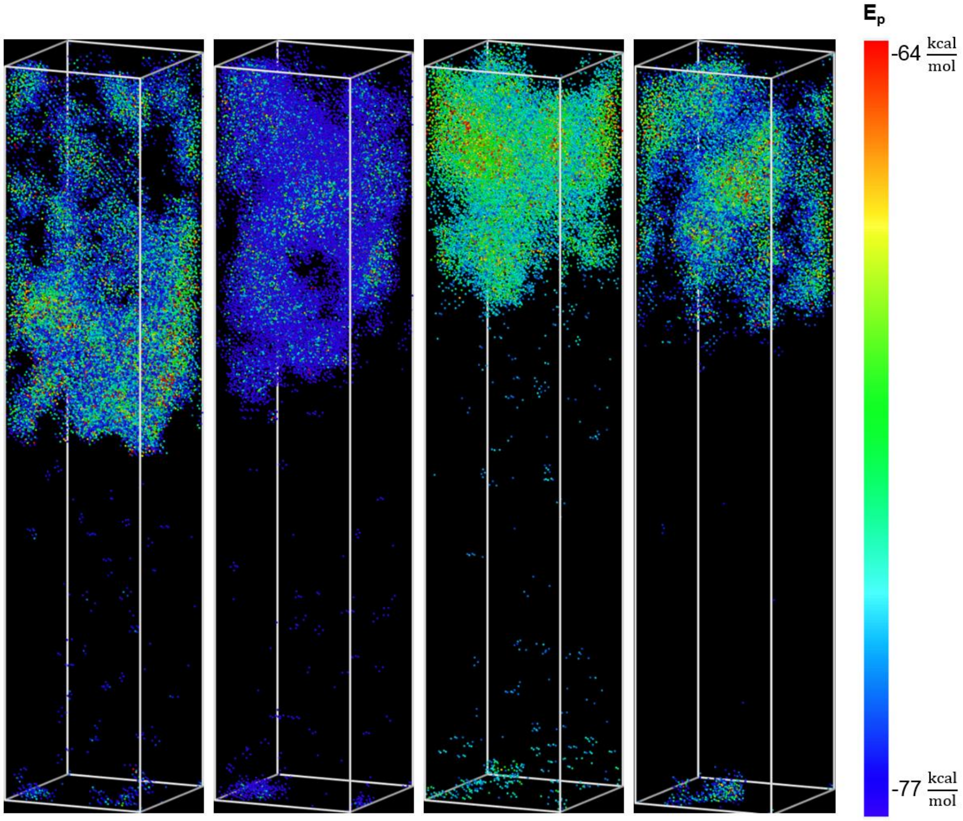

Figure S7 Structure of a displacement cascade with potentials of the eam/alloy (44) type to describe the AlO_x system, eam/alloy (45), eam/alloy (46), ReaxFF (43). Only atoms with a defect in the local environment are shown. The color corresponds to potential energy.

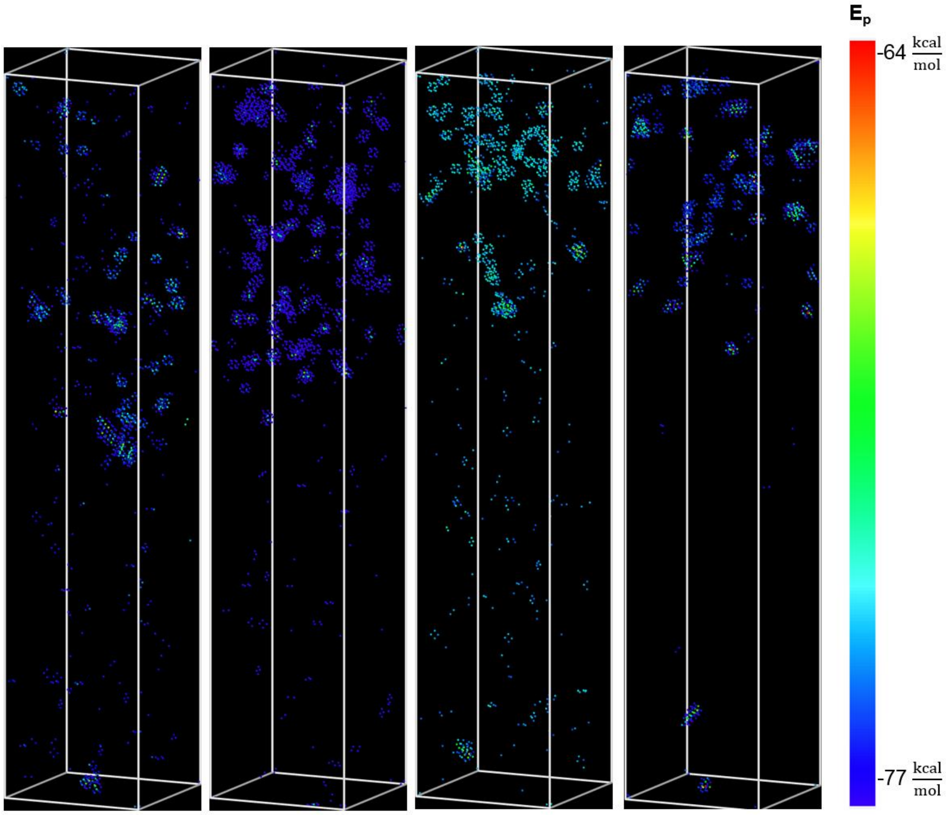

Figure S8 Annealing structure of a displacement cascade with eam/alloy (44) type to describe the AlO_x system, eam/alloy (45), eam/alloy (46), ReaxFF (43). Only atoms with a defect in the local environment are shown. Color corresponds to potential energy.

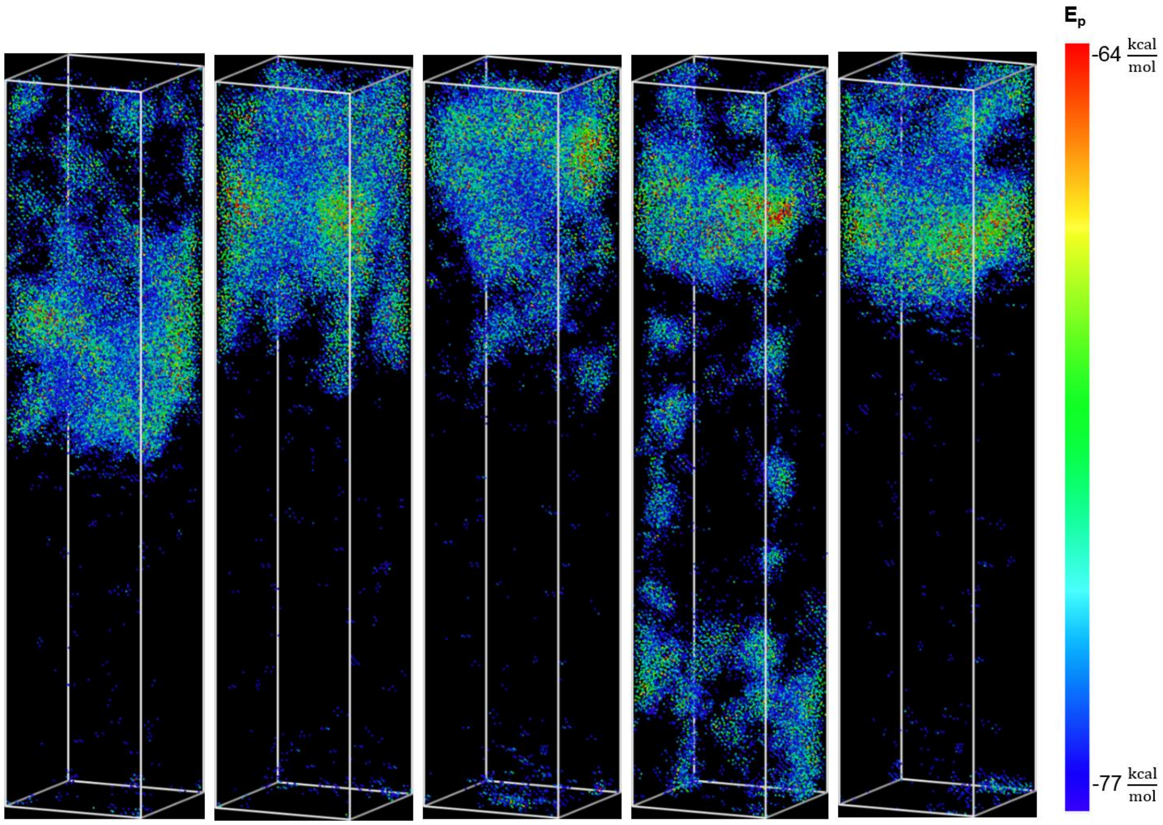

Figure S9 Aluminum eam/alloy type potential bias cascade structure under different launches after amorphization. Only atoms with a defect in the local environment are shown.

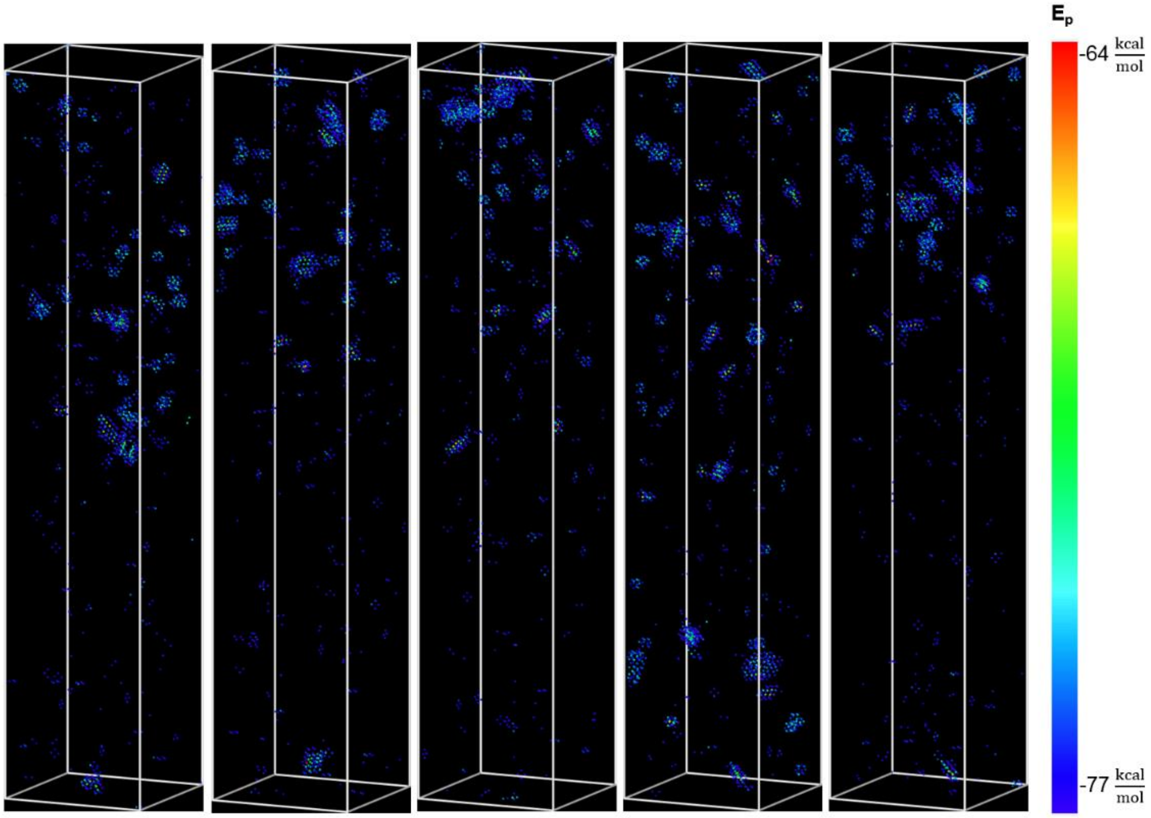

Figure S10 Aluminum eam/alloy type potential bias cascade structure under different launches after aging. Only atoms with a defect in the local environment are shown.

Section 3: Supplementary information on experimental data

The study revealed that the presented post-treatment method does not worsen the initial resistance variation when a single dose is applied to an array of elements. The plot with coefficients of variation before and after treatment for different doses is presented in Figure S11(a).

Experiments comparing Helium and Neon treatments showed that the spread of ΔR values during Helium treatment is on average 1.2 times lower than during neon treatment (Fig. S11(b)). We assume that the reason for this is the mass and, accordingly, the depth of penetration of helium ions into JJ.

Figure S12(a) shows the results of the experiment to reduce resistance variation for Josephson junctions with an area of 150 × 670 nm^2 in the form of normal resistance heat maps. Figure S12(b) shows the histogram of the probability density distribution for JJ with 150 × 670 nm^2 area.

Figure 2(b,c) of the main text shows plots of normal resistance versus treatment dose. Tables S1-5 present the measurement data from these plots.

Equations S1-3 show that the dependence of the normal resistance on the irradiation dose for JJ with a top electrode thickness of 35 nm, 45 nm and 60 nm is linear, the straight lines differ in slope coefficients.

$$\frac{R_{35,a}}{R_{35,b}} = 0.0628 \cdot dose + 1.0559, \quad \text{(S1)}$$

$$\frac{R_{45,a}}{R_{45,b}} = 0.0527 \cdot dose + 1.0268, \quad \text{(S2)}$$

$$\frac{R_{60,a}}{R_{60,b}} = 0.0366 \cdot dose + 1.0045, \quad \text{(S3)}$$

where R_a – value of the normal resistance of the Josephson junction after irradiation, Ohm;

R_b – value of the normal resistance of the Josephson junction before irradiation, Ohm;

$dose$ – irradiation dose, $\times 10^{13} \frac{Ne}{cm^2}$.

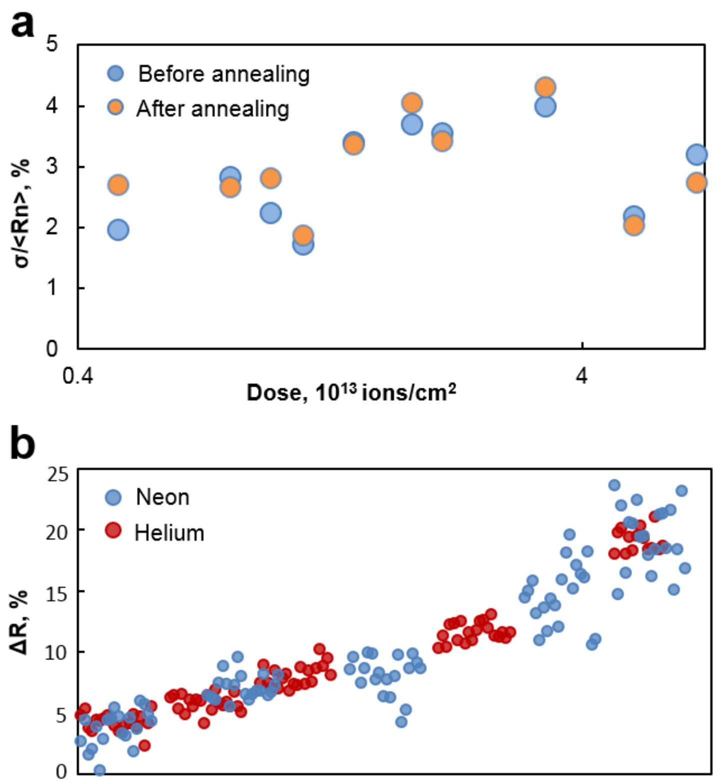

Figure S11: Experimental results of ion beam irradiation of JJ. a Coefficients of variation of normal resistance before and after treatment with different doses. b Comparison of the scatter of values between helium and neon treatments.

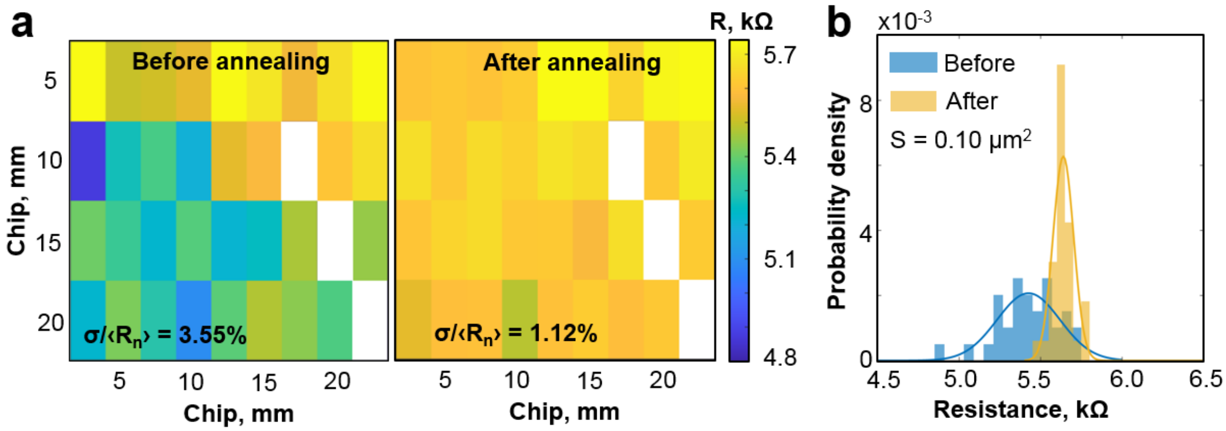

Figure S12: Improving of the chip-scale variation coefficient of room-temperature resistance. **a** Heat maps showing the decrease in the variation coefficient of room resistance values after focused ion beam treatment. The chip size is 25×25 mm^2 and the area of the JJ is 0.10 μm^2. **b** Probability density histograms of the normal resistance distribution before and after ion beam treatment for 0.10 μm^2 Josephson junctions.

Table S1: measurement data of parameters of JJ with an area of 0.04 μm^2 treated with Ne^+

Dose, $\times 10^{13} \frac{Ne}{cm^2}$	Number of structures	R_{nb}, кΩ	σ_b, Ω	σ_b/⟨ R_{nb}⟩, %	R_{na}, кΩ	σ_a, Ω	σ_a/⟨R_{na}⟩, %	ΔR, %
0.0215	20	23.83	886	11.1	24.20	976	12.1	1.6
0.0488	20	23.88	846	10.6	24.34	839	10.3	1.9
0.0873	20	23.61	919	11.6	24.12	930	11.6	2.2
0.179	20	22.79	598	7.9	23.99	643	8.0	5.3
0.323	20	22.13	533	7.2	23.31	540	6.9	5.3
0.716	20	22.56	599	7.9	24.97	621	7.5	10.7
1.58	19	22.92	796	10.4	26.42	994	11.3	15.2
2.96	20	23.52	769	9.8	27.60	1002	10.9	17.4
3.95	19	22.75	739	9.7	30.26	941	9.3	32.9
5.93	20	23.85	769	9.8	30.71	1093	10.7	28.8
7.9	20	22.39	625	8.4	34.08	1135	10.0	52.2

Table S2: measurement data of parameters of JJ with an area of 0.13 μm^2 treated with Ne^+

Dose, $\times 10^{13}\ \frac{Ne}{cm^2}$	Number of structures	R_{nb}, κΩ	σ_b, Ω	$\sigma_b/\langle R_{nb}\rangle$, %	R_{na}, κΩ	σ_a, Ω	$\sigma_a/\langle R_{na}\rangle$, %	ΔR, %
0.018	20	5.87	176	9.0	6.05	195	9.7	2.9
0.084	18	6.18	120	5.8	6.38	98	4.6	3.4
0.189	20	6.24	133	6.4	6.42	117	5.5	2.9
0.378	20	6.20	147	7.13	6.43	94	4.4	3.7
0.481	20	6.27	178	8.5	6.57	175	8.0	4.9
0.755	20	6.18	102	4.9	6.62	134	6.1	7.2
0.802	18	6.19	139	6.7	6.60	185	8.4	6.5
0.963	20	6.16	106	5.2	6.59	124	5.6	7.1
1.06	16	6.33	216	10.2	6.89	232	10.1	8.8
1.12	20	6.11	110	5.4	6.64	226	10.2	8.6
1.41	20	6.23	221	10.6	6.83	233	10.3	9.60
1.51	20	6.13	167	8.2	6.38	153	7.17	4.2
1.84	20	6.24	249	11.9	7.45	321	12.9	19.4
2.11	20	5.97	192	9.6	6.84	255	11.2	14.6
3.38	20	5.93	129	6.6	7.21	146	6.1	21.7
5.07	20	5.88	188	9.6	7.59	208	8.2	29.2
6.76	20	5.87	167	8.5	8.04	274	10.2	36.7

Table S3: measurement data of parameters of JJ with an area of 0.13 μm^2 treated with He^+

Dose, $\times 10^{13}$ $\frac{Ne}{cm^2}$	Number of structures	R_{nb}, кΩ	σ_b, Ω	$\sigma_b/\langle R_{nb}\rangle$, %	R_{na}, кΩ	σ_a, Ω	$\sigma_a/\langle R_{na}\rangle$, %	ΔR, %
0.022	20	6.05	101	5.0	6.21	75	3.6	2.7
0.049	20	6.04	117	5.8	6.17	110	5.3	2.2
0.087	20	6.01	130	6.5	6.14	134	6.5	2.3
0.198	20	6.02	138	6.9	6.22	126	6.1	3.4
0.395	17	5.96	183	9.2	6.14	119	5.8	3.0
0.481	18	5.95	176	8.9	6.06	191	9.4	2.3
0.676	20	6.05	113	5.6	6.26	122	5.9	3.5
0.753	20	6.02	83	4.2	6.27	83	3.9	4.2
0.963	20	5.97	199	10.0	6.20	219	10.6	4.0
1.12	18	5.95	184	9.3	6.16	182	8.8	3.6
1.2	20	6.01	173	8.6	6.36	167	7.9	5.8
2.41	20	5.94	76	3.8	6.42	99	4.6	8.1
3.21	20	5.89	111	5.7	6.57	135	6.2	11.6
4.52	16	6.15	327	16.0	7.21	170	7.1	19.0
6.02	17	6.62	485	22.0	7.66	217	8.5	20.0

Table S4: measurement data of the parameters of JJ with an area of 0.13 μm^2 and a thickness of the top electrode of 35 nm, treated with Ne^+

Dose, ×10^{13} $\frac{Ne}{cm^2}$	Number of structures	R_{nb}, кΩ	σ_b, Ω	$\sigma_b/\langle R_{nb}\rangle$, %	R_{na}, кΩ	σ_a, Ω	$\sigma_a/\langle R_{na}\rangle$, %	ΔR, %
0.179	19	5.41	199	3.7	5.76	209	3.6	6.4
0.358	18	5.28	342	6.5	5.70	326	5.7	8.0
0.537	16	5.10	288	5.6	5.60	300	5.4	9.5
0.716	19	4.96	397	8.0	5.52	483	8.8	11.2
0.846	20	5.50	558	10.2	6.04	560	9.3	10.0
1.01	18	5.59	567	10.1	6.21	602	9.7	10.7
1.18	20	5.78	558	9.7	6.51	620	9.5	12.6
1.35	19	5.42	139	2.6	6.23	134	2.2	15.1

Table S5: measurement data of the parameters of JJ with an area of 0.13 μm^2 and a thickness of the top electrode of 60 nm, treated with Ne^+

Dose, ×10^{13} $\frac{Ne}{cm^2}$	Number of structures	R_{nb}, kΩ	σ_b, Ω	$\sigma_b/\langle R_{nb}\rangle$, %	R_{nb}, kΩ	σ_b, Ω	$\sigma_b/\langle R_{nb}\rangle$, %	ΔR, %
0.198	18	6.74	245	3.6	6.83	236	3.5	1.3
0.312	18	6.88	151	2.2	6.97	160	2.3	1.4
0.878	19	6.87	192	2.8	7.08	242	3.4	3.1
1.09	20	6.87	163	2.4	7.14	274	3.8	4.0
1.36	16	6.83	158	2.3	7.29	156	2.1	6.6
1.64	16	6.92	194	2.8	7.38	181	2.4	7.1
1.91	17	6.93	145	2.1	7.42	96	1.29	7.2
2.18	16	6.81	287	4.2	7.40	216	2.9	7.9

Section 4: Alternative hypotheses for the mechanisms of ion treatment on buried oxides

The effect of ballistic mixing on oxide amorphization

One of the criteria that was considered as a factor influencing the oxide layer was the ballistic mixing mechanism. Ballistic mixing occurs due to binary collisions of high-energy ions with lattice atoms or ions. As a result, atoms are mixed from the position of the crystal lattice. The greatest effect is observed at the end of the track, when all the remaining energy appears in small quantities. In metallic materials, when irradiated enough to be destroyed by ions, zones with local melting occur, which partially crystallize during annealing. In ionic crystals, covalent or intermetallic compounds, the formation of an amorphous state is possible.

In the case of irradiation with neon ions, most of the energy is transferred to aluminum atoms in the upper aluminum layer, which in turn knock out aluminum and oxygen ions in the oxide layer. For 15 keV neon with a thickness of 45 nm, the results are shown in Figure S13.

The number of contacts in the oxide layer is 0.2 per ion per Å. The energy loss for this furnace is approximately 10 eV/Å. Thus, the collision with the source averages approximately 50 eV. Thus, the average energy of knocked out ions in the oxide is determined. To perform calculations, it is necessary to determine the number of ions that are knocked out in the simulated distribution. The area of the calculation cell in MD modeling is $11\, nm^2 = 11 \cdot 10^{-14}\, cm^2$. With a dose of 10^{13} ions, 1 ion falls on average onto the calculated area. The thickness of the calculation cell is 26 Å. Thus, the number of collisions is $0.2 \cdot 26\ =\ 5$.

Thus, for a neon energy of 15 keV to simulate ballistic mixing, it is necessary to launch 5 ions with an average energy of 50 eV in the calculation cell. An energy of 20 keV will correspond to approximately 10 ions.

In the MD calculation, the ions were fired sequentially to simulate annealing after each ion, since thermalization occurs quickly. Thermalization was carried out at temperatures of 300, 450 and 600 K for 50 ps after each ion. Different temperatures were chosen to speed up the process, since in

the experiment the characteristic irradiation times are microseconds, and the experimental time is seconds, hours. The ion and direction were chosen randomly. 5 initial structures were considered (Figure S14) - from a completely amorphous system to a completely crystalline one. In the MD calculation, the ions were fired sequentially to simulate annealing after each ion, since thermalization occurs quickly. The ion and direction were chosen randomly.

Each launched ion creates a local amorphous region that partially crystallizes. But in general there is an increase in the amorphous region. When the 9th ion was knocked out, almost half of the crystalline oxide became amorphized.

Thus, it has been shown that ballistic mixing with ion energies and doses characteristic of the oxide layer when irradiated with neon ions leads to amorphization of the oxide layer.

We attribute the increase of resistance to a local structure change of the oxide rather than stoichiometry. To summarize, there is no change in stoichiometry in the experimentally studied modes for processing qubits. The thickness of the oxide also does not change significantly, which means that there should be no change in hysteresis or breakdown voltage. The results of modeling the change in the coordination number during ion irradiation are presented in Figure S16.

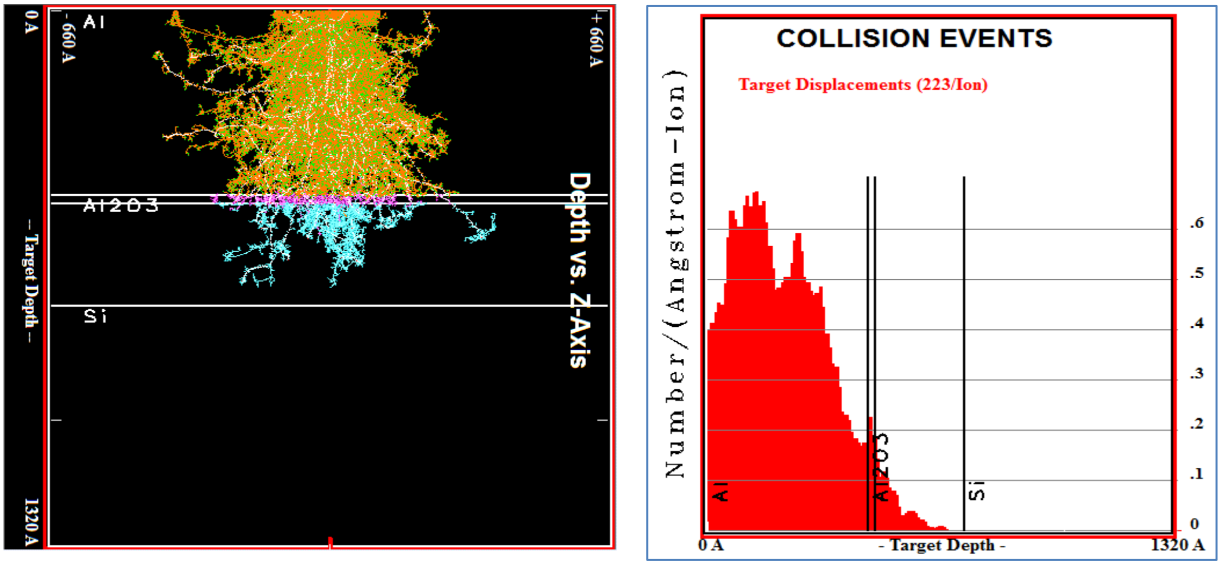

Figure S13 Calculation of SRIM distribution of binary collision events

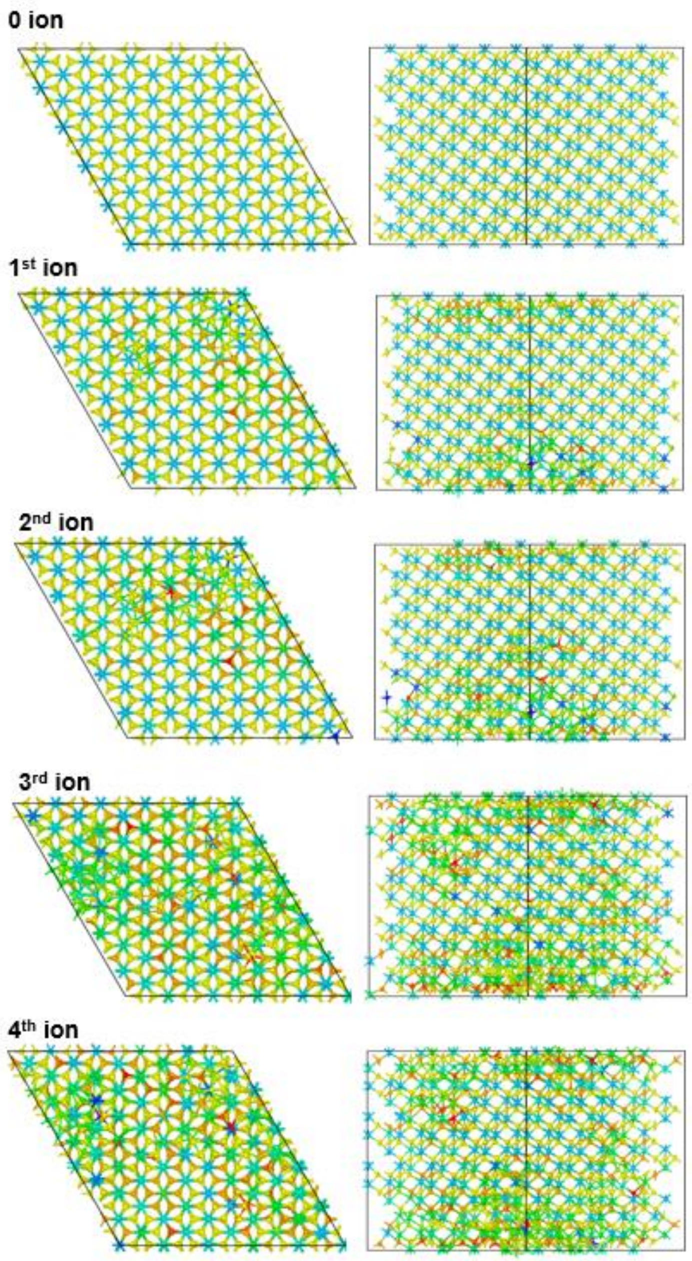

Figure S14 Oxide structure (top and side views) with different numbers of launched ions: 0-4

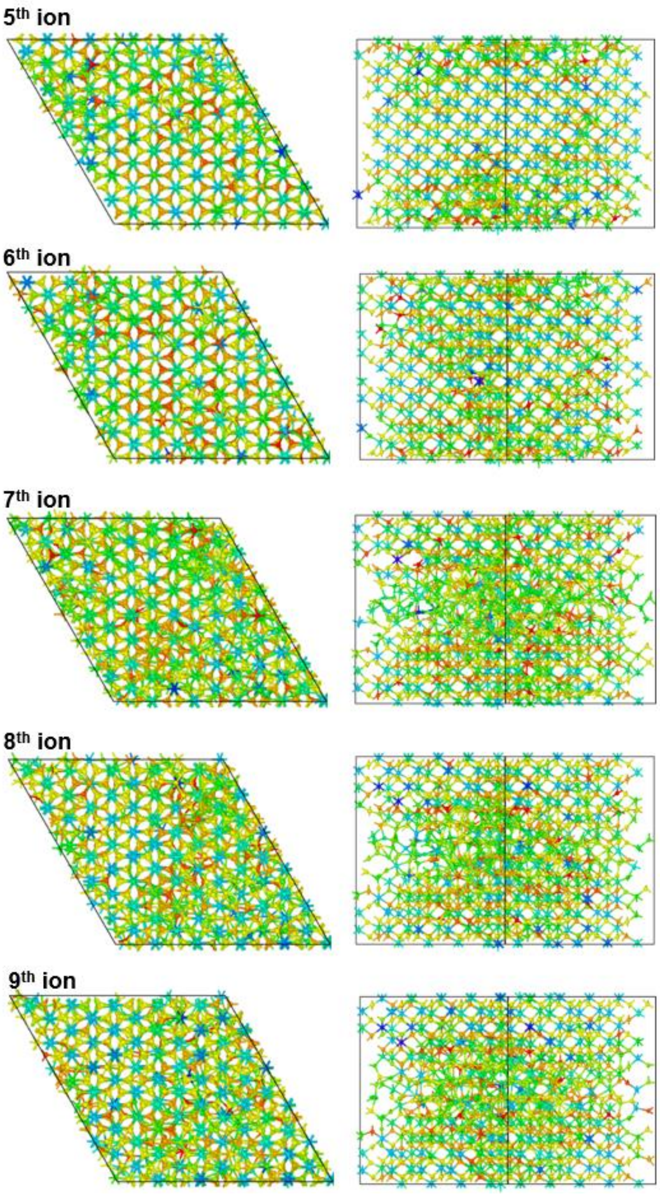

Figure S15 Oxide structure (top and side views) with different numbers of launched ions: 5-9

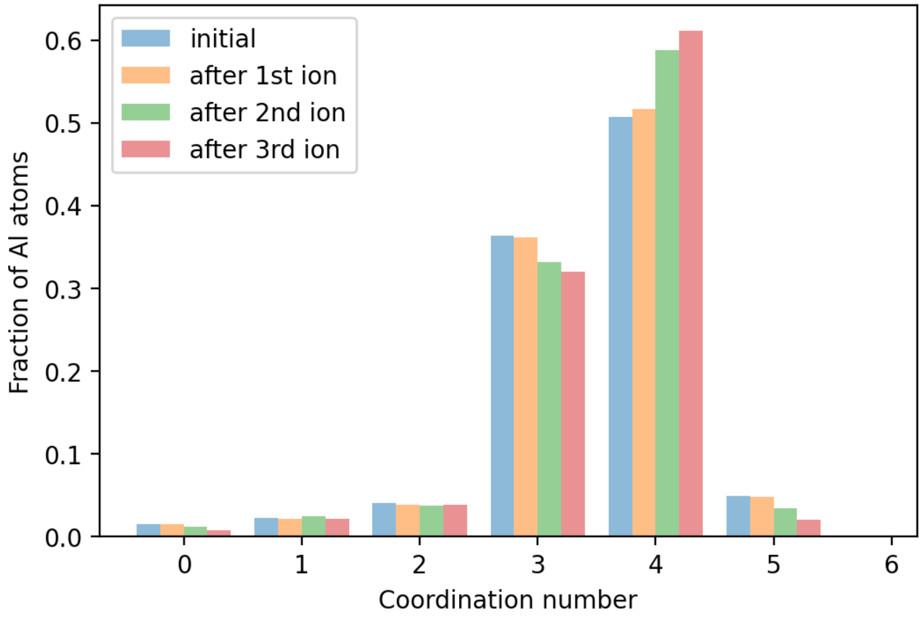

Figure S16 The results of modeling the change in the coordination number during ion irradiation: intial - before ion irradiation; after 1st ion - the first ion does not reach the barrier; after 2nd ion - the second ion penetrates and amorphizes the oxide; after 3rd ion - the third ion passes through the oxide and also amorphizes it

Heating mechanism

We also considered the heating mechanism as a result of elastic and inelastic collisions of the ion with the sample. Several effects are observed as a result of elastic collisions, including sputtering of neutral and ionized atoms of the material, emission of electrons, displacement of lattice atoms in the processed material, and heating of the material associated with the emission of phonons. Inelastic collisions cause electronic excitation and relaxation, which also leads to heating of the sample[2]. The rate of energy loss of an ion can be expressed as:

$$\left.\frac{dE}{dx}\right|_{tot} = \left.\frac{dE}{dx}\right|_{Nu} + \left.\frac{dE}{dx}\right|_{el}, \qquad \text{(S4)}$$

where $\left.\frac{dE}{dx}\right|_{Nu}$ - rate of energy loss during elastic nuclear collisions, $\left.\frac{dE}{dx}\right|_{el}$ - c rate of energy loss during electron collisions. It is known from the literature[3] that for ions with low energy E<100 keV, energy losses due to nuclear braking predominate.

The ZBL function is used to estimate energy loss during nuclear collisions[4]. For practical calculations, the universal nuclear stopping power ZBL for an ion with energy E is given by Eq. 5.

$$S_n(E) = 8.462 \times 10^{-15} \frac{Z_1 Z_2 M_1 S_n(\varepsilon)}{(M_1 + M_2)(Z_1^{0.23} + Z_2^{0.23})} \left[\frac{eV \times cm^2}{atom}\right], \qquad \text{(S5)}$$

where Z_1, Z_2 – atomic numbers of beam ions and Josephson junction material, respectively, M_1, M_2– mass of ion and material JJ in a.m.u, $S_n(\varepsilon)$ – reduced nuclear stopping cross-section.

At low energies E, the stopping cross section of an ion is proportional to its velocity. The model of electronic braking in a mode proportional to speed is due to Lindhard and Scharff (47). The stopping cross-section can be expressed as:

$$S_e(E) = 3.83 \times 10^{-15} \frac{Z_1^{\frac{7}{6}} Z_2}{\left(Z_1^{\frac{2}{3}} + Z_1^{\frac{2}{3}}\right)^{\frac{3}{2}}} \times \left(\frac{E}{M_1}\right)^{\frac{1}{2}} \left[\frac{eV \times cm^2}{atom}\right], \quad \text{(S6)}$$

With ion energy $E_{Ne} = 10\ keV$, the rate of energy loss due to electron collisions, taking into account the atomic density of aluminum, is equal to $\left.\frac{dE}{dx}\right|_e = S_e\mathrm{N} = 95.7\,\frac{\mathrm{eV}}{\mathrm{nm}}$, and for nuclear collisions $\left.\frac{dE}{dx}\right|_{Nu} = S_n\mathrm{N} = 284.2\,\frac{eV}{nm}$.

To verify the analytical calculation and evaluate the effects that arise in the volume of the Josephson junction under the action of a focused ion beam, Monte Carlo simulation was performed in the SRIM environment[5]. The study estimated the influence of neon and helium ions on the aluminum crystal lattice, as well as the JJ tunnel barrier at a given beam energy. Modeling shows that in the case of working with neon, the peak concentration of ions of the primary beam is inhibited already at a depth of 20 nm from the surface of the upper electrode JJ, and only 2% of the ions of the primary beam reach the tunnel barrier. From the literature[6], the interaction of a helium beam with a silicon substrate is known, leading to the formation of bubbles in the volume of the material. Based on these data, further modeling was carried out for a beam of neon ions. As shown in Figure 3b, ionization processes in combination with the formation of phonons under the influence of recoiling atoms lead to the fact that 96% of the energy of the primary beam goes to heating the Josephson junction. The amount of heat transferred by the ion beam to the Al/Al-O_x/Al atoms of the Josephson junction was used as an input parameter in the finite element simulation in COMSOL software. We simulated the collision of a single neon ion (Ne^+) with an energy of 10 keV with a 3D Josephson junction model. The time-dependent differential heat conduction equation for a stationary, homogeneous, isotropic solid was used as the design equation:

$$\rho c_p \frac{\partial T(\vec{r},t)}{\partial t} = \nabla \times [k \nabla T(\vec{r},t)] + g(\vec{r},t), \quad \text{(S7)}$$

where ρ – density [$\frac{kg}{m^3}$], c_p - specific heat [$\frac{J}{kg \times K}$], k – thermal conductivity [$\frac{W}{m \times K}$], T – temperature [K], t – time [s], g – heat source [$\frac{W}{m^3}$].

A simulation was performed characterizing the energy transfer (0,96×E_{Ne}) of a single neon ion into the volume $\pi r_{phonon}^2 z$ over time t. Here E_{Ne} = 10 keV – ion energy, r_{phonon} = 3 nm – half-width of phonon heating, z is the depth of ion penetration into the JJ volume. Due to multiple collisions of the ion with atoms of the aluminum crystal lattice, the trajectory of the ion changes, which leads to the fact that the total path length of the ion is determined by the projected path R_p in the direction parallel to the direction of the incident ion, which corresponds to the parameter z.

Figure S17 (b-c) shows the temperature profile of a point at the center of an Al/AlO_x/Al Josephson junction tunnel barrier generated by a 0.0094 W heat source, equivalent to the volume of one ion track, during the time it takes for a neon ion to stop in the sample (170 fs). The stopping time of the ion was calculated based on Lindhard's theory, which states that the energy loss when stopping an ion is proportional to the speed. As a result of the simulation, a curve was constructed to plot the dependence of heating in the center of the tunnel barrier on the depth of ion penetration into the JJ's volume. At the initial sample temperature corresponding to room temperature, the maximum temperature increase in the center of the tunnel barrier of the JJ due to the impact of a single ion is ΔT_{single_ion} = 360°C. We hypothesize that rapid local heating of the JJ leads to the formation of new AlO_x bonds and, as a consequence, an increase in the thickness of the tunnel barrier, thereby increasing R_n.

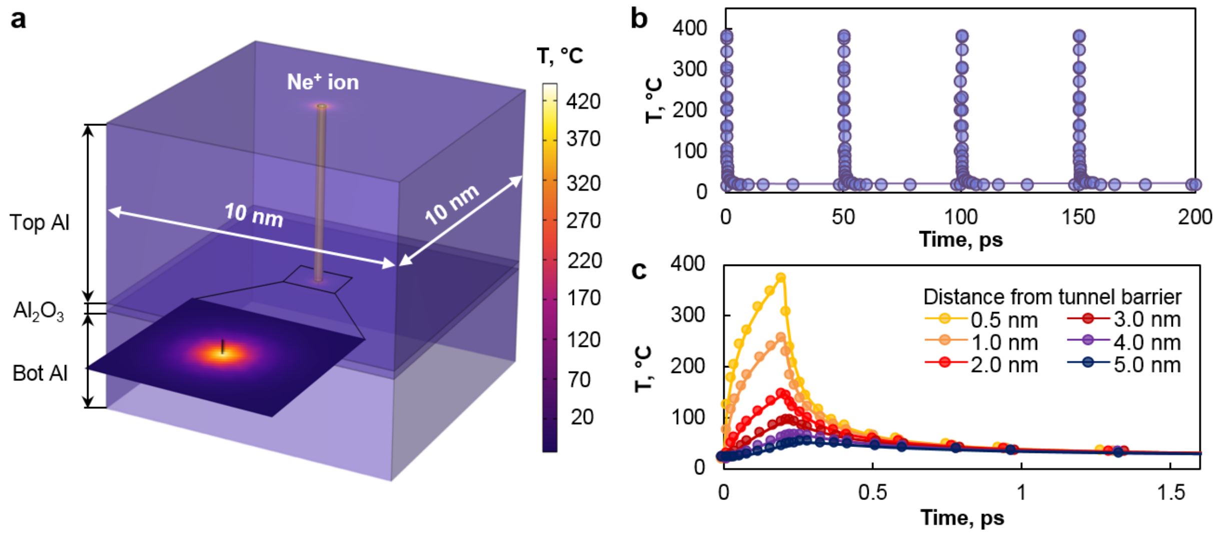

Figure S17: Results of finite element simulations. a Model of thermal heating of the Josephson junction structure by a focused ion beam in the COMSOL environment. We simulated the collision of a single neon ion (Ne+) with an energy of 10 keV with a 3D Josephson junction model with functional layer thicknesses of 25/2/45 nm, respectively. The time-dependent differential heat equation for a stationary, homogeneous, isotropic solid was used as the design equation. **b-c** Temperature profile of a point at the center of the tunnel barrier of an $Al/AlO_x/Al$ Josephson junction, created by a heat source with a power of 0.0094 W, which is equivalent to the volume of one ion track, during the time required for a neon ion to stop in the sample (170 fs). At the initial sample temperature corresponding to room temperature, the maximum temperature increase in the center of the tunnel barrier of the DP due to the impact of a single ion is $\Delta T_{single\_ion} = 360°C$.

Section 5: Supplementary information on the TEM measurements

As described in the main text of the paper, we made TEM measurements of Josephson junctions before and after ion beam irradiation. For the study, chips with a JJ's with a nominal area S = 0.04 μm^2 and normal resistance Rn were fabricated. Transmission electron microscope (TEM) samples were prepared by a focused ion beam instrument with a gas injection system (Helios, Thermo Fisher Scientific Ltd.). The TEM samples were thinned to electron beam transparency by a Ga+ ion beam from 30 to 2 kV. The TEM samples were investigated by an aberration-corrected TEM (Titan Cube, Thermo Fisher Scientific Ltd.) at 200 kV. A high-angle annular dark-field (HAADF) detector was used for dark-field imaging in scanning TEM (STEM) mode with a convergent semi-angle and a collection semi-angle of 18 m rad and 74-200 m rad, respectively. Energy-dispersive X-ray spectroscopy (EDS) studies were carried out with probe currents of 250 nA.

Figure S18 shows HIM-image of JJ and high-resolution TEM images of JJ before and after ion beam annealing with dose 8×10^{13} Ne^+/cm^2.

Figure S19 shows the TEM and STEM images of JJ and the intensity profiles. As can be seen in the figure, treatment with an ion beam does not introduce visible changes or destruction to the tunnel barrier.

The thickness of the tunnel barrier was different on the two samples, with the sample to which the treatment was applied being thinner. However, we suppose this is due to the variation in the thickness of the tunnel barrier over the junction area, and not due to the effect of ion treatment. According to the recent research (*50*), an oxide thickness t can vary with the standard deviation σ_t $\approx$ 0.35 nm across a Josephson junction. In our TEM measurements we got the thickness about 1.89 nm and 1.5 nm before and after annealing correspondingly. One can see that the thickness is of the order of the variation σ_t and can change significantly across the junction. Therefore, relying solely on the TEM data one can not make any reliable conclusion on the junction thickness change. There are several factors that may contribute to the barrier thickness variation oxidized directly on the Al bottom layer: (1) The oxide barrier closely follows the morphology of the top surface of the bottom Al layer. However, due to the presence of the grain boundary grooving of bottom layer,

the barrier tends to be thicker at grain boundaries; (2) Since the bottom Al layer is polycrystalline, the Al grains have different crystallographic orientations along the film growth direction. Even on one single grain, the curvature of the top surface of the Al grain indicates that there might be local variation in the crystallographic orientation of Al. When oxidized, it will thus give rise to a variation in oxide thickness; (3) Atomic steps of Al at the Al/AlOx interfaces result in local change of the barrier thickness. We demonstrate all the TEM images to confirm non-destructive manner of ion-beam treatment.

EDX analysis was also performed before and after ion beam treatment (Figure S20). Neon peaks were not found in EDX analysis. Probably due to the low content in the lamella. Despite the high resolution of the TEM-based EDX, the thickness of the tunnel barrier in the intensity spectrum is ≈2 times greater than in the intensity spectrum of the STEM image. When scanning an area, the data is summarized over the entire thickness of the lamella. In different sections of the lamella, the tunnel oxide lies at slightly different heights due to the roughness of the grains of the bottom electrode, which is clearly visible in Figure S20 (d), but the data is summarized over all sections of the lamella.

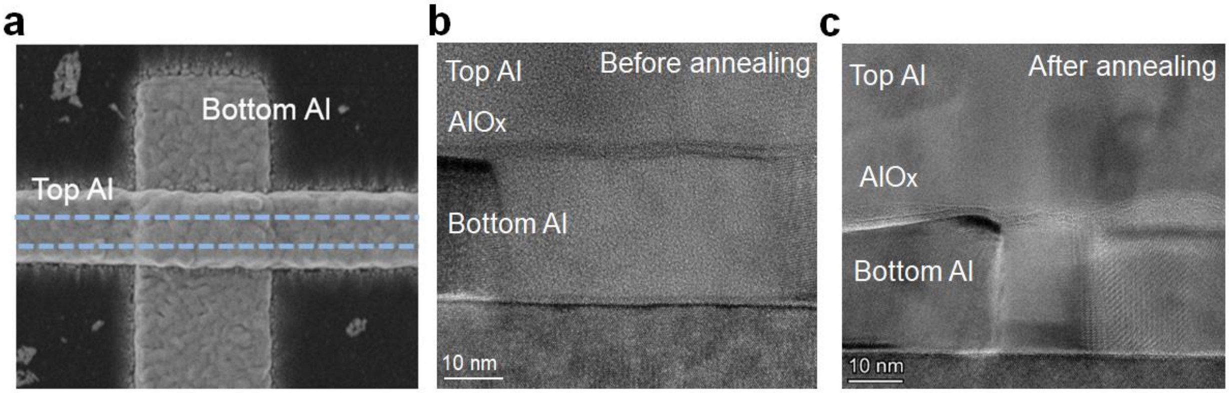

Figure S18: TEM-images of JJ before and after ion irradiation. a HIM-image of JJ with area 0.04 μm^2. The blue dash corresponds to cutting line to acquire the cross-section of the JJ for high resolution transmission electron microscopy (TEM). **b** TEM of the central area of the JJ shown in (a) **c** TEM of the central area of the JJ after Ne^+ ion beam annealing.

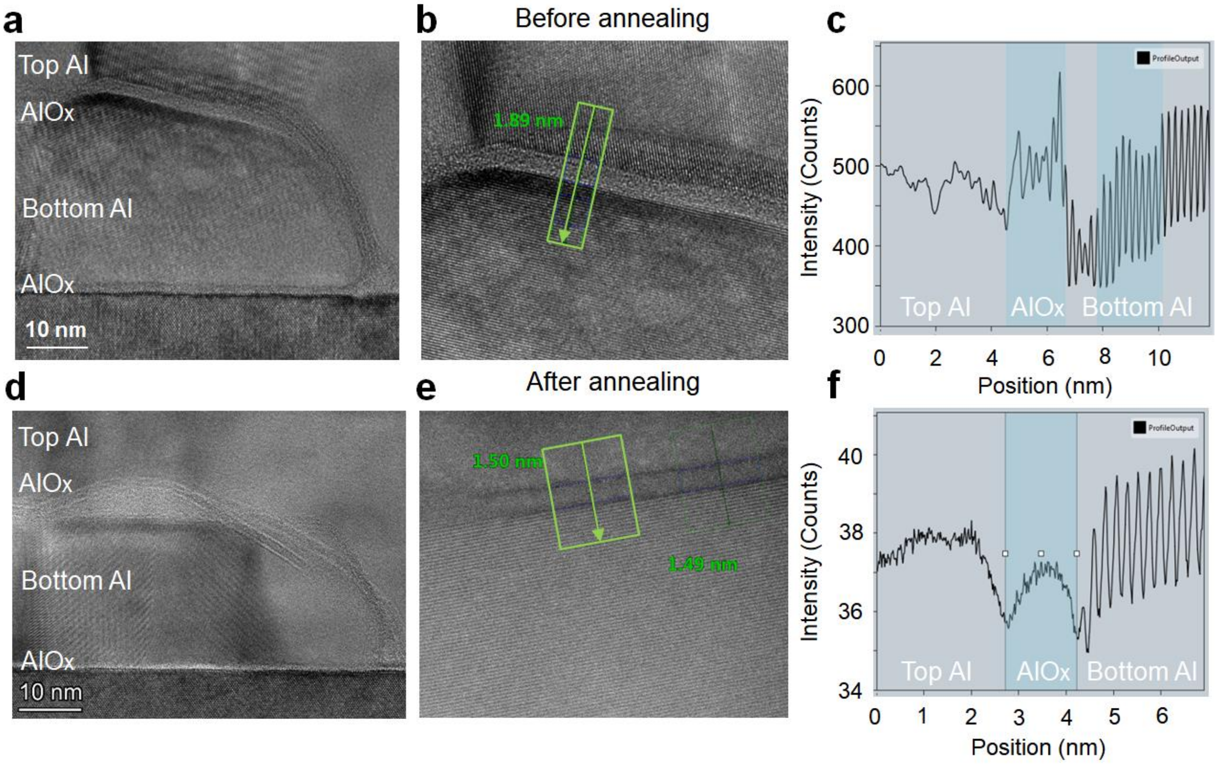

Figure S19: TEM-images of JJ before and after ion irradiation. TEM (**a**) and STEM (**b**) images of JJ's tunnel barrier before ion beam treatment. **c** Intensity profile for the tunnel barrier region before ion beam treatment. TEM (**d**) and STEM (**e**) images of JJ's tunnel barrier after Ne^+ ion beam treatment. **f** Intensity profile for the tunnel barrier region after Ne^+ ion beam treatment.

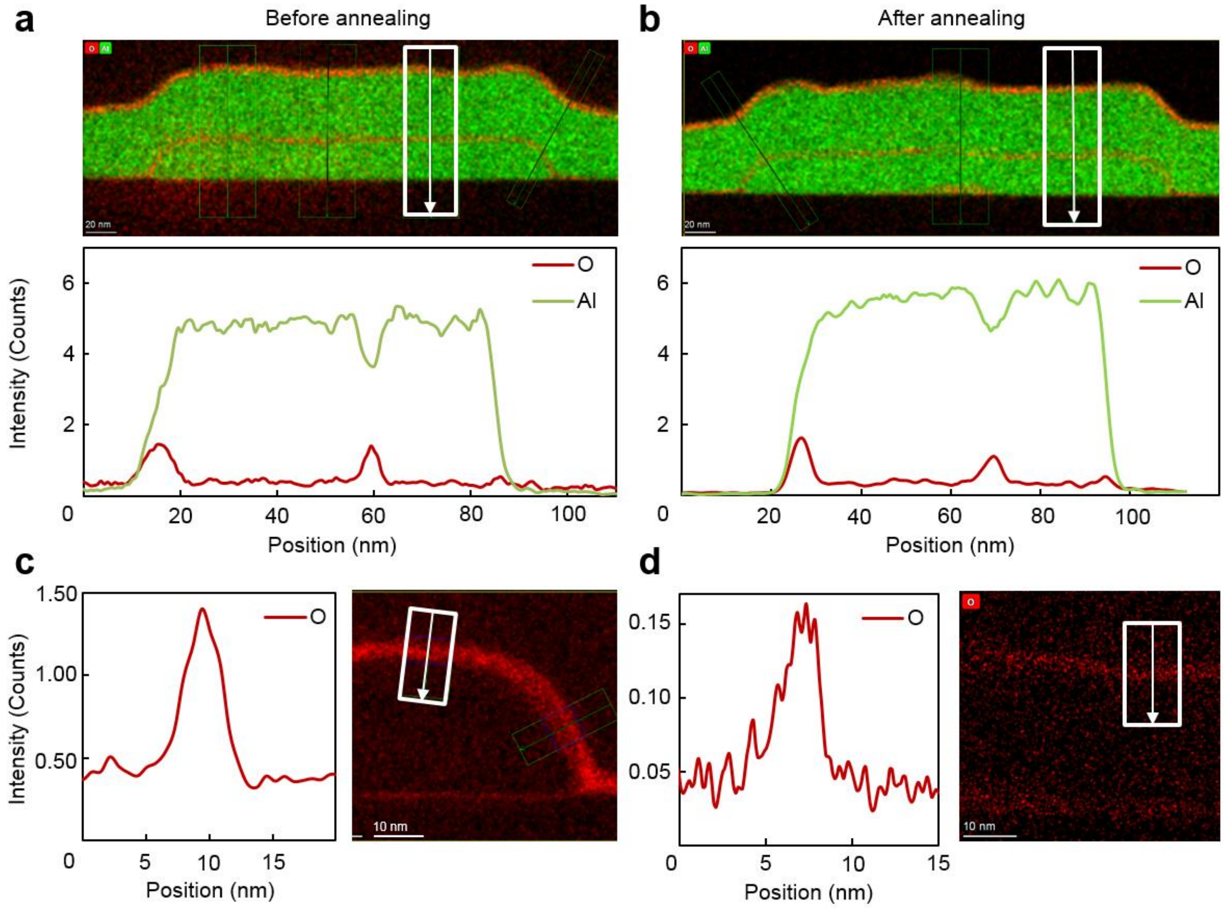

Figure S20: Results of EDX measurements of JJs. a-b EDX map and intensity profiles of the entire Josephson junction before (a) and after (b) ion beam treatment. The figure shows Al (green) and O (red) distributions **c-d** EDX map and oxygen intensity profile of the Josephson junction before and after ion beam treatment.

Section 6: The contribution of the metal (Al layer) to the overall resistance

To quantitatively estimate the resistance variation of aluminum electrodes caused by ion treatment, we conducted additional experiments irradiating the electrodes of Josephson junctions outside their tunnel barriers (Figure S21). We treated two regions of each Josephson junction: bottom Al electrode with oxide on its surface (zone 1) and top Al electrode on the oxide and bottom Al electrode (zone 2). In this experiment we irradiate two different regions of the junction: one aluminum layer in zone 1 and two aluminum layers in zone 2. We chose a deliberately high dose (8×10^{13} ions/cm^2) to increase the possible effect of the irradiation. We irradiated electrodes on 19 identical structures and measured their resistance before and after irradiation.

First, before ion treatment we measured each Josephson junction for 5 times and average normal resistance (R_{aver1}) and sigma (σ, Ohm) of each Josephson junction. Second, after ion treatment we measured them again in the same way. ΔR is a normal resistance change due to ion treatment $\Delta R = R_{aver2} - R_{aver1}$. The table S6 shows the measurement results.

One can see that ΔR caused by ion treatment of Al electrodes is negligible compared to the resistance changes after tunnel barrier irradiation. That is why we concluded that ion irradiation does not cause permanent changes in aluminum structure leading to its significant normal resistance variation even at high doses.

We also calculated the contribution of the aluminum electrodes as follows: we measured the resistance of a structure that fully replicates the electrodes geometry, but does not contain a tunnel barrier. Then we fabricated it with the same process as in the main experiments, but without shadow evaporation step. The experimentally measured room temperature resistance of the electrodes is in the range from 25 to 30 Ohm (this range is due to electrodes geometry variation). From the other hand, the Josephson junction resistance in our case is in the range from 4.96 kOhm to 6.62 kOhm (for large junction areas) and from 22.13 kOhm to 23.88 kOhm (for small areas). Thus, the fraction of electrode resistance in the total resistance of the junctions is from 0.6% to 0.1%.

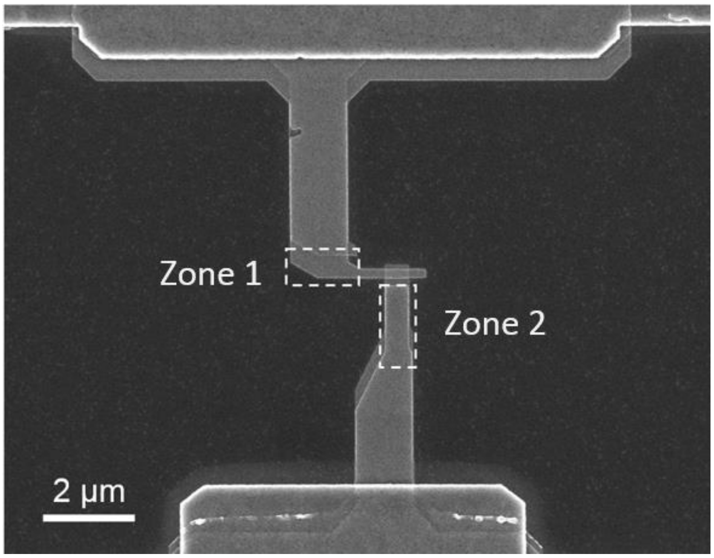

Figure S21 SEM image of Josephson junctions. Irradiated areas are marked with the white rectangles.

Table S6: measurement data of the resistance of JJs with irradiated electrodes

	Before irradiation, Ohm							After irradiation, Ohm							ΔR, Ohm
#	R_1	R_2	R_3	R_4	R_5	R_{aver1}	σ, Ohm	R_1	R_2	R_3	R_4	R_5	R_{aver2}	σ, Ohm	
1	6176	6178	6179	6177	6178	**6178**	**1.0**	6161	6160	6160	6159	6158	**6159**	**1.2**	**-19**
2	6351	6355	6353	6351	6351	**6352**	**1.7**	6331	6333	6333	6335	6333	**6333**	**1.0**	**-19**
3	6431	6431	6429	6429	6430	**6430**	**0.9**	6452	6455	6452	6452	6454	**6453**	**1.6**	**23**
4	6475	6476	6475	6475	6475	**6475**	**0.5**	6466	6469	6466	6465	6468	**6466**	**1.8**	**-9**
5	6378	6377	6374	6376	6377	**6376**	**1.3**	6343	6341	6342	6343	6342	**6341**	**1.0**	**-35**
6	6295	6295	6293	6294	6295	**6295**	**1.0**	6270	6269	6270	6272	6269	**6270**	**1.2**	**-25**
7	6198	6198	6197	6195	6193	**6196**	**2.3**	6171	6175	6174	6175	6177	**6174**	**1.8**	**-22**
8	6149	6150	6149	6150	6153	**6150**	**1.5**	6159	6158	6159	6160	6162	**6159**	**1.4**	**9**
9	6437	6437	6435	6436	6436	**6436**	**1.1**	6406	6409	6408	6409	6409	**6408**	**1.2**	**-28**
10	6338	6336	6338	6340	6339	**6338**	**1.3**	6343	6341	6342	6342	6341	**6341**	**0.8**	**3**
11	6109	6108	6110	6109	6107	**6109**	**1.0**	6105	6110	6110	6112	6112	**6110**	**2.7**	**1**
12	6596	6595	6593	6593	6591	**6594**	**1.9**	6590	6591	6590	6594	6594	**6591**	**1.9**	**-3**
13	6477	6477	6477	6477	6475	**6477**	**0.8**	6488	6490	6490	6488	6487	**6488**	**1.2**	**11**
14	6237	6237	6238	6236	6236	**6237**	**0.9**	6243	6244	6242	6242	6243	**6242**	**0.5**	**5**
15	6336	6336	6335	6334	6334	**6335**	**0.9**	6345	6347	6347	6348	6348	**6347**	**0.8**	**12**
16	6313	6311	6311	6310	6309	**6311**	**1.4**	6320	6322	6322	6322	6322	**6321**	**0.6**	**10**
17	6073	6074	6072	6074	6074	**6073**	**0.9**	6092	6089	6092	6092	6092	**6091**	**1.4**	**18**
18	6163	6162	6161	6161	6160	**6162**	**1.3**	6273	6273	6273	6275	6273	**6273**	**0.7**	**111**
19	6028	6031	6029	6030	6029	**6030**	**0.8**	6048	6046	6047	6049	6049	**6047**	**1.2**	**17**

Section 7: Supplementary information on experiments with qubits

This section provides details on the changes in the characteristics of superconducting quantum circuits as a result of ion beam irradiation. The aim of each experiment was to solve the frequency crowding problem. All data are summarized in Table S6. For additional information on superconducting qubits fabrication see the papers (37, 48, 49).

Section also provides circuit diagrams of the qubit chips in Figures S21-24 and measurement data (T_1, T_2 Echo, T_2 Ramsey) of qubits before and after ion beam irradiation in tables S7-S8.

We carried out repeated measurement of 3 annealed qubits from 7Q chip 14 days after the first cooldown. The qubits were stored at room temperature in a vacuum. The results are shown in the Table S9. The change in the frequency of the annealed qubits due to aging after 14 days ranges from 17 to 26 MHz (0.35%-0.55%). We attribute fluctuations in relaxation time and decoherence to the reconfiguration of TLS frequencies after repeated cooling, since both increases and decreases of T1, T2 are noticeable, as well as on the rest of the annealed qubits.

Table S7: parameters of qubits before and after ion treatment

Description	Qubit number	f_b, GHz	E_c, MHz	E_{jb}, GHz	R_{nb}, κΩ	Dose, $\times 10^{13}\ \frac{Ne}{cm^2}$	f_a, GHz	E_{ja}, GHz	R_{na}, kΩ	T_{1b}, μs	T_{2b}, μs	T_{1a}, μs	T_{2a}, μs	f_r, GHz
1Q	1	4.22	251	9.96	14.8	0.96	4.089	9.62	15.1	373	379	345	303	5.96
	2	4.527	251	11.37	14.8	1.05	4.385	9.62	13.3	159	167	323	217	6.46
2Q	3	5.86	222	20.83	7.2	0.44	5.794	20.37	7.0	25		12	16	6.89
	4	5.958	227	21.06	7.1	0.42	5.896	20.65	6.9	25		27	31	6.93
	5	5.799	219	20.67	7.2	0.45	5.728	20.19	7.0	25		34	19	6.97
	6	5.992	219	20.31	7.7	1.53	5.806	19.11	7.4	25		14	6	7.01
6Q	7	5.057	223	15.63	9.4	0.17	5.029	15.45	9.2	34	42	23	38	6.68
	8	5.175	233	15.69	9.4	0.15	5.147	15.53	9.1	16	23	24	46	6.84
	9	5.126	240	14.99	9.8	0.16	5.083	14.76	9.6	35	52	32	63	6.72
	10	5.031	240	14.47	10.4	0.63	4.937	13.96	10.2	30	56	34	80	6.81
	11	5.064	226	15.47	9.5	0.16	5.039	15.33	9.3	31	61	34	66	6.75
6Q	12	4.752	235	13.23	10.9	0.33	4.647	12.68	11.2	53	53	68	21	6.84
	13	4.846	246	13.17	11.0	0.33	4.744	12.65	11.2	35	52	68	6	6.72
	14	4.923	244	13.68	11.0	1.19	4.779	12.93	10.9	31	35	19		6.81
	15	4.697	235	12.94	11.2	0.34	4.621	12.54	11.3	47	65	43		6.78
7Q	16	4.944	230	14.55	9.7	2.5	4.673	13.06	10.9	37	21	30	28	6.73
	17	5.094	245	14.57	9.7	3.08	4.746	12.73	11.2	46	34	38	42	6.75
	18	5.119	244	14.73	9.6	3.08	4.715	12.60	11.3	32	31	20	23	6.81
	19	5.043	246	14.19	10.0	2.5	4.731	13.57	11.3	48	42	38	29	6.78
	20	4.991	245	13.96	10.2	1.43	4.85	13.22	10.7	47	35	26	28	6.68
	21	5.058	244	14.41	9.8	2.5	4.82	13.14	10.8	60	22	52	28	6.71
7Q_2	22	4.669	233	12.91	11.0	0.29	4.64	12.76	11.1	28	23	25	31	6.75
	23	4.673	230	13.45	10.5	0.28	4.63	12.84	11.1	30	28	42	45	6.78
	24	4.85	245	13.22	10.7	0.43	4.78	12.88	11.0	26	28	28	28	6.68
	25	4.82	244	13.14	10.8	0.27	4.78	12.93	10.9	28	28	50	39	6.71

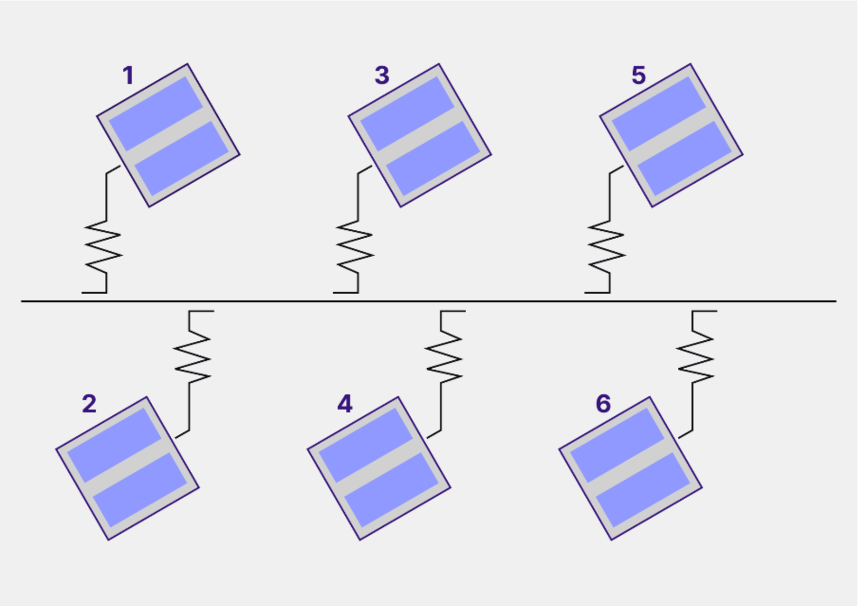

Figure S22 Circuit diagram of the 1Q chip

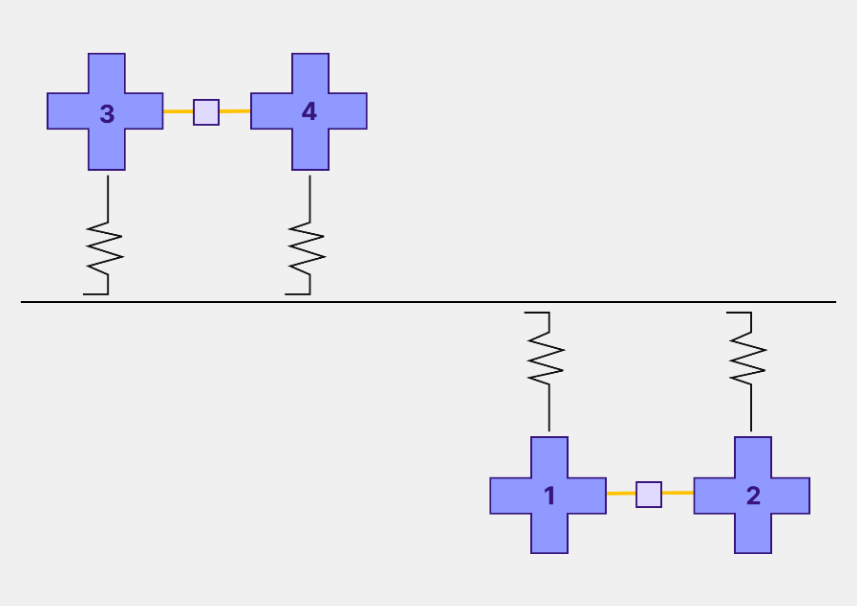

Figure S23 Circuit diagram of the 2Q chip

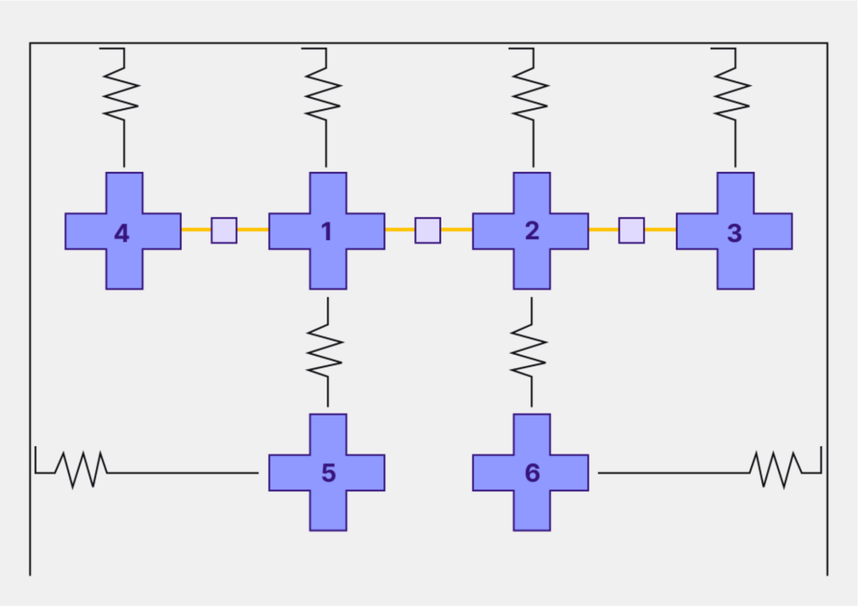

Figure S24 Circuit diagram of the 2Q chip

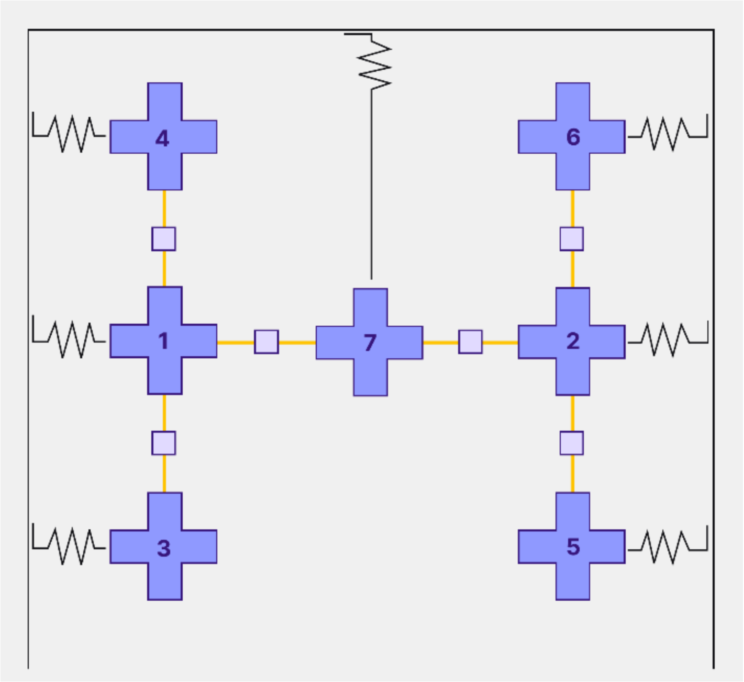

Figure S25 Circuit diagram of the 7Q chip

Table S8: Measurement data for 1Q chip

Q_N	$T_{1,before}$, μs	$T_{1,after}$, μs
1	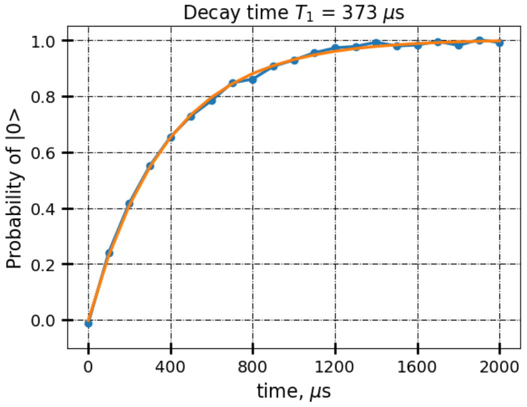	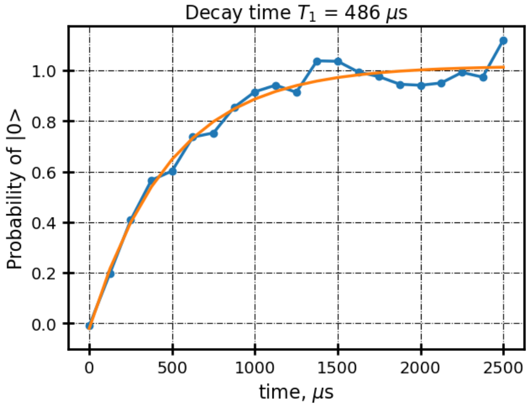
	$T_{2E,before}$, μs	$T_{2E,after}$, μs
	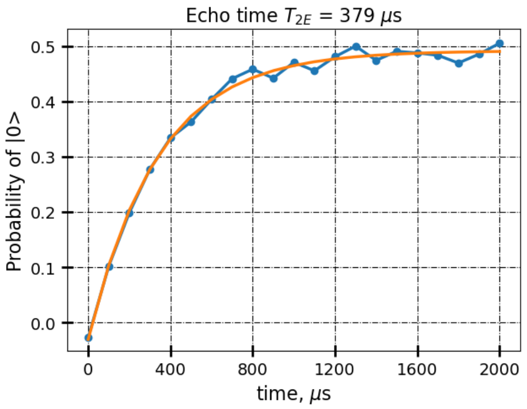	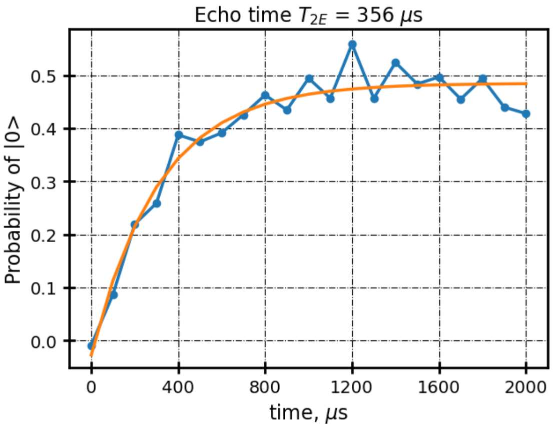
	Ramsey $T_{2,before}$, μs	Ramsey $T_{2,after}$, μs
	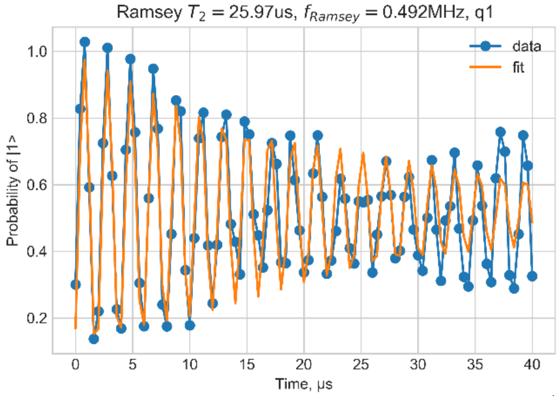	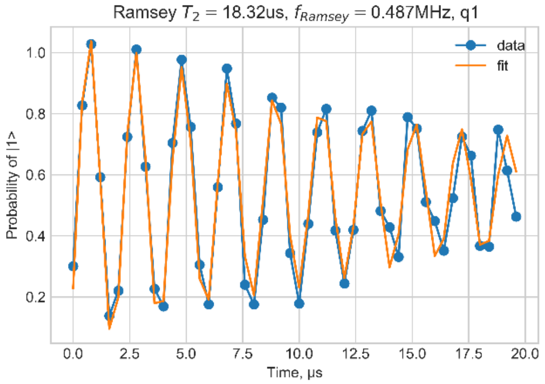
6	$T_{1,before}$, μs	$T_{1,after}$, μs

<table>
<tr><td rowspan="5"></td><td>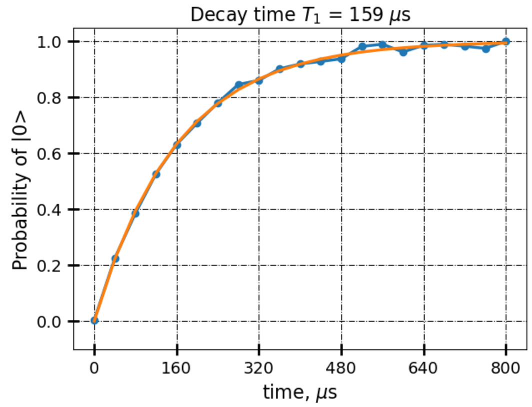
</td><td>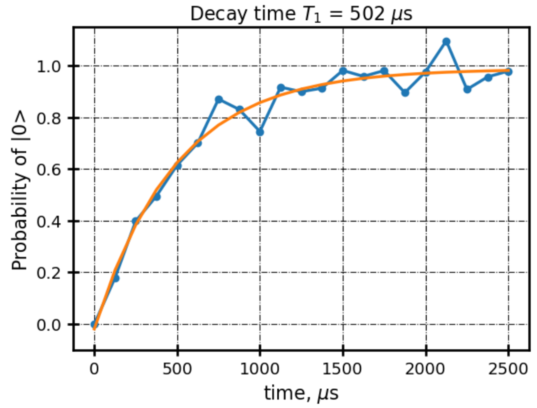
</td></tr>
<tr><td>$T_{2E,before}$, µs</td><td>$T_{2E,after}$, µs</td></tr>
<tr><td>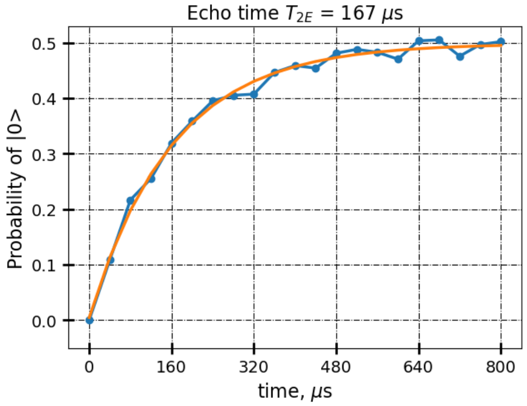
</td><td>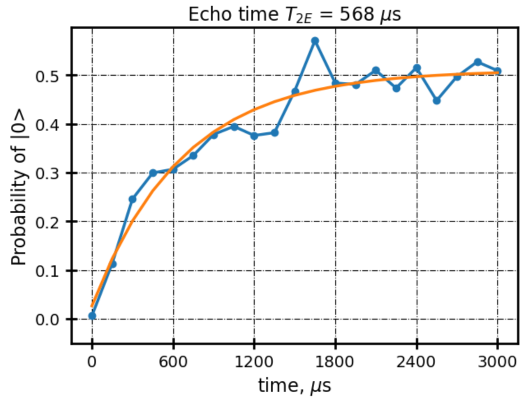
</td></tr>
<tr><td>Ramsey $T_{2,before}$, µs</td><td>Ramsey $T_{2,after}$, µs</td></tr>
<tr><td>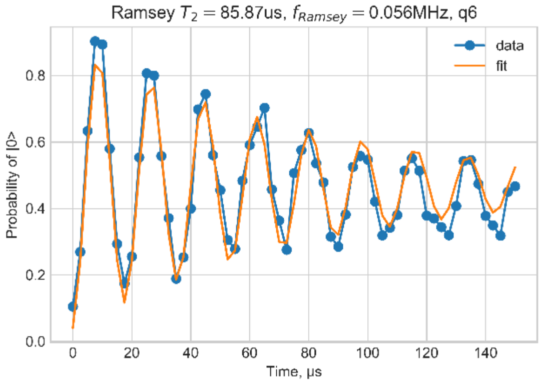
</td><td>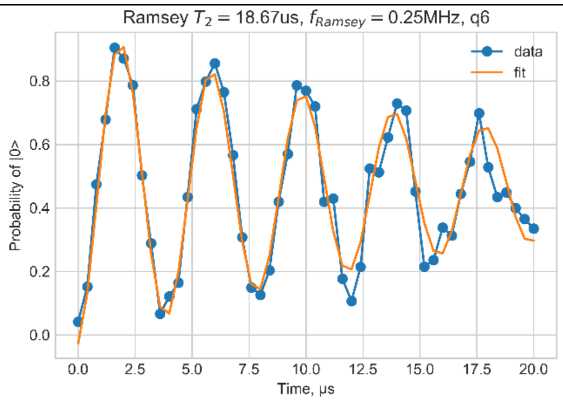
</td></tr>
</table>

Table S9: Measurement data for 7Q chip

First annealing		
**Q N**	**$T_{1,before}$, µs**	**$T_{1,after}$, µs**
**2**	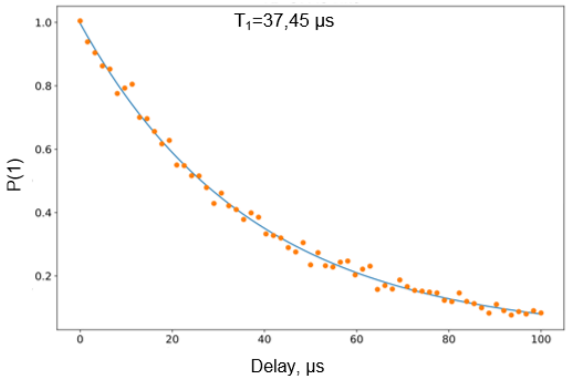	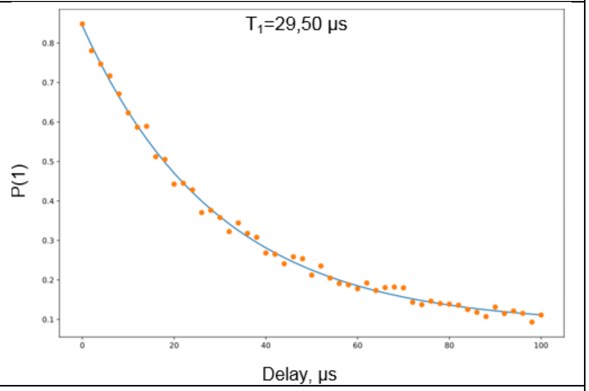
	**$T_{2E,before}$, µs**	**$T_{2E,after}$, µs**
	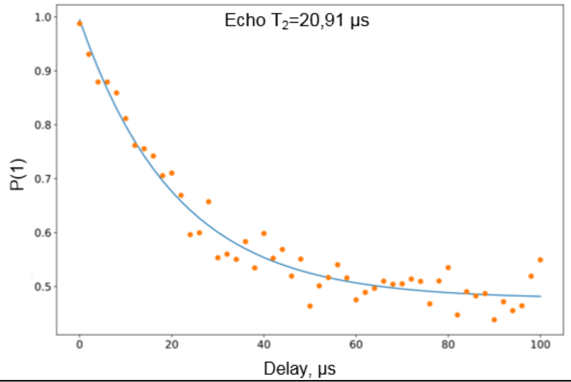	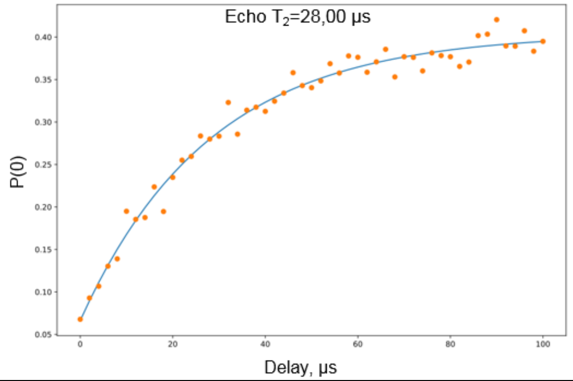
	**Ramsey $T_{2,before}$, µs**	**Ramsey $T_{2,after}$, µs**
	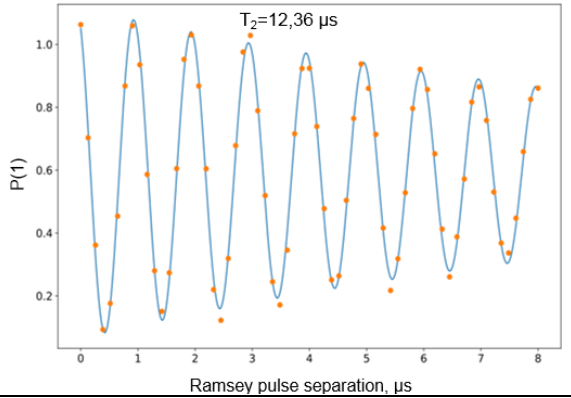	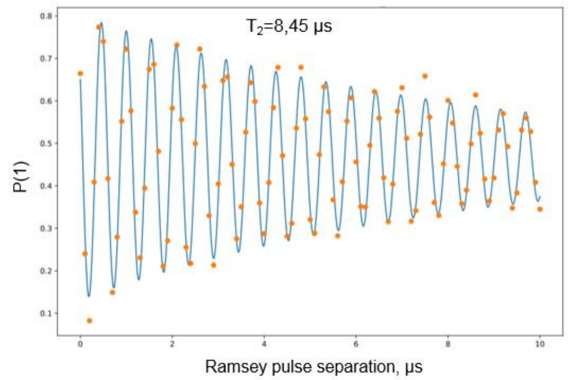
**3**	**$T_{1,before}$, µs**	**$T_{1,after}$, µs**

<table>
<tr><td rowspan="5"></td><td>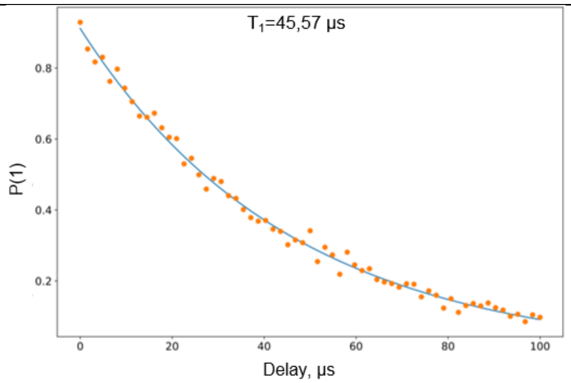
</td><td>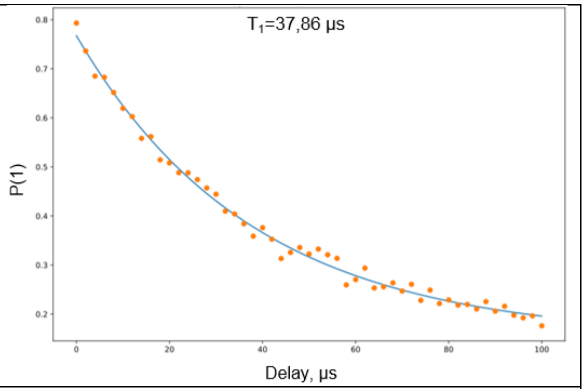
</td></tr>
<tr><td>$T_{2E,before}$, µs</td><td>$T_{2E,after}$, µs</td></tr>
<tr><td>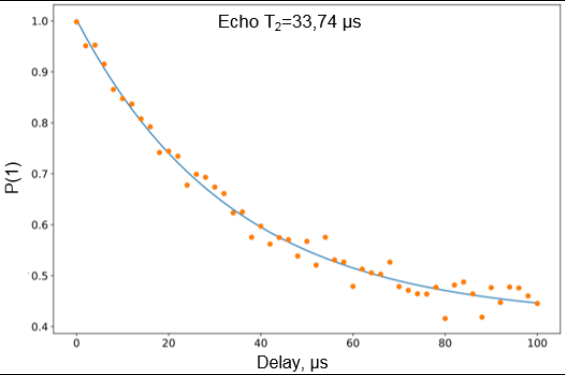
</td><td>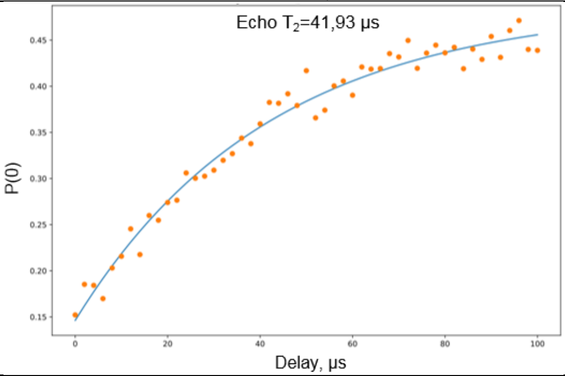
</td></tr>
<tr><td>Ramsey $T_{2,before}$, µs</td><td>Ramsey $T_{2,after}$, µs</td></tr>
<tr><td>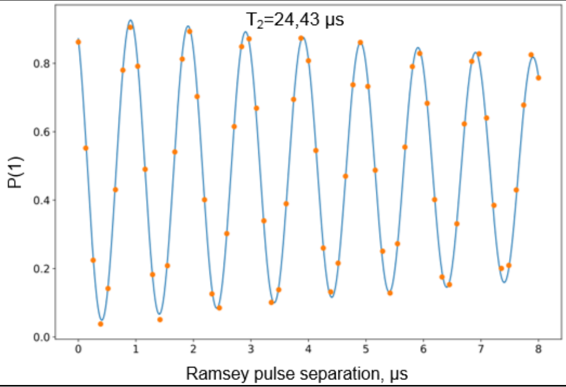
</td><td>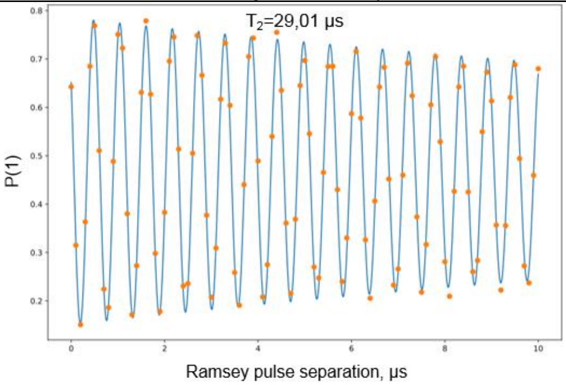
</td></tr>
<tr><td rowspan="3">4</td><td>$T_{1,before}$, µs</td><td>$T_{1,after}$, µs</td></tr>
<tr><td>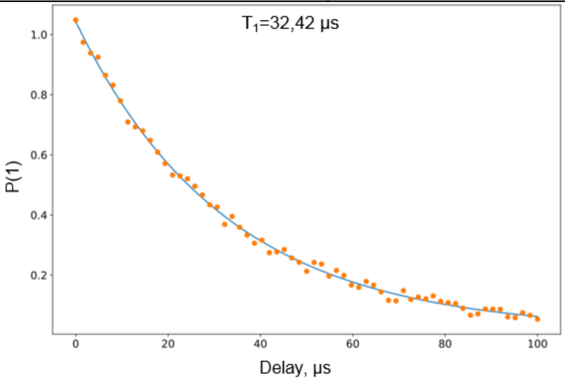
</td><td>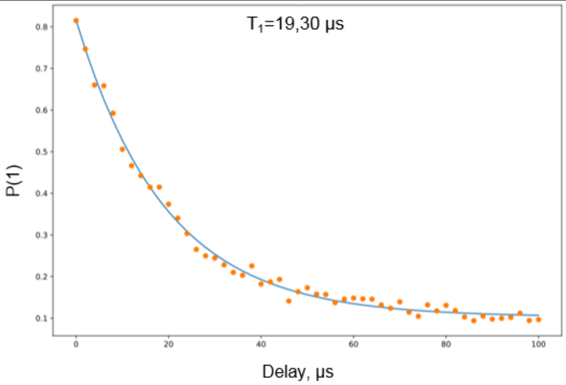
</td></tr>
<tr><td>$T_{2E,before}$, µs</td><td>$T_{2E,after}$, µs</td></tr>
</table>

	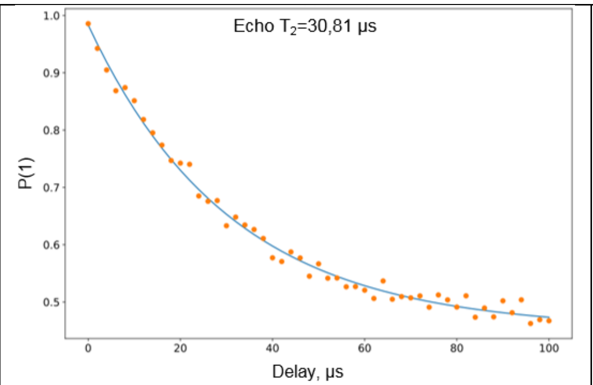	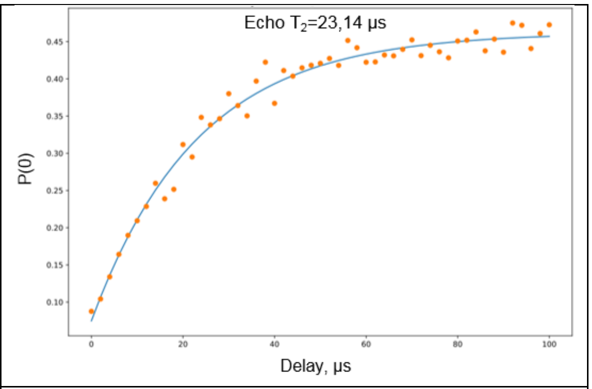
	**Ramsey $T_{2,before}$, μs**	**Ramsey $T_{2,after}$, μs**
	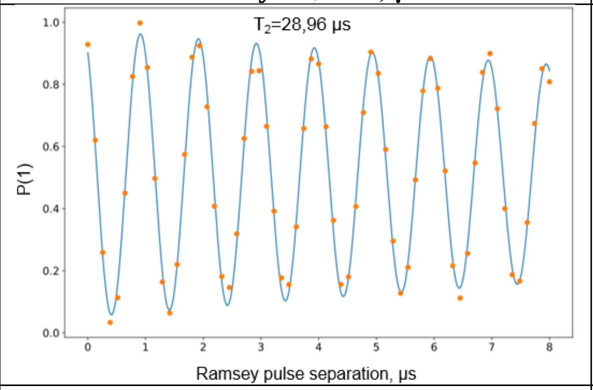	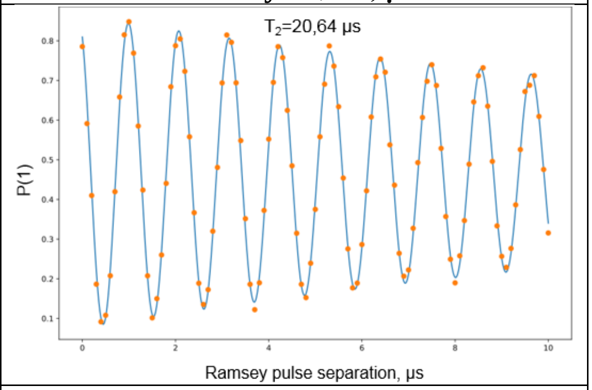
5	**$T_{1,before}$, μs**	**$T_{1,after}$, μs**
	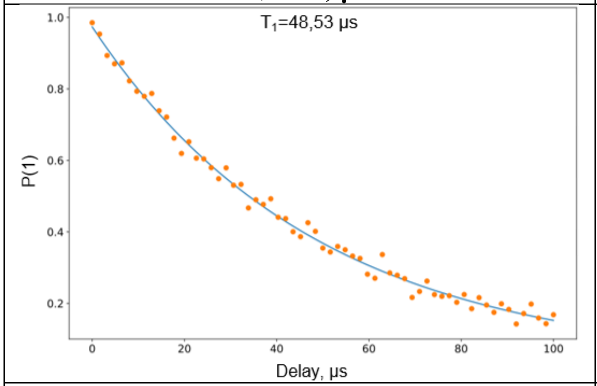	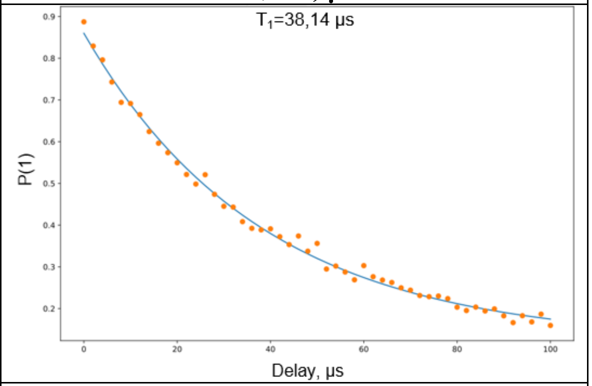
	**$T_{2E,before}$, μs**	**$T_{2E,after}$, μs**
	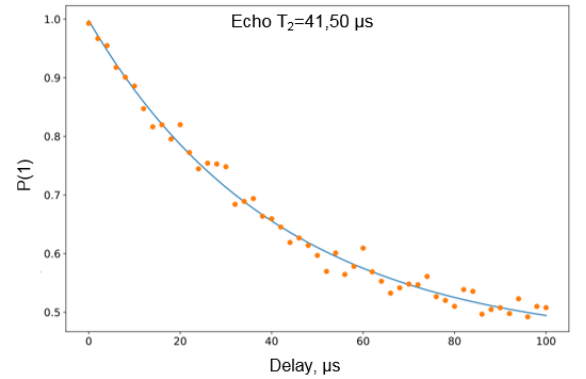	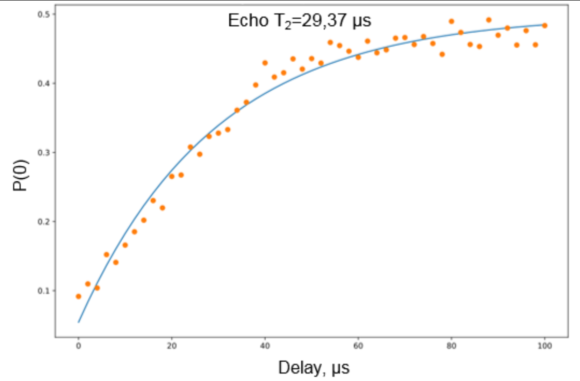
	**Ramsey $T_{2,before}$, μs**	**Ramsey $T_{2,after}$, μs**

	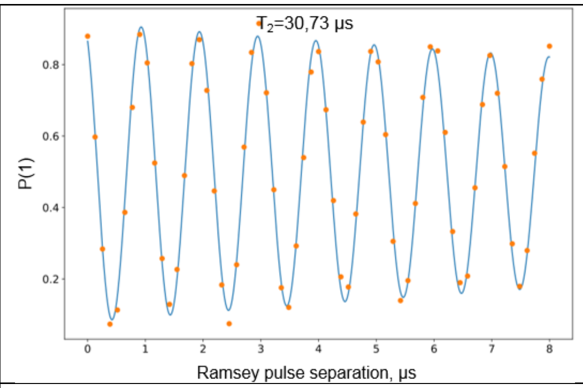	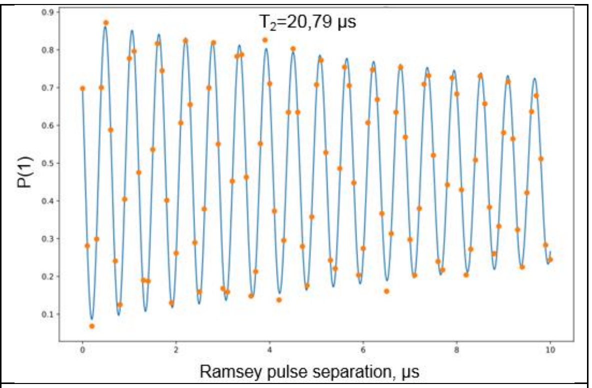
6	**$T_{1,before}$, μs**	**$T_{1,after}$, μs**
	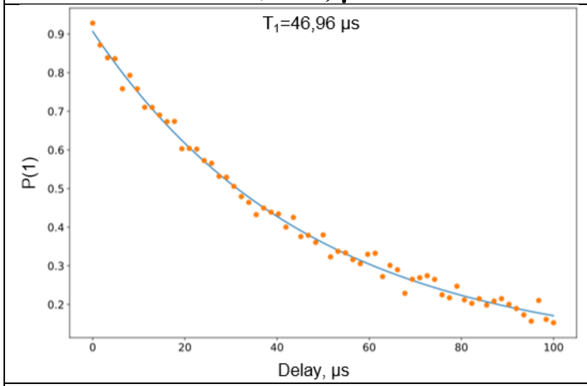	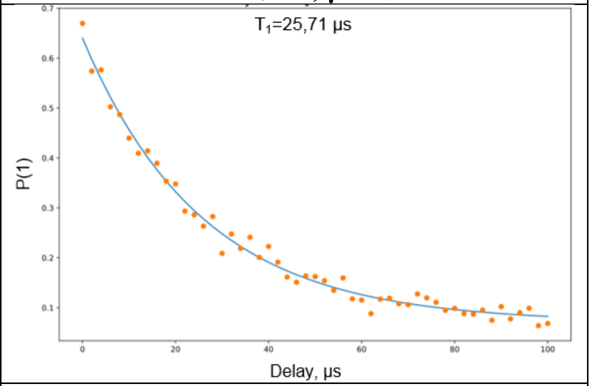
	**$T_{2E,before}$, μs**	**$T_{2E,after}$, μs**
	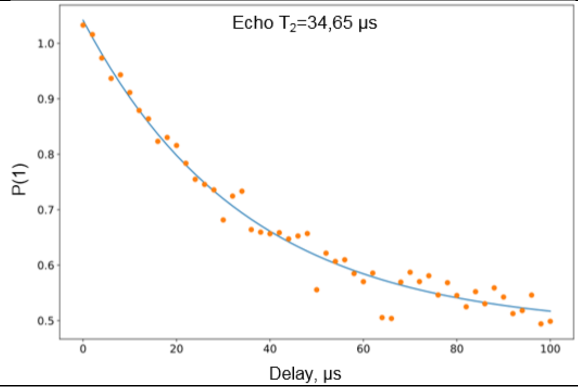	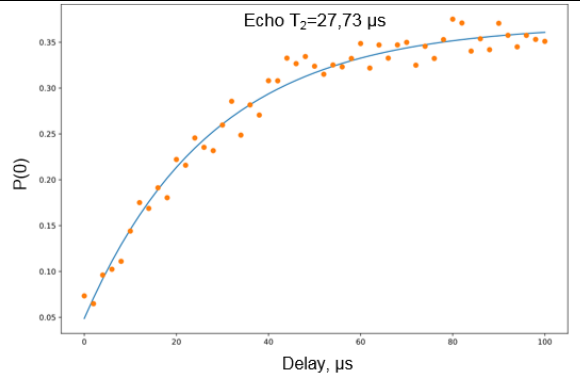
	**Ramsey $T_{2,before}$, μs**	**Ramsey $T_{2,after}$, μs**
	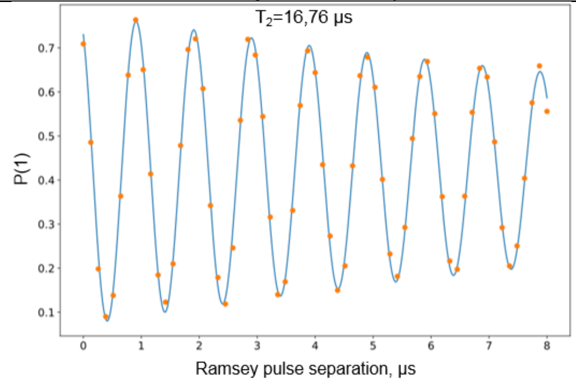	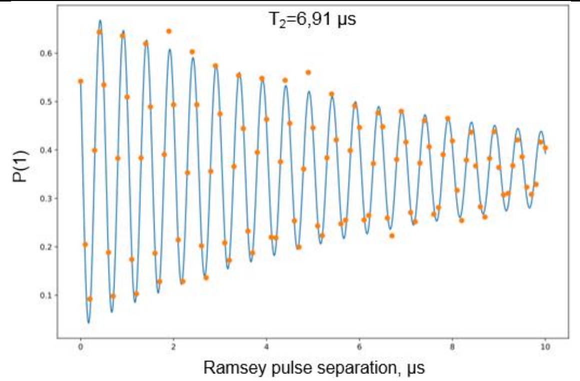
7	**$T_{1,before}$, μs**	**$T_{1,after}$, μs**

<table>
<tr><td></td><td>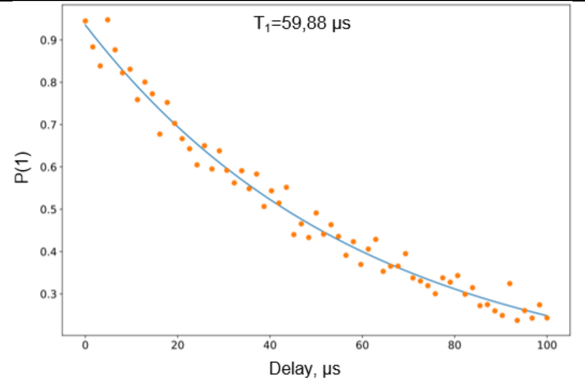</td><td></td></tr>
<tr><td></td><td>T$_{2E,before}$, µs</td><td>T$_{2E,after}$, µs</td></tr>
<tr><td></td><td>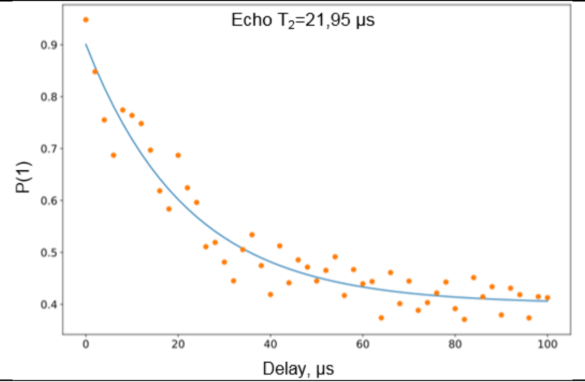</td><td></td></tr>
<tr><td></td><td>Ramsey T$_{2,before}$, µs</td><td>Ramsey T$_{2,after}$, µs</td></tr>
<tr><td></td><td>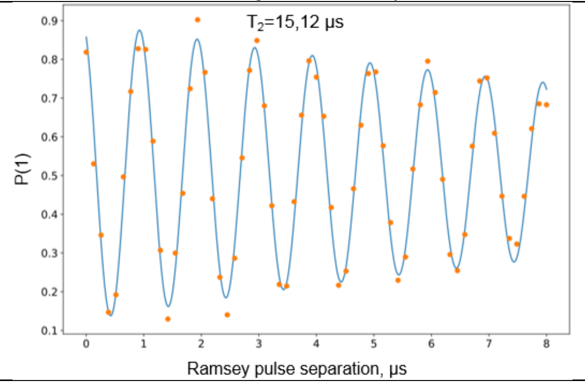</td><td></td></tr>
<tr><td colspan="3">Second annealing</td></tr>
<tr><td>Q
N</td><td>T$_{1,before}$, µs</td><td>T$_{1,after}$, µs</td></tr>
<tr><td>1</td><td>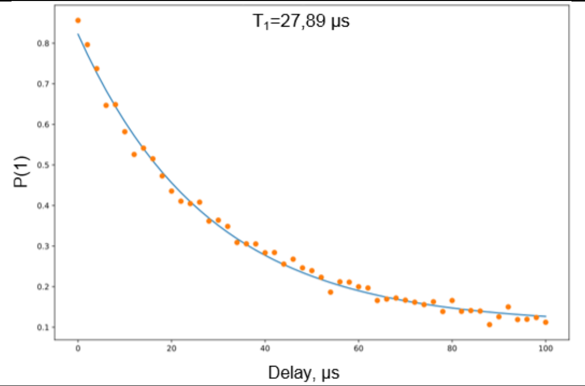</td><td>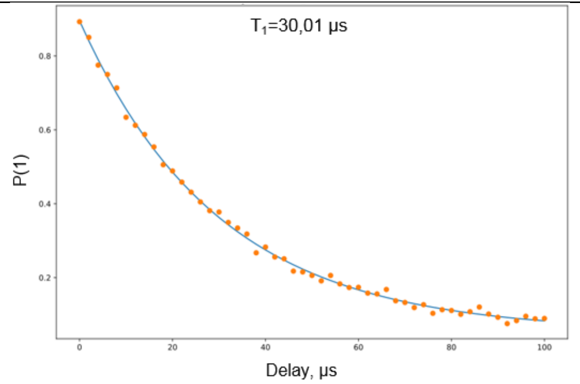</td></tr>
</table>

<table>
<tr><td></td><td>$T_{2E,before}$, µs</td><td>$T_{2E,after}$, µs</td></tr>
<tr><td></td><td>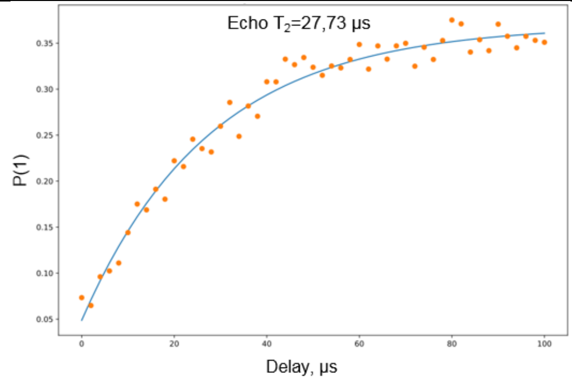
</td><td>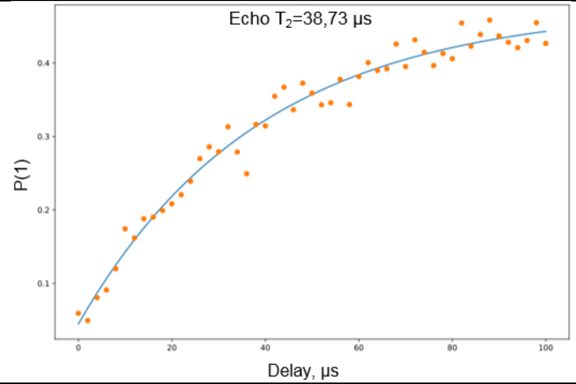
</td></tr>
<tr><td></td><td>Ramsey $T_{2,before}$, µs</td><td>Ramsey $T_{2,after}$, µs</td></tr>
<tr><td></td><td>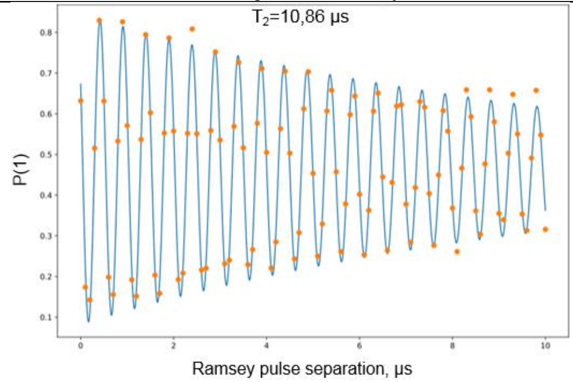
</td><td>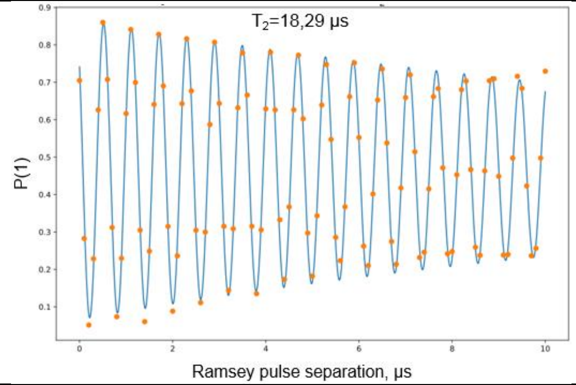
</td></tr>
<tr><td rowspan="5">2</td><td>$T_{1,before}$, µs</td><td>$T_{1,after}$, µs</td></tr>
<tr><td>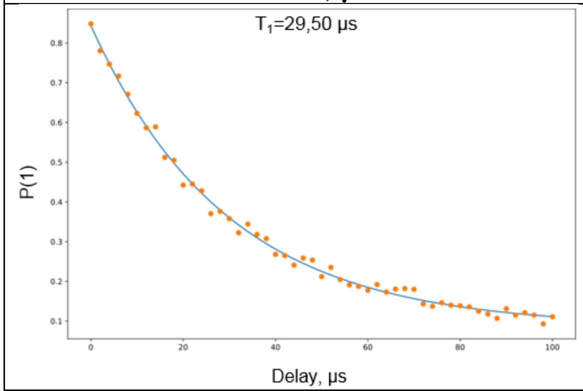
</td><td>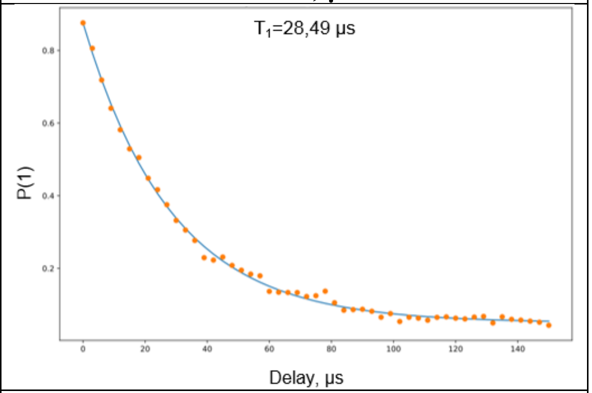
</td></tr>
<tr><td>$T_{2E,before}$, µs</td><td>$T_{2E,after}$, µs</td></tr>
<tr><td>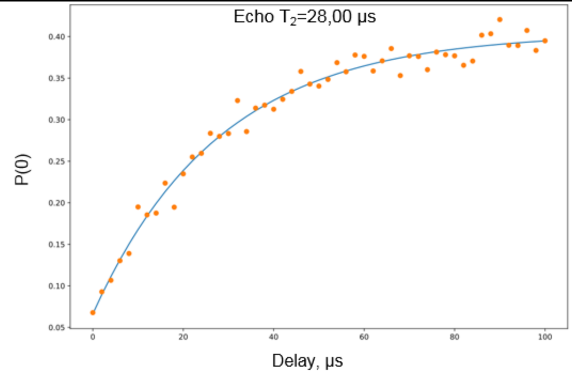
</td><td>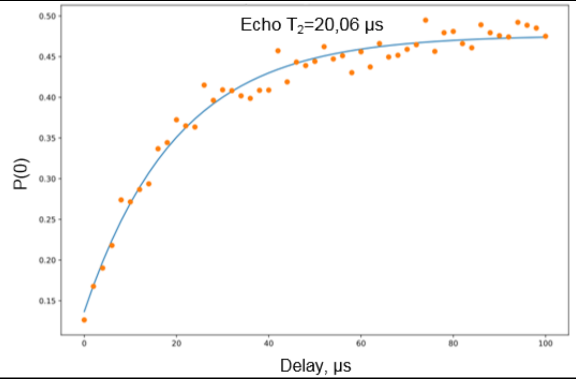
</td></tr>
<tr><td>Ramsey $T_{2,before}$, µs</td><td>Ramsey $T_{2,after}$, µs</td></tr>
</table>

	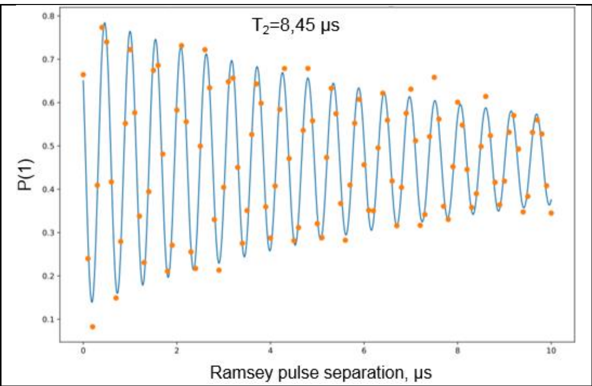	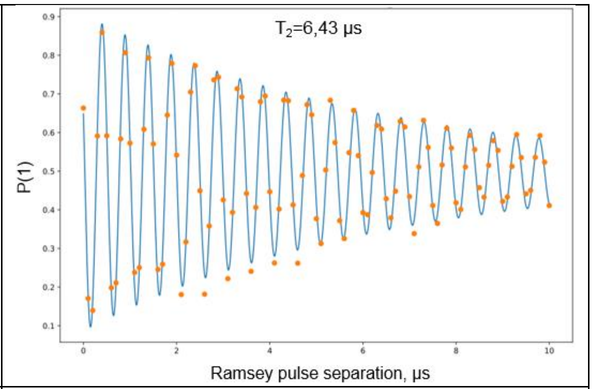
**6**	**$T_{1,before}$, μs**	**$T_{1,after}$, μs**
	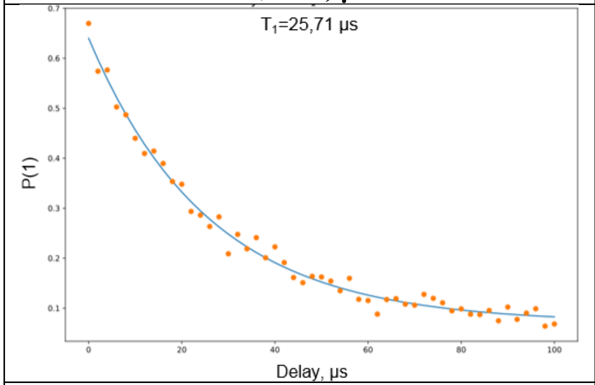	
	**$T_{2E,before}$, μs**	**$T_{2E,after}$, μs**
	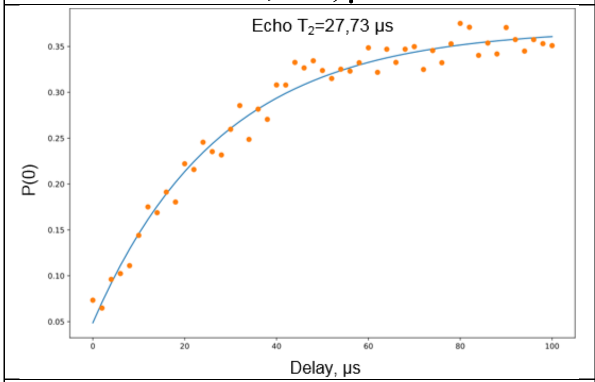	
	**Ramsey $T_{2,before}$, μs**	**Ramsey $T_{2,after}$, μs**
	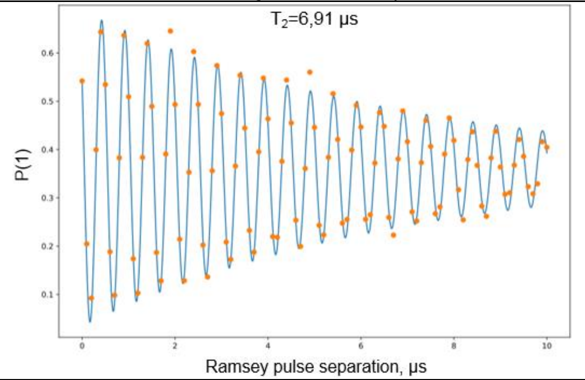	
**7**	**$T_{1,before}$, μs**	**$T_{1,after}$, μs**

<table>
<tr><td></td><td></td><td>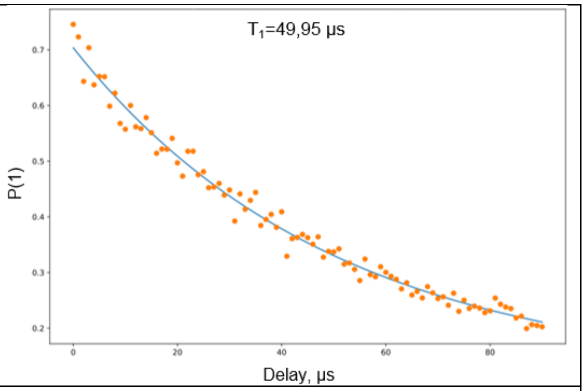
</td></tr>
<tr><td></td><td>$T_{2E,before}$, μs</td><td>$T_{2E,after}$, μs</td></tr>
<tr><td></td><td></td><td>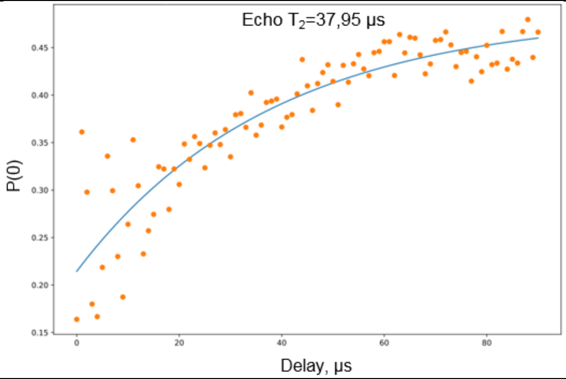
</td></tr>
<tr><td></td><td>Ramsey $T_{2,before}$, μs</td><td>Ramsey $T_{2,after}$, μs</td></tr>
<tr><td></td><td></td><td></td></tr>
<tr><td></td><td></td><td>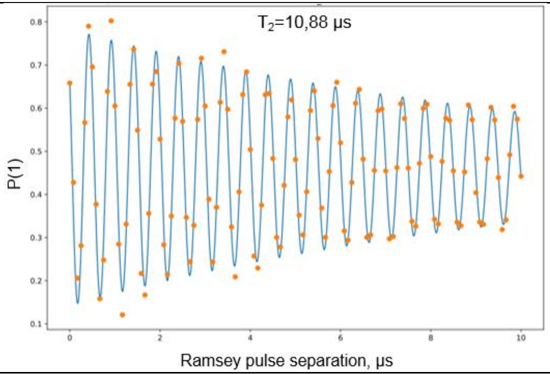
</td></tr>
</table>

Table S10: the change in the frequency of the annealed qubits due to aging after 14 days

f_{before}, GHz	f_{after}, GHz	Δf, MHz	f_{aged}, GHz	Δf_{aged}, MHz	$T_{1,before}$, µs	$T_{1,after}$, µs	$T_{1,aged}$, µs	$T_{2,before}$, µs	$T_{2,after}$, µs	$T_{2,aged}$, µs
5,094	4,746	348	4,725	20	45	38	42	34	42	57
5,119	4,715	404	4,688	26	32	20	46	31	23	43
5,043	4,731	312	4,713	17	48	38	40	42	29	39

Where f is the qubit frequency.

Movie S1.
Full-scale molecular dynamics simulation.

Movie S2.
Molecular dynamics simulation.

REFERENCES AND NOTES

1. F. Arute, K. Arya, R. Babbush, D. Bacon, J. C. Bardin, R. Barends, R. Biswas, S. Boixo, F. G. S. L. Brandao, D. A. Buell, B. Burkett, Y. Chen, Z. Chen, B. Chiaro, R. Collins, W. Courtney, A. Dunsworth, E. Farhi, B. Foxen, A. Fowler, C. Gidney, M. Giustina, R. Graff, K. Guerin, S. Habegger, M. P. Harrigan, M. J. Hartmann, A. Ho, M. Hoffmann, T. Huang, T. S. Humble, S. V. Isakov, E. Jeffrey, Z. Jiang, D. Kafri, K. Kechedzhi, J. Kelly, P. V. Klimov, S. Knysh, A. Korotkov, F. Kostritsa, D. Landhuis, M. Lindmark, E. Lucero, D. Lyakh, S. Mandrà, J. R. McClean, M. McEwen, A. Megrant, X. Mi, K. Michielsen, M. Mohseni, J. Mutus, O. Naaman, M. Neeley, C. Neill, M. Y. Niu, E. Ostby, A. Petukhov, J. C. Platt, C. Quintana, E. G. Rieffel, P. Roushan, N. C. Rubin, D. Sank, K. J. Satzinger, V. Smelyanskiy, K. J. Sung, M. D. Trevithick, A. Vainsencher, B. Villalonga, T. White, Z. J. Yao, P. Yeh, A. Zalcman, H. Neven, J. M. Martinis, Quantum supremacy using a programmable superconducting processor. *Nature* **574**, 505–510 (2019).

2. V. Havlíček, A. D. Córcoles, K. Temme, A. W. Harrow, A. Kandala, J. M. Chow, J. M. Gambetta, Supervised learning with quantum-enhanced feature spaces. *Nature* **567**, 209–212 (2019).

3. H.-Y. Huang, M. Broughton, J. Cotler, S. Chen, J. Li, M. Mohseni, H. Neven, R. Babbush, R. Kueng, J. Preskill, J. R. McClean, Quantum advantage in learning from experiments. *Science* **376**, 1182–1186 (2022).

4. A. Fert, N. Reyren, V. Cros, Magnetic skyrmions: Advances in physics and potential applications. *Nat. Rev. Mater.* **2**, 17031 (2017).

5. K. Meng, Z. Li, P. Chen, X. Ma, J. Huang, J. Li, F. Qin, C. Qiu, Y. Zhang, D. Zhang, Y. Deng, Y. Yang, G. Gu, H. Y. Hwang, Q.-K. Xue, Y. Cui, H. Yuan, Superionic fluoride gate dielectrics with low diffusion barrier for two-dimensional electronics. *Nat. Nanotechnol.* **19**, 932–940 (2024).

6. M. Gurvitch, M. A. Washington, H. A. Huggins, High quality refractory Josephson tunnel junctions utilizing thin aluminum layers. *Appl. Phys. Lett.* **42**, 472–474 (1983).

7. D. Olaya, M. Castellanos-Beltran, J. Pulecio, J. Biesecker, S. Khadem, T. Lewitt, P. Hopkins, P. Dresselhaus, S. Benz, Planarized process for single-flux-quantum circuits with self-shunted Nb/Nb_xSi_{1-x}/Nb Josephson junctions. *IEEE Trans. Appl. Supercond.* **29**, 1101708 (2019).

8. S. K. Tolpygo, Superconductor digital electronics: Scalability and energy efficiency issues (review article). *Low Temp. Phys.* **42**, 361–379 (2016).

9. M. Kjaergaard, M. E. Schwartz, J. Braumüller, P. Krantz, J. I.-J. Wang, S. Gustavsson, W. D. Oliver, Superconducting qubits: Current state of play. *Annu. Rev. Condens. Matter. Phys.* **11**, 369–395 (2020).

10. J. B. Hertzberg, E. J. Zhang, S. Rosenblatt, E. Magesan, J. A. Smolin, J.-B. Yau, V. P. Adiga, M. Sandberg, M. Brink, J. M. Chow, J. S. Orcutt, Laser-annealing Josephson junctions for yielding scaled-up superconducting quantum processors. *npj Quantum Inf.* **7**, 129 (2021).

11. E. J. Zhang, S. Srinivasan, N. Sundaresan, D. F. Bogorin, Y. Martin, J. B. Hertzberg, J. Timmerwilke, E. J. Pritchett, J.-B. Yau, C. Wang, W. Landers, E. P. Lewandowski, A. Narasgond, S. Rosenblatt, G. A. Keefe, I. Lauer, M. B. Rothwell, D. T. McClure, O. E. Dial, J. S. Orcutt, M. Brink, J. M. Chow, High-performance superconducting quantum processors via laser annealing of transmon qubits. *Sci. Adv.* **8**, doi: 10.1126/sciadv.abi6690 (2022).

12. D. A. Moskaleva, N. D. Korshakov, D. O. Moskalev, A. A. Solovyova, A. R. Matanin, E. I. Malevannaya, N. S. Smirnov, M. I. Teleganov, Y. V. Panfilov, I. A. Rodionov, Wafer-scale uniformity improvement of Dolan-bridge Josephson junction by shadow evaporation bias correction. arXiv:2403.01894 [quant-ph] (2024).

13. D. O. Moskalev, E. V. Zikiy, A. A. Pishchimova, D. A. Ezenkova, N. S. Smirnov, A. I. Ivanov, N. D. Korshakov, I. A. Rodionov, Optimization of shadow evaporation and oxidation for reproducible quantum Josephson junction circuits. *Sci. Rep.* **13**, 4174 (2023).

14. A. A. Pishchimova, N. S. Smirnov, D. A. Ezenkova, E. A. Krivko, E. V. Zikiy, D. O. Moskalev, A. I. Ivanov, N. D. Korshakov, I. A. Rodionov, Improving Josephson junction reproducibility for superconducting quantum circuits: Junction area fluctuation. *Sci. Rep.* **13**, 6772 (2023).

15. J. M. Kreikebaum, K. P. O'Brien, A. Morvan, I. Siddiqi, Improving wafer-scale Josephson junction resistance variation in superconducting quantum coherent circuits. *Supercond. Sci. Technol.* **33**, 06LT02 (2020).

16. N. Muthusubramanian, M. Finkel, P. Duivestein, C. Zachariadis, S. L. M. van der Meer, H. M. Veen, M. W. Beekman, T. Stavenga, A. Bruno, L. DiCarlo, Wafer-scale uniformity of Dolan-bridge and bridgeless Manhattan-style Josephson junctions for superconducting quantum processors. *Quantum Sci. Technol.* **9**, 025006 (2024).

17. Y. Balaji, N. Acharya, R. Armstrong, K. G. Crawford, S. Danilin, T. Dixon, O. W. Kennedy, R. D. Pothuraju, K. Shahbazi, C. D. Shelly, Electron-beam annealing of Josephson junctions for frequency tuning of quantum processors. arXiv:2402.17395v2 [quant-ph] (2024).

18. D. P. Pappas, M. Field, C. J. Kopas, J. A. Howard, X. Wang, E. Lachman, J. Oh, L. Zhou, A. Gold, G. M. Stiehl, K. Yadavalli, E. A. Sete, A. Bestwick, M. J. Kramer, J. Y. Mutus, Alternating-bias assisted annealing of amorphous oxide tunnel junctions. *Commun. Mater.* **5**, 150 (2024).

19. X. Wang, J. Howard, E. A. Sete, G. Stiehl, C. Kopas, S. Poletto, X. Wu, M. Field, N. Sharac, C. Eckberg, H. Cansizoglu, R. Katta, J. Mutus, A. Bestwick, K. Yadavalli, D. P. Pappas, Precision frequency tuning of tunable transmon qubits using alternating-bias assisted annealing. arXiv:2407.06425 [quant-ph] (2024).

20. A. R. Matanin, K. I. Gerasimov, E. S. Moiseev, N. S. Smirnov, A. I. Ivanov, E. I. Malevannaya, V. I. Polozov, E. V. Zikiy, A. A. Samoilov, I. A. Rodionov, S. A. Moiseev, Toward highly efficient multimode superconducting quantum memory. *Phys. Rev. Appl.* **19**, 034011 (2023).

21. N. E. Frattini, V. V. Sivak, A. Lingenfelter, S. Shankar, M. H. Devoret, Optimizing the nonlinearity and dissipation of a SNAIL parametric amplifier for dynamic range. *Phys. Rev. Appl.* **10**, 054020 (2018).

22. D. Ezenkova, D. Moskalev, N. Smirnov, A. Ivanov, A. Matanin, V. Polozov, V. Echeistov, E. Malevannaya, A. Samoylov, E. Zikiy, I. Rodionov, Broadband SNAIL parametric amplifier with microstrip impedance transformer. *Appl. Phys. Lett.* **121**, 232601 (2022).

23. R. Assouly, R. Dassonneville, T. Peronnin, A. Bienfait, B. Huard, Quantum advantage in microwave quantum radar. *Nat. Phys.* **19**, 1418–1422 (2023).

24. P. Gkoupidenis, D. A. Koutsouras, G. G. Malliaras, Neuromorphic device architectures with global connectivity through electrolyte gating. *Nat. Commun.* **8**, 15448 (2017).

25. D. Sarkar, X. Xie, W. Liu, W. Cao, J. Kang, Y. Gong, S. Kraemer, P. M. Ajayan, K. Banerjee, A subthermionic tunnel field-effect transistor with an atomically thin channel. *Nature* **526**, 91–95 (2015).

26. S. Kumar, R. S. Williams, Z. Wang, Third-order nanocircuit elements for neuromorphic engineering. *Nature* **585**, 518–523 (2020).

27. K.-H. Kim, S. Oh, M. M. A. Fiagbenu, J. Zheng, P. Musavigharavi, P. Kumar, N. Trainor, A. Aljarb, Y. Wan, H. M. Kim, K. Katti, S. Song, G. Kim, Z. Tang, J.-H. Fu, M. Hakami, V. Tung, J. M. Redwing, E. A. Stach, R. H. Olsson, D. Jariwala, Scalable CMOS back-end-of-line-compatible AlScN/two-dimensional channel ferroelectric field-effect transistors. *Nat. Nanotechnol.* **18**, 1044–1050 (2023).

28. V. V. Kornienko, I. A. Nechepurenko, P. N. Tananaev, E. D. Chubchev, A. S. Baburin, V. V. Echeistov, A. V. Zverev, I. I. Novoselov, I. A. Kruglov, I. A. Rodionov, A. V. Baryshev, A. V. Dorofeenko, Machine learning for optical gas sensing: A leaky-mode humidity sensor as example. *IEEE Sens. J.* **20**, 6954–6963 (2020).

29. C. Wang, X. Li, H. Xu, Z. Li, J. Wang, Z. Yang, Z. Mi, X. Liang, T. Su, C. Yang, G. Wang, W. Wang, Y. Li, M. Chen, C. Li, K. Linghu, J. Han, Y. Zhang, Y. Feng, Y. Song, T. Ma, J. Zhang, R. Wang, P. Zhao, W. Liu, G. Xue, Y. Jin, H. Yu, Towards practical quantum computers: Transmon qubit with a lifetime approaching 0.5 milliseconds. *npj Quantum Inf.* **8**, 3 (2022).

30. E. Mitterreiter, B. Schuler, A. Micevic, D. Hernangómez-Pérez, K. Barthelmi, K. A. Cochrane, J. Kiemle, F. Sigger, J. Klein, E. Wong, E. S. Barnard, K. Watanabe, T. Taniguchi, M. Lorke, F. Jahnke, J. J. Finley, A. M. Schwartzberg, D. Y. Qiu, S. Refaely-Abramson, A. W. Holleitner, A. Weber-Bargioni, C. Kastl, The role of chalcogen vacancies for atomic defect emission in MoS_2. *Nat. Commun.* **12**, 3822 (2021).

31. Y. Jia, Q. Yang, Y.-W. Fang, Y. Lu, M. Xie, J. Wei, J. Tian, L. Zhang, R. Yang, Giant tunnelling electroresistance in atomic-scale ferroelectric tunnel junctions. *Nat. Commun.* **15**, 693 (2024).

32. J. F. Ziegler, M. D. Ziegler, J. P. Biersack, SRIM—The stopping and range of ions in matter (2010). *Nucl. Instrum. Methods Phys. Res. B* **268**, 1818–1823 (2010).

33. I. I. Novoselov, A. V. Yanilkin, A. V. Shapeev, E. V. Podryabinkin, Moment tensor potentials as a promising tool to study diffusion processes. *Comput. Mater. Sci.* **164**, 46–56 (2019).

34. R. Bullough, M. H. Wood, Mechanisms of radiation induced creep and growth. *J. Nucl. Mater.* **90**, 1–21 (1980).

35. V. Ambegaokar, A. Baratoff, Tunneling between superconductors. *Phys. Rev. Lett.* **10**, 486–489 (1963).

36. J. Koch, T. M. Yu, J. Gambetta, A. A. Houck, D. I. Schuster, J. Majer, A. Blais, M. H. Devoret, S. M. Girvin, R. J. Schoelkopf, Charge-insensitive qubit design derived from the Cooper pair box. *Phys. Rev. A* **76**, 042319 (2007).

37. E. V. Zikiy, A. I. Ivanov, N. S. Smirnov, D. O. Moskalev, V. I. Polozov, A. R. Matanin, E. I. Malevannaya, V. V. Echeistov, T. G. Konstantinova, I. A. Rodionov, High-Q trenched aluminum coplanar resonators with an ultrasonic edge microcutting for superconducting quantum devices. *Sci. Rep.* **13**, 15536 (2023).

38. P. V. Klimov, J. Kelly, Z. Chen, M. Neeley, A. Megrant, B. Burkett, R. Barends, K. Arya, B. Chiaro, Y. Chen, A. Dunsworth, A. Fowler, B. Foxen, C. Gidney, M. Giustina, R. Graff, T. Huang, E. Jeffrey, E. Lucero, J. Y. Mutus, O. Naaman, C. Neill, C. Quintana, P. Roushan, D.

Sank, A. Vainsencher, J. Wenner, T. C. White, S. Boixo, R. Babbush, V. N. Smelyanskiy, H. Neven, J. M. Martinis, Fluctuations of energy-relaxation times in superconducting qubits. *Phys. Rev. Lett.* **121**, 090502 (2018).

39. W. A. Phillips, Tunneling states in amorphous solids. *J. Low Temp. Phys.* **7**, 351–360 (1972).

40. P. w. Anderson, B. I. Halperin, c. M. Varma, Anomalous low-temperature thermal properties of glasses and spin glasses. *Philos. Mag.* **25**, 1–9 (1972).

41. B. A. Primavera, J. M. Shainline, An active dendritic tree can mitigate fan-in limitations in superconducting neurons. *Appl. Phys. Lett.* **119** (2021).

42. Z. Wang, S. Joshi, S. E. Savel'ev, H. Jiang, R. Midya, P. Lin, M. Hu, N. Ge, J. P. Strachan, Z. Li, Q. Wu, M. Barnell, G.-L. Li, H. L. Xin, R. S. Williams, Q. Xia, J. J. Yang, Memristors with diffusive dynamics as synaptic emulators for neuromorphic computing. *Nat. Mater.* **16**, 101–108 (2017).

43. A. C. T. van Duin, S. Dasgupta, F. Lorant, W. A. Goddard, ReaxFF: A reactive force field for hydrocarbons. *J. Phys. Chem. A* **105**, 9396–9409 (2001).

44. F. H. Streitz, J. W. Mintmire, Electrostatic potentials for metal-oxide surfaces and interfaces. *Phys. Rev. B* **50**, 11996–12003 (1994).

45. X. W. Zhou, H. N. G. Wadley, R. A. Johnson, D. J. Larson, N. Tabat, A. Cerezo, A. K. Petford-Long, G. D. W. Smith, P. H. Clifton, R. L. Martens, T. F. Kelly, Atomic scale structure of sputtered metal multilayers. *Acta Mater.* **49**, 4005–4015 (2001).

46. J. Cai, Y. Y. Ye, Simple analytical embedded-atom-potential model including a long-range force for fcc metals and their alloys. *Phys. Rev. B* **54**, 8398–8410 (1996).

47. J. Lindhard, V. Nielsen, M. Scharff, Approximation method in classical scattering by screened coulomb fields (notes on atomic collisions I). *Kgl. Dan. Vidensk. Selsk., Mat.-Fys. Medd.* **36**, 31 (1968).

48. N. S. Smirnov, E. A. Krivko, A. A. Solovyova, A. I. Ivanov, I. A. Rodionov, Wiring surface loss of a superconducting transmon qubit. *Sci. Rep.* **14**, 7326 (2024).

49. I. A. Rodionov, A. S. Baburin, A. R. Gabidullin, S. S. Maklakov, S. Peters, I. A. Ryzhikov, A. V. Andriyash, Quantum engineering of atomically smooth single-crystalline silver films. *Sci. Rep.* **9**, 12232 (2019).

50. L. J. Zeng, S. Nik, T. Greibe, P. Krantz, C. M. Wilson, P. Delsing, E. Olsson, Direct observation of the thickness distribution of ultra thin AlO_x barriers in Al/AlO_x/Al Josephson junctions. *J. Phys. D Appl. Phys.* **48**, 395308 (2015).
